# Modeling gonorrhea vaccination to find optimal targeting strategies that balance impact with cost-effectiveness

**DOI:** 10.1038/s41541-025-01159-0

**Published:** 2025-06-21

**Authors:** Trystan Leng, Lilith K. Whittles, Dariya Nikitin, Peter J. White

**Affiliations:** 1 MRC Centre for Global Infectious Disease Analysis and NIHR Health Protection Research Unit in Modelling and Health Economics, Imperial College London, London, UK; 2 Modelling and Economics Unit, UK Health Security Agency, London, UK

**Keywords:** Public health, Epidemiology, Infectious diseases

## Abstract

Vaccination for UK men who have sex with men (MSM) at increased gonorrhea risk has been advised, but not yet implemented. Effective targeting is essential for cost-effectiveness, but previously-examined approaches have disadvantages: Vaccination-on-Diagnosis has low coverage (limiting impact), and Vaccination-according-to-Risk requires asking about sexual behavior to identify at-risk individuals, which is not always feasible. We developed a transmission-dynamic model to evaluate novel strategies offering vaccination based on information readily available to clinicians (diagnostic/vaccination history, if the patient is seeking care due to partner notification). Offering vaccination to MSM who are notified partners of gonorrhea cases or were diagnosed themselves in the past 2 years averts 1.6x more cases and is more cost-effective than Vaccination-on-Diagnosis. If vaccination provides 20% protection for 1.5 years after primary vaccination and 3 years after revaccination then at £18/dose administered, all considered strategies have ≥50 and ≥90% probabilities of positive net monetary benefit compared with no vaccination with a quality-adjusted life year valued at £20,000 and £30,000 respectively, thus meeting the UK criteria for cost-effectiveness. All novel strategies considered achieve greater impact than Vaccination-on-Diagnosis without the feasibility issues of Vaccination-according-to-Risk.

## Introduction

Gonorrhea is an important public health issue, with over 82 million cases occurring globally in 2020^1^, and there is increasing concern about antimicrobial resistance limiting treatment options^2^. Men who have sex with men (MSM) are a highly-affected group internationally^3–6^, and in England nearly half of all gonorrhea diagnoses are among MSM^7^. The UK’s Joint Committee on Vaccination and Immunisation (JCVI) has advised use of the 4CMenB meningitis B vaccine for MSM with increased risk of gonorrhea acquisition^8,9^, based on extensive observational evidence showing it provides partial protection against gonorrhea, combined with transmission-dynamic modeling^10^. Alongside this, gonorrhea-specific vaccines are in development^11^, which are intended to offer greater protection.

The key question for implementation is how to target vaccination towards those MSM at higher risk, because although offering vaccination to all MSM attending sexual health clinics (SHCs) has the greatest impact (i.e., greatest reduction in cases), it has excessive costs^10,12^. Previously-examined targeting strategies have been “Vaccination-on-Diagnosis” (VoD), where all individuals diagnosed with gonorrhea infection are eligible, and “Vaccination-according-to-Risk” (VaR), where both those diagnosed with gonorrhea infection and those reporting high numbers of sexual partners when attending SHCs for screening are eligible^10,12^. Both have problems. VoD achieves low coverage, limiting its impact. VaR will not always be feasible to implement^10,13^ because it requires information on sexual behavior which is not always known^14,15^ and which may be difficult to accurately elicit due to the sensitivity of the topic^16^.

To examine alternative strategies intended to be more impactful than VoD and still be cost-effective (Table 1) we developed a new model with the novel features of tracking individuals’ past history of gonorrhea diagnosis and representing partner notification, so that use of these markers of risk could be simulated. We evaluate “Vaccination-according-to-History” (VaH), offering vaccination to uninfected SHC patients who have a recent history of gonorrhea, which is associated with increased risk of future diagnoses^15,17^. We also evaluate “Vaccination-according-to-partner-Notification” (VaN), offering vaccination to patients who attend SHC, because they are notified contacts of a person diagnosed with gonorrhea even if they are uninfected (infected patients are already eligible under VoD), as sexual mixing patterns mean that partners of high-risk individuals are also likely to be high-risk. Finally, we evaluate combining VaH and VaN.

**Table 1:** Vaccination strategies considered

Strategy	Eligibility^†^	Rationale	${\delta }_{a,d}^{VoS}$^*^	*δ*^VaN*^	*δ*^VoD*^
Baseline targeting strategy					
Vaccination-on-Diagnosis (VoD)	MSM diagnosed with gonorrhea in SHCs at the time of attendance, both through seeking care for symptomatic infection and through attending for screening (testing in the absence of symptoms).	MSM who are diagnosed with gonorrhea at an SHC are more likely to be at high future risk than uninfected MSM.	0	0	1
Strategies with additional eligibility					
Vaccination-according-to-History (VaH)	MSM attending an SHC for screening who were diagnosed in the previous 1 or 2 years (depending on the eligibility period chosen), in addition to those eligible under VoD (i.e., MSM diagnosed with gonorrhea at the time of attendance).	MSM with a recent history of gonorrhea diagnosis are likely to be at high future risk even if currently uninfected.	0 if *d* = 0 1 if *d* = 1	0	1
Vaccination-according-to-partner- Notification (VaN)	Uninfected MSM attending an SHC because of contact with a partner who has been diagnosed with gonorrhea, in addition to those eligible under VoD (i.e., MSM diagnosed with gonorrhea at the time of attendance).	MSM who have been exposed to infection are likely to be at high future risk even if currently uninfected, particularly if there is assortativity in sexual mixing with high-activity individuals being particularly likely to have sex with other high-activity individuals.	0	1	1
VaH + VaN	MSM attending an SHC for screening who were diagnosed in the previous 1 or 2 years (depending on the eligibility period chosen) + Uninfected MSM attending an SHC because of contact with a partner who has been diagnosed with gonorrhea in addition to those eligible under VoD (i.e., MSM diagnosed with gonorrhea at the time of attendance).	MSM with a recent history of gonorrhea diagnosis and those who have been exposed to infection are likely to be at high future risk even if currently uninfected.	0 if *d* = 0 1 if *d* = 1	1	1
Vaccination-according-to-Risk (VaR)	MSM attending an SHC for screening who report a high number of recent sexual partners (over 5 in the previous year) in addition to those eligible under VoD (i.e., MSM diagnosed with gonorrhea at the time of attendance).	MSM with high numbers of sexual partners are at high future risk of infection	0 if *a* = *L* 1 if *a* = *H*	0	1

*MSM* men who have sex with men, *SHC* sexual health clinic.

^†^For all strategies, vaccination eligibility is restricted to those who have not previously been vaccinated, or have not received both doses required for full primary vaccination, or who received two-dose primary vaccination more than *E*_*V*_ years ago, or who received booster vaccination more than *E*_*R*_ years ago.

*The indicator functions ${\delta }_{a,d}^{VoS}$, *δ*^*VaN*^, and *δ*^*VoD*^ respectively indicate whether vaccination is offered to uninfected individuals attending for screening, to uninfected individuals attending for screening due to partner notification, or to infected individuals when diagnosed.

We tested the robustness of these strategies under a range of scenarios regarding the level and duration of vaccine protection, vaccine cost, population vaccine sentiment, and the time-horizon considered.

## Results

**Fig. 1:**
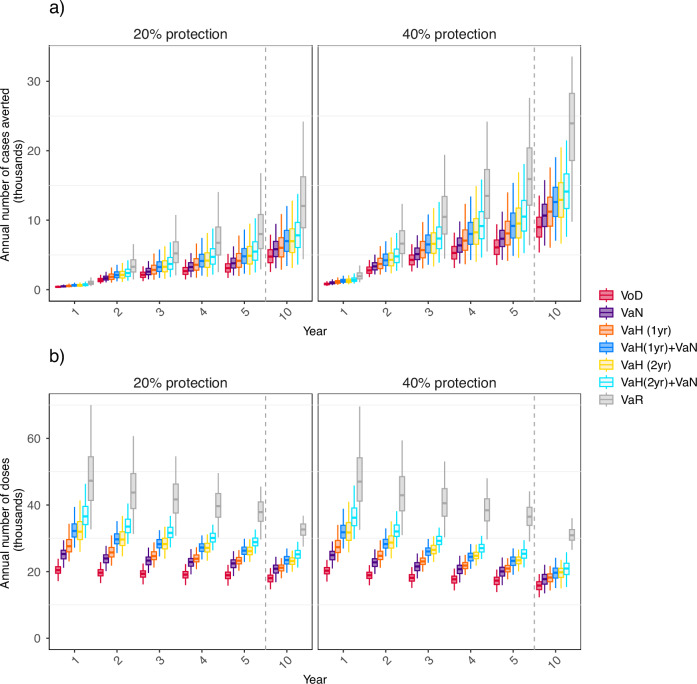
Annual numbers of gonorrhea cases averted and vaccine doses administered over the first 10 years of a vaccination program under different targeting strategies. Vaccination strategies considered are Vaccination-on-Diagnosis (VoD); Vaccination-according-to-partner-Notification (VaN); Vaccination-according-to-History (VaH), offering vaccination to those with a diagnosis in the last year [VaH(1yr)] or in the last 2 years [VaH(2yr)]; VaN combined with VaH(1yr) or VaH(2yr); and Vaccination-according-to-Risk (VaR). Primary vaccination provides protection for 1.5 years, and booster vaccination protects for 3 years. Panels on the left and right show results for the vaccine providing 20 and 40% protection after two-dose primary vaccination, respectively. Section **a** shows the annual numbers of diagnoses averted, and section **b** shows the annual numbers of doses administered. Box plots depict results from 1000 sets of sampled epidemiological and health-economic parameters, with whiskers indicating the 2.5th and 97.5th centiles, boxes the 25th and 75th centiles, and the central line the median (50th centile).

In our main analysis, we consider conservative durations of vaccine protection (1.5 years after primary vaccination and 3 years after revaccination), with longer durations examined in supplementary material. Figure 1a shows the impact of the considered targeting strategies. For all strategies, the annual number of cases averted increases over time as the numbers of individuals with vaccine protection increases, with the most marked increase over the first few years (Fig. 1a). The annual number of cases diagnosed and gonorrhea prevalence decrease over time (Supplementary Fig. 1). The annual number of doses administered decreases over time (Fig. 1b) as coverage increases (Supplementary Fig. 2), with a corresponding decrease in the number of eligible unvaccinated people. A vaccine providing 40% protection averts approximately 90% more cases over 10 years than a vaccine providing 20% protection [e.g., 82,900 (95% CrI:28,800–159,300) vs 154,900(63,200–246,200) for VaR] (Tables 2,3).

**Table 2:** Health-economic analysis of vaccination of MSM in England over 10 years, under different targeting strategies, with a vaccine providing 20% protection after two-dose primary vaccination, for 1.5 years after primary vaccination, and 3 years after booster vaccination

							£18/dose				£85/dose			
Strategy	Cases averted (thousands)		Testing & treatment costs saved (£M)	QALYs gained	Vaccine doses administered (thousands)		Vaccination costs incurred (£M)	Net costs saved (£M)	NMB_£20k_ (£M)	Prob NMB_£20k_ >0	Vaccination costs incurred (£M)	Net costs saved (£M)	NMB_£20k_ (£M)	Prob NMB_£20k_ >0
	Undisc.	Disc.			Undisc.	Disc.			*NMB*_*£30k*_ *(£M)*	*Prob* *NMB*_*£30k*_ *>0*			*NMB*_*£30k*_ *(£M)*	*Prob* *NMB*_*£30k*_ *>0*
VoD	31.3 (16.6,50.1)	25.3 (13.5,40.4)	4.1 (2.1,6.8)	29.8 (14.5,53.5)	188.9 (157.7,220)	160.4 (134.1,186.7)	2.9 (2.4,3.4)	1.2 (−0.9,4)	1.8 (−0.6,5) *2.1 (−0.5,5.4)*	90.6% *93.1%*	13.6 (11.4,15.9)	−9.5 (−12.8,−6.4)	−9 (−12.4,−5.5) *−8.7 (−12.1,−5)*	0% *0%*
VaN	38.1 (20.3,60.4)	30.8 (16.5,48.9)	5 (2.6,8.3)	36.2 (17.9,65.5)	224.5 (187.3,261.5)	191 (159.5,222.8)	3.4 (2.9,4)	1.5 (−1,5)	2.3 (−0.7,6) *2.6 (−0.5,6.5)*	91.7% *93.9%*	16.2 (13.6,18.9)	−11.3 (−15.1,−7.3)	−10.5 (−14.5,−6.3) *−10.2 (−14.3,−5.7)*	0% *0%*
VaH(1yr)	41.4 (19.2,73.4)	33.6 (15.7,59.4)	5.4 (2.4,9.8)	39.5 (17,77.2)	235.2 (204,268.7)	200.7 (173.7,228.5)	3.6 (3.1,4.1)	1.8 (−1,6)	2.6 (−0.7,7.3) *3 (−0.5,8.1)*	91.3% *93.5%*	17.1 (14.8,19.4)	−11.6 (−15,−8)	−10.8 (−14.4,−6.8) *−10.5 (−14.1,−6.1)*	0% *0%*
VaH(1yr) + VaN	47.5 (22.6,81.8)	38.5 (18.4,66.2)	6.2 (2.9,10.9)	45.3 (20.1,87.4)	266.3 (231.4,302.2)	227.6 (197.8,258.1)	4.1 (3.6,4.6)	2.1 (−1.1,6.9)	3 (−0.8,8.3) *3.5 (−0.5,9.2)*	92.3% *94.3%*	19.3 (16.8,21.9)	−13.1 (−17.2,−8.7)	−12.2 (−16.6,−7.3) *−11.8 (−16.4,−6.5)*	0% *0.1%*
VaH(2yr)	48.4 (20.9,87.7)	39.3 (17,71.3)	6.3 (2.6,11.7)	46.1 (18.6,91.6)	265.1 (229.4,303)	226.7 (194.9,260.3)	4.1 (3.5,4.7)	2.3 (−1.1,7.4)	3.2 (−0.8,9) *3.6 (−0.6,9.8)*	91.6% *93.8%*	19.3 (16.6,22.1)	−12.9 (−16.3,−8.9)	−12 (−15.8,−7.4) *−11.5 (−15.5,−6.5)*	0% *0.2%*
VaH(2yr)+ VaN	54 (24.3,94.7)	43.8 (19.7,77.1)	7.1 (3.1,12.9)	51.5 (21.7,101.1)	293.7 (257,332.3)	251.5 (219.7,285.5)	4.5 (4,5.1)	2.6 (−1.2,8.2)	3.6 (−0.8,9.9) *4.1 (−0.5,10.9)*	92.4% *94.5%*	21.4 (18.7,24.3)	−14.3 (−18.5,−9.5)	−13.3 (−17.8,−7.8) *−12.8 (−17.5,−6.9)*	0% *0.2%*
VaR	82.9 (28.8,159.3)	67.1 (23.4,129)	10.8 (3.6,21.4)	78.8 (25.9,163.4)	384 (295.5,476.4)	329 (251.5,411.3)	5.9 (4.5,7.4)	4.9 (−0.9,14.3)	6.5 (−0.4,17.4) *7.3 (−0.2,19.1)*	96.0% *97.1%*	28 (21.4,35)	−17.1 (−20.1,−12)	−15.6 (−18.7,−9.3) *−14.8 (−18.3,−7.6)*	0% *0.1%*

*Undisc* undiscounted, *Disc* discounted, *M* millions, *NMB*_*£20k*_ net monetary benefit with a QALY valued at £20,000, *NMB*_*£30k*_ net monetary benefit with a QALY valued at £30,000 (shown in italic text), *QALY* quality-adjusted life year, *VoD* Vaccination-on-Diagnosis, *VaN* Vaccination-according-to-partner-Notification, *VaH(1yr)* Vaccination-according-to-History (diagnosed in last year), *VaH(2yr)* Vaccination-according-to-History (diagnosed in last 2 years), *VaR* Vaccination-according-to-Risk.

Results are mean values and 95% credible intervals (CrIs) of simulations comparing each vaccination strategy against no vaccination, using 1000 sets of sampled epidemiological and health-economic parameters. All values are discounted at 3.5% per annum except where stated. Note that at £18/dose administered, each strategy gains more QALYs and saves more money than the one above, and therefore dominates the one above.

**Table 3:** Health-economic analysis of vaccination of MSM in England over 10 years, under different targeting strategies, with a vaccine providing 40% protection after two-dose primary vaccination, for 1.5 years after primary vaccination, and 3 years after booster vaccination

							£18/dose				£85/dose			
Strategy	Cases averted (thousands)		Testing & treatment costs saved (£M)	QALYs gained	Vaccine doses administered (thousands)		Vaccination costs incurred (£M)	Net costs saved (£M)	NMB_£20k_ (£M)	Prob NMB_£20k_>0	Vaccination costs incurred (£M)	Net costs saved (£M)	NMB_£20k_ (£M)	Prob NMB_£20k_>0
	Undisc.	Disc.			Undisc.	Disc.			*NMB*_*£30k*_ *(£M)*	*Prob* *NMB*_*£30k*_*>0*			*NMB*_*£30k*_ *(£M)*	*Prob* *NMB*_*£30k*_*>0*
VoD	59.8 (35.1,89.7)	48.5 (28.6,72.6)	7.8 (4.3,12.3)	57 (30.1,99.4)	173.7 (140.6,206.1)	148 (120.1,175)	2.7 (2.2,3.1)	5.2 (1.5,9.8)	6.3 (2.2,11.6) *6.9 (2.5,12.6)*	100% *100%*	12.6 (10.2,14.9)	−4.7 (−9.4,0.6)	−3.6 (−8.7,2.2) *−3 (−8.3,3.1)*	10.4% *15.3%*
VaN	71.5 (42.3,106.4)	58.1 (34.4,86.4)	9.4 (5.3,14.7)	68.2 (36.7,117.9)	202.7 (163,242.4)	173.3 (139.9,206.6)	3.1 (2.5,3.7)	6.3 (1.9,11.7)	7.6 (2.8,13.8) *8.3 (3.2,14.5)*	100% *100%*	14.7 (11.9,17.6)	−5.4 (−10.9,1)	−4 (−10,2.8) *−3.3 (−9.6,4)*	11.7% *16.8%*
VaH(1yr)	78.2 (40.7,127.5)	63.6 (33.1,103.7)	10.3 (5.1,17.2)	74.6 (35.5,138)	213.8 (183.7,243.6)	183.2 (158.5,208.9)	3.3 (2.9,3.8)	7 (1.8,13.9)	8.5 (2.5,16.4) *9.2 (2.9,17.6)*	100% *100%*	15.6 (13.5,17.8)	−5.3 (−10.8,1.7)	−3.8 (−9.9,4.2) *−3.1 (−9.5,5.4)*	14.8% *20.2%*
VaH(1yr) + VaN	88.2 (47.6,139.1)	71.8 (38.8,113.4)	11.6 (6,18.9)	84.2 (41.8,150.8)	238 (202.4,276)	204.5 (174.9,236.1)	3.7 (3.1,4.3)	7.9 (2.2,15.5)	9.6 (3.1,17.9) *10.4 (3.5,19.1)*	100% *100%*	17.4 (14.9,20.1)	−5.8 (−12.2,2.1)	−4.1 (−11.1,4.7) *−3.3 (−10.6,6.1)*	15.6% *21.5%*
VaH(2yr)	91.2 (44.7,151.8)	74.2 (36.4,123.9)	12 (5.7,20.3)	87.1 (39.4,164)	240 (210.1,270.6)	206.3 (180.4,233)	3.7 (3.2,4.2)	8.3 (1.9,16.8)	10 (2.8,19.5) *10.9 (3.1,21.1)*	100% *100%*	17.5 (15.3,19.8)	−5.6 (−11.8,2.8)	−3.8 (−10.8,5.8) *−2.9 (−10.2,7.2)*	18.3% *24.6%*
VaH(2yr) + VaN	100.2 (51.5,161.4)	81.6 (42,131.4)	13.2 (6.5,21.9)	95.7 (45.1,176.1)	261.8 (226.9,297.8)	225.4 (197,254.8)	4.1 (3.5,4.6)	9.1 (2.4,18.1)	11 (3.3,20.8) *12 (3.7,22.4)*	100% *100%*	19.2 (16.7,21.7)	−6 (−13,3.1)	−4.1 (−11.8,6.3) *−3.1 (−11.3,7.9)*	18.6% *25.9%*
VaR	154.9 (63.2,246.2)	125.7 (51.4,201)	20.3 (8,34.5)	147.5 (57.1,272.5)	371.9 (289.6,464.6)	319 (246.5,401.5)	5.7 (4.4,7.2)	14.5 (3.5,27.6)	17.5 (4.7,32.1) *19 (5.3,34.7)*	100% *100%*	27.1 (21,34.1)	−6.8 (−13.4,2.6)	−3.9 (−12.1,7) *−2.4 (−11.5,9.2)*	21.4% *30.9%*

*Undisc* undiscounted, *Disc* discounted, *M* millions, *NMB*_*£20k*_ net monetary benefit with a QALY valued at £20,000, *NMB*_*£30k*_ net monetary benefit with a QALY valued at £30,000 (shown in italic text), *QALY* quality-adjusted life year, *VoD* Vaccination-on-Diagnosis, *VaN* Vaccination-according-to-partner-Notification, *VaH(1yr)* Vaccination-according-to-History (diagnosed in last year), *VaH(2yr)* Vaccination-according-to-History (diagnosed in last 2 years), *VaR* Vaccination-according-to-Risk.

Results are mean values and 95% credible intervals (CrIs) of simulations comparing each vaccination strategy against no vaccination, using 1000 sets of sampled epidemiological and health-economic parameters. All values are discounted at 3.5% per annum except where stated. Note that at £18/dose administered, each strategy gains more QALYs and saves more money than the one above, and therefore dominates the one above.

The novel vaccination strategies that we examined all have a greater impact than VoD, as more people are offered vaccination. Of these, VaN has the smallest increase over VoD, with VaH having a greater impact, which increases when considering a 2-year history rather than a 1-year history (denoted VaH(2yr) and VaH(1yr) respectively). Combining VaN and VaH has a greater impact still. However, VaR has a considerably larger impact than all other strategies considered. For example, if vaccine protection is 20% then VaR averts 82,900(95% CrI: 28,800–159,300) cases over 10 years, compared to 54,000(24,300–94,700) cases averted by VaH(2yr) + VaN, which is the next-highest-impact strategy (Table 2), compared to 31,300 (16,600–50,100) cases averted by VoD alone. Note that the uncertainty in the estimates is primarily due to uncertainty in epidemiological parameters; there is little uncertainty in the relative effectiveness of the different strategies.

**Fig. 2:**
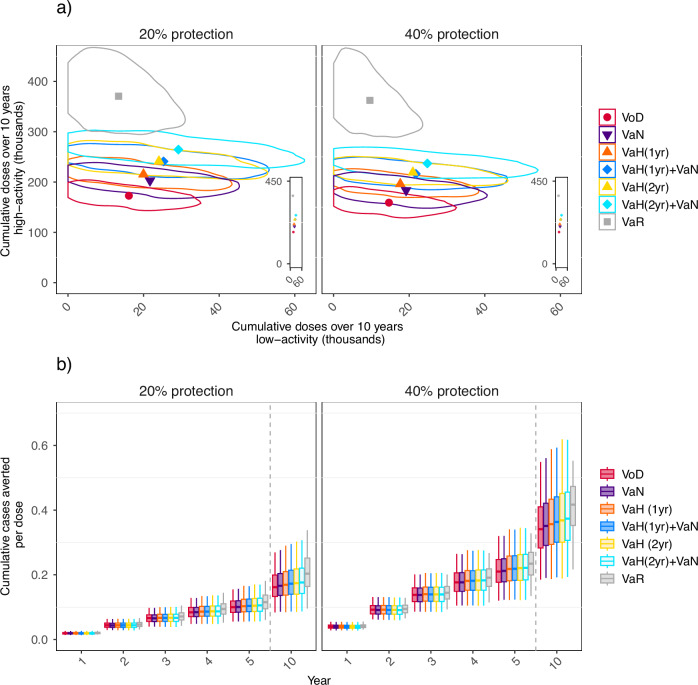
Risk profile of vaccinated individuals and vaccination efficiency under different vaccination strategies over the first 10 years of a vaccination program under different targeting strategies. Vaccination strategies considered are Vaccination-on-Diagnosis (VoD); Vaccination-according-to-partner-Notification (VaN); Vaccination-according-to-History (VaH), offering vaccination to those with a diagnosis in the last year [VaH(1yr)] or in the last 2 years [VaH(2yr)]; VaN combined with VaH(1yr) or VaH(2yr); and Vaccination-according-to-Risk (VaR). Section **a** Cumulative number of doses administered to low-activity men who have sex with men (MSM) against doses administered to high-activity MSM. Note the very different magnitudes of the horizontal and vertical scales; inset plots show the same mean values, but with the same scale on both axes. Point markers show mean values, while outlined regions show high-density regions which include 80% of model results^54^ VoD is shown with a circle marker, VaN is shown with a triangle pointing downwards, VaH(1yr) and VaH(2yr) are shown with a triangle pointing upwards, VaH(1yr) + VaN and VaH(2yr) + VaN are shown with diamond markers, and VaR is shown with a square marker. Section **b** the cumulative number of cases averted per dose administered. All plots use 1000 sets of sampled epidemiological and health-economic parameters. Box plot whiskers indicate the 2.5th and 97.5th centiles, boxes the 25th and 75th centiles, and the central line the median (50th centile).

Vaccinating high-activity individuals reduces transmission more than vaccinating low-activity individuals, so is a more-efficient use of resources. We examined the number of vaccine doses administered to each activity group under the different strategies. High-activity MSM receive the majority of doses under all strategies, but strategies differ in the coverage they achieve and the proportion of vaccinees who are high-activity individuals. VaR vaccinates the most high-activity individuals and the fewest low-activity individuals (Fig. 2a). VoD vaccinates the fewest individuals in total and the fewest high-activity individuals, but actually vaccinates more low-activity individuals than VaR. This is because VoD achieves the smallest reduction in transmission, so more low-activity individuals become infected under VoD, and are consequently eligible for vaccination.

Strategies involving VaN and VaH vaccinate more low- and high-activity individuals than VoD. For all strategies, the ratio of gonorrhea cases averted to vaccine doses administered (Fig. 2b) increases over time, as reductions in prevalence lead to reductions in incidence. The ratio is highest for VaR, as this is the most successful strategy for targeting high-activity individuals. All VaN and VaH strategies considered have higher ratios than VoD (Fig. 2b).

**Fig. 3:**
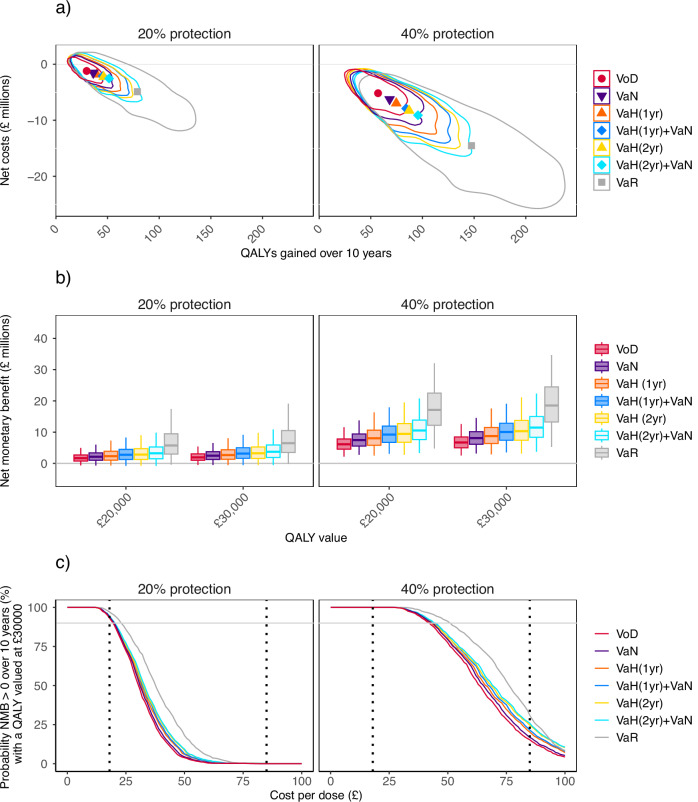
Cost-effectiveness over the first 10 years of a vaccination program under different targeting strategies. Vaccination strategies considered are Vaccination-on-Diagnosis (VoD); Vaccination-according-to-partner-Notification (VaN); Vaccination-according-to-History (VaH), offering vaccination to those with a diagnosis in the last year [VaH(1yr)] or in the last 2 years [VaH(2yr)]; VaN combined with VaH(1yr) or VaH(2yr); and Vaccination-according-to-Risk (VaR). Section **a** Cost-effectiveness planes, for a vaccine costing £18/dose administered. The x-axis shows quality-adjusted life years gained by each strategy over 10 years, while the y-axis shows net costs of each strategy over 10 years (negative costs indicate that savings in testing and treatment outweigh vaccination costs). VoD is shown with a circle marker, VaN is shown with a triangle pointing downwards, VaH(1yr) and VaH(2yr) are shown with a triangle pointing upwards, VaH(1yr) + VaN and VaH(2yr) + VaN are shown with diamond markers, and VaR is shown with a square marker. Point markers show mean values, while outlined regions show high-density regions, which include 80% of model results^54^. Section **b** Box plots of the net monetary benefit of each strategy over 10 years, for a vaccine costing £18/dose administered and with a QALY valued at £20,000 or £30,000. Box plot whiskers indicate the 2.5th and 97.5th centiles, boxes the 25th and 75th centiles, and the central line the median (50th centile). Section **c** Probability that vaccination is cost-effective (i.e., net monetary benefit is positive, compared to no vaccination) at different costs per dose administered, with a QALY valued at £30,000. All plots use 1000 sets of sampled epidemiological and health-economic parameters.

At £18/dose administered, savings in testing and treatment considerably outweigh vaccination costs for all strategies, so there is a net saving (i.e., negative net costs) (Fig. 3a). The net monetary benefit of vaccination (NMB) accrues more from cost savings than gains in quality-adjusted life years (QALYs) (Tables 2,3), with the NMB with a QALY valued at £30,000 (NMB_ £30k_) being only slightly greater than NMB_ £20k_ for all strategies (Fig. 3b). Therefore, the requirement for cost-effectiveness that NMB_ £30k_ be positive with at least 90% probability is more stringent than the requirement that NMB_ £20k_ be positive with at least 50% probability. So, we examine how the cost per dose administered affects the probability that vaccination is cost-effective using NMB_ £30k_ (Fig. 3c). We present results for NMB_ £20k_ in Supplementary Fig. 6.

If vaccine protection is 20% then VoD (the lowest-impact strategy) averts 31,300 (95% CrI:16,600–50,100) (undiscounted) cases and saves £1.2M (95% CrI:-£0.9M–£4.0M) (discounted) over 10 years at £18/dose administered, VaH(2yr) + VaN (the highest-impact strategy not requiring information on sexual behavior) averts 54,000 (24,300–94,700) cases and saves £2.6M (-£1.2M–£8.2M), and VaR (the highest-impact strategy) averts 82,900 (28,800–159,300) cases and saves £4.9M (-£0.9M–£14.3M) (Table 2). Corresponding values for 40% protection are VoD 59,800 (35,100–89,700) cases, £5.2M (£1.5M–£9.8M); VaH(2yr) + VaN 100,200 (51,500–161,400) cases, £9.1M (£2.4M–£18.1M); VaR 154,900 (63,200–246,200) cases, £14.5M (£3.5M–£27.6M) (Table 3).

If vaccine protection is 20% then at £18/dose administered all strategies meet the two UK cost-effectiveness criteria (NMB_ £20k_ > 0 with at least 50% probability and NMB_ £30k_ > 0 with at least 90% probability) over 10 years (Fig. 3c, Tables 2, 3, and Supplementary Fig. 6) but the probability of being cost-effective declines sharply if the cost exceeds £18/dose (Fig. 3c). If protection is 40% then vaccination remains cost-effective at higher costs: up to £50/dose administered for VaR, £44/dose for VaH(1yr) + VaN and VaH(2yr) + VaN, £43/dose for VaH(2yr) and VaN, and £42/dose for VaH(1yr) and VoD. Notably, VaR and VaH(2yr) + VaN meet the two UK cost-effectiveness criteria when compared to any of the lower-impact strategies considered (incremental analysis comparing strategies to lower-impact strategies is shown in Supplementary Tables 1, 2). At £85/dose administered no strategy meets the cost-effectiveness criteria over 10 years (Tables 2, 3, and Supplementary Fig. 5).

The relationship between NMB and cost per dose is shown in Supplementary Fig. 3a, showing that NMB decreases linearly with cost per dose. The expected loss associated with each strategy, i.e., the expected difference in NMB between the implemented strategy and the strategy with the highest NMB, is shown in Supplementary Fig. 3b. The probability that each strategy has the highest NMB of all strategies as cost per dose is varied is shown in Supplementary Fig. 4. If VaR is included then either VaR or no vaccination has the highest probability of having the highest NMB. If VaR is excluded then either VaH(2yr) + VaN or no vaccination has the highest probability of having the highest NMB.

Fewer QALYs are gained using an alternative, lower, estimate for the disutility of symptomatic gonorrhea^18^, but cost-effectiveness results remain qualitatively similar, as the bulk of benefit of vaccination comes from savings in testing and treatment (Supplementary Tables 3, 4).

Each strategy has a higher NMB when evaluated over 20 years (Supplementary Fig. 7 and Supplementary Tables 5, 6), but still none meets the cost-effectiveness criteria at £85/dose even if protection is 40% (Supplementary Table 6 and Supplementary Fig. 7).

If vaccine protection lasts for 4 or 7.5 years then vaccination has a greater impact and is more likely to be cost-effective at higher costs per dose (Supplementary Fig. 8 and Supplementary Tables 7–11). Vaccine protection of 40% lasting for 7.5 years would almost meet the UK’s cost-effectiveness criteria over 10 years under all targeting strategies at £85/dose administered (all have over 89% probability that NMB_ £30k_ > 0).

Finally, in the alternative vaccine-sentiment scenario in which a proportion of MSM are unwilling to be vaccinated, each strategy has a lower impact (ranging from 25–43% fewer cases averted over 10 years), due to lower coverage being reached; however, as this also means lower costs of vaccination, cost-effectiveness results were very similar to our main scenario (Supplementary Fig. 10 and Supplementary Tables 12, 13).

## Discussion

In this paper we examined MSM vaccination approaches which do not require enquiring about sexual behavior (as the “Vaccination-according-to-Risk” (VaR) strategy does), and which achieve greater coverage specifically of higher-risk individuals than the “Vaccination-on-Diagnosis” (VoD) strategy of only offering vaccination to patients diagnosed with gonorrhea at SHC attendance. We find the most impactful and cost-effective alternative strategy to VaR is offering vaccination to patients meeting any of the criteria of i) being diagnosed with gonorrhea on attendance, or ii) having a recent history of gonorrhea, or iii) attending as notified partners of gonorrhea cases.

Each strategy we considered meets both UK cost-effectiveness criteria over a 10-year time horizon if vaccination costs £18/dose administered and provides at least 20% protection for 1.5 years following primary vaccination and 3 years after booster vaccination. Higher levels and longer durations of protection, and considering a longer time-horizon, all increase the health gains of vaccination and the cost savings from averted costs of diagnosis and treatment. Therefore, these factors could make vaccination cost-effective at higher costs per dose administered. This is particularly relevant to new vaccines that are in development and may come to market in the next few years^11^. In the supplementary material, we present graphs (Supplementary Fig. 8) that can inform policymakers regarding the value of vaccines with different properties.

A key strength of our study is that, by tracking time since the last diagnosis and quantifying the number of patients attending SHCs due to partner notification, we could evaluate for the first time alternative vaccination strategies that can be implemented at SHCs using readily available information. Our modeling suggests that strategies offering vaccination using this information effectively target a high-activity cohort (Fig. 2). These strategies have the benefit of not relying on self-reported sexual behavior, which may be difficult to elicit and may not always be accurate^16^, and of course the success of VaR relies on the accuracy of self-reported behavior.

Another strength is that we ensured our results are robust to uncertainty in epidemiological parameters, including those relating to the natural history of *Neisseria gonorrhoeae*, patterns of sexual mixing between low- and high-activity groups (which affects how many notified partners of high-activity individuals are also high-activity individuals), rates of SHC attendance due to partner notification, and in gonorrhea prevalence among those notified partners. Additionally, we ensured our results are robust to uncertainty in population vaccine sentiment, by examining two alternative interpretations of vaccine-uptake data.

There is uncertainty in the level and duration of protection provided by vaccines (both 4CMenB and gonorrhea-specific vaccines in development), so we explored combinations of these factors. Recently the DOXYVAC trial reported an adjusted hazard ratio of 0.78 (95% CI:0.60–1.01; *p* = 0.061) for 4CMenB^19^, corresponding to 22% protection if it were statistically significant. However, the trial was powered for 30% protection and was stopped early due to an error in the interim analysis, further reducing its power^19^. Multiple observational studies have estimated that 4CMenB provides protection against gonorrhea in the range 33–47%^9,20–24^, and other clinical trials of 4CMenB protection against gonorrhea are in progress^9^. Gonorrhea-specific vaccines in development are intended to be more protective. Our primary analysis (assuming 1.5 years after primary vaccination and 3 years after revaccination) is conservative with regard to the duration of protection of 4CMenB, as evidence indicates protection may last at least 4 years or even at least 7.5 years^25^. Studies quantifying a vaccine’s duration of protection would enable us to refine estimates of its value, and would inform the frequency of repeat vaccination required to maintain population protection^26^.

Whilst the level and duration of protection obviously affect the value of a vaccine—i.e., the maximum cost per dose administered at which it would be cost-effective to use—the rank-order of cost-effectiveness of the targeting strategies we examined is the same under all scenarios for which vaccination is cost-effective (i.e., at 20 or 40% protection for all three durations of protection for a vaccine costing £18/dose administered). Using this scenario-based approach, our results demonstrate that even a vaccine with 20% protection lasting for only 1.5 years after primary vaccination and 3 years would be cost-effective at £18/dose administered, and should be taken as evidence that a vaccine with 20–40% efficacy with a duration of protection between 1.5 and 7.5 years after primary vaccination and between 3 and 7.5 years after booster vaccination is very likely to be cost-effective (for the vaccination strategies considered) at this cost.

Our analysis is conservative because our model does not quantify the benefit of vaccination in combating antimicrobial resistance (AMR) in gonorrhea, due to a lack of suitable data on both the health burden and costs, but these could potentially become very large^6^. Recently, high prevalence of ‘extensively drug-resistant’ gonorrhea has been observed in Cambodia^27^, and in the UK there has been several cases of ceftriaxone-resistant infection^28^. Vaccination reduces prevalence, thus reducing *Neisseria gonorrhoeae*’s opportunities for mutation and genetic exchange, and reduces the need for treatment, thus reducing antibiotic selection pressure^29^. The World Health Organization has identified vaccination against gonorrhea (and other STIs) as “a priority for long-term, sustainable STI control”^30^. We have previously explored the theoretical impact of vaccination in combating gonorrhea AMR^31^, but further progress requires more empirical data, including to estimate evolutionary fitness costs and benefits of resistance^32^.

Moreover, our analysis may also underestimate the full benefit of vaccination because it does not account for the secondary impact of reducing gonorrhea incidence on the incidence of HIV. Previous studies have suggested that gonorrhea infection may increase HIV risk^33^. Whilst including HIV infections averted would be unlikely to have a large impact on cost-effectiveness results in this study, due to low HIV incidence among UK MSM^34^, this may be an important factor to consider in settings with higher HIV incidence rates.

While our model was stratified by sexual activity, our model was not stratified by age. Gonorrhea incidence rates vary by age, and including age as a factor in triage may increase the efficiency and cost-effectiveness of vaccination. However, we note that relevant data to inform sexual behavior parameters disaggregated by age for MSM are sparse, limiting the ability to parameterize an age-structured model.

Our model is not stratified by anatomical site, and we have previously highlighted the need for data on natural history and the effects of vaccination at each site to allow models with this additional level of detail to be parameterized robustly^10,29,35,36^. The proportion of infections that are symptomatic differs by anatomical site, and vaccination might have the effect of reducing symptoms—which prompt care-seeking—and hence prolonging infection, which would tend to promote transmission and would offset to some extent the effects of vaccination in reducing acquisition and promoting clearance of infection^37^.

This paper considers the MSM population, which is the group with the highest gonorrhea incidence in England^6^ and was the main focus for JCVI’s advice. Further work is required to determine if vaccination of heterosexual individuals could be cost-effective. Given that targeting is necessary for vaccination to be cost-effective in MSM, the much lower average incidence in the heterosexual population^7^ means that a highly-targeted approach is likely necessary. One modeling study has evaluated the potential impact of untargeted adolescent 4CMenB vaccination on heterosexual gonorrhea in England^38^ but did not evaluate cost-effectiveness. Further studies are required to inform decisions about heterosexual vaccination, including better quantification of the burden of sequelae in women and men^39^.

In summary, we have identified pragmatic strategies for targeting of gonorrhea vaccination to higher-risk MSM which achieve greater coverage—and therefore greater impact—than VoD but which are still cost-effective and which do not require obtaining sensitive information about sexual behavior that the VaR strategy needs. These strategies involve offering vaccination to those diagnosed with gonorrhea either at the time of attendance or in the recent past, as well as notified partners of gonorrhea cases, making use of readily available information for effective targeting of vaccination toward higher-risk individuals.

## Methods

### Model structure

We developed a new model of gonorrhea transmission and vaccination, building on previous work^10,12^ which represents England’s sexually active MSM population, stratified by sexual behavior, current infection status, history of gonorrhea diagnosis, and vaccination status/vaccine sentiment. The model accounts for rates of SHC attendance due to partner notification.

Within the model, individuals who are uninfected (*U*) can become infected through sexual contact with a contagious individual. Newly-infected individuals pass through an incubating state (*I*), before developing symptoms (*S*), or remaining asymptomatic (*A*). Infected individuals who attend an SHC (either due to care-seeking for symptoms or asymptomatic screening) are treated and enter a treatment state (*T*). Treated individuals are cured, returning to the *U* state; infection does not confer natural immunity, and previously-infected individuals are as susceptible as those never infected.

Heterogeneity in sexual behavior is represented by low (*L*) and high (*H*) sexual activity groups; high-activity MSM have more partners and attend SHCs for screening more frequently.

The population is stratified by vaccination status/sentiment. Individuals who are fully-vaccinated (*V*_*i*_, *V*_*e*_) or revaccinated (*R*_*i*_, *R*_*e*_) have lower rates of acquisition than partially-vaccinated individuals (*P*), who have lower rates of acquisition than those who are unvaccinated (*X*) and those whose vaccine protection has waned (*W*). We also consider, in supplementary analysis, a scenario in which a proportion of individuals are unwilling to be vaccinated (*H*).

In order to consider vaccination strategies in which vaccination is offered to those with a clinical history of gonorrhea, the model distinguishes between individuals diagnosed in the last *D* years (represented by *d* = 1) from those with no previous diagnosis or who were last diagnosed more than *D* years ago (represented by *d* = 0). All treated individuals move into the *U* state in the “Diagnosed in the last *D* years” (*d* = 1) stratum. Over time, individuals in the “Diagnosed in the last *D* years” stratum move into the corresponding infection state in the “Not diagnosed in the last *D* years” (*d* = 0) stratum.

Eligibility for revaccination is dependent upon the time since the last vaccination. We assume that to allow for variation in the duration of protection, repeat vaccination is offered to individuals after a period shorter than the average duration of protection. The model distinguishes between recently vaccinated individuals (*V*_*i*_), who are ineligible for revaccination, and vaccinated individuals who were vaccinated more than *E*_*V*_ years ago (*V*_*e*_), who are eligible for revaccination despite still being vaccine-protected. As a two-dose primary vaccination provides protection for *D*_*V*_ years, individuals remain in *V*_*e*_ for a period of (*D*_*V*_-*E*_*V*_) years before their protection wanes. In the same vein, the model distinguishes between recently revaccinated individuals (*R*_*i*_, ineligible) and revaccinated individuals who received their booster vaccine more than *E*_*R*_ years ago (*R*_*e*_, eligible). As booster vaccination provides protection for *D*_*R*_ years, individuals remain in *R*_*e*_ for a period of (*D*_*R*_-*E*_*R*_) years before their protection wanes. We assume that individuals who only received one primary dose are offered another course of two-dose primary vaccination (according to the implemented vaccination strategy), and those who accept can receive either one or two further doses. Those in *P* who receive one dose remain in *P*, while those who receive two doses move to *V*_*i*_.

**Fig. 4:**
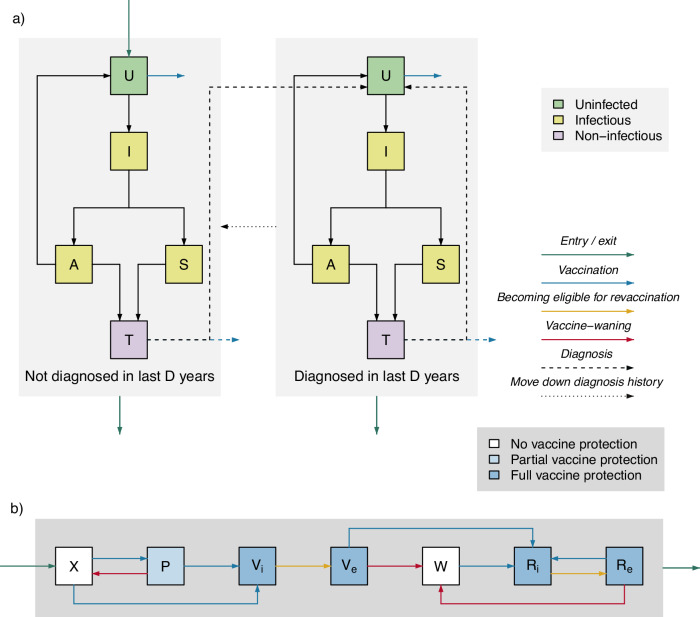
Model structure diagram. The population is divided into compartments representing different states of infection, and stratified according to diagnosis history and vaccination status. Note that there are separate sets of compartments for low and high sexual activity groups, which have identical arrangements of compartments and vaccination/diagnosis history strata. **a** Infection states and stratification by diagnosis history. Individuals enter the population uninfected (*U*); those leaving through aging leave from any state. Uninfected individuals can acquire infection, passing through an incubating state (*I*) before either developing symptoms (*S*) or remaining asymptomatic (*A*). Symptomatic individuals seek treatment due to symptoms, while a proportion of asymptomatic infections are identified via screening. Diagnosed individuals enter the treatment state (*T*). Treated individuals and untreated asymptomatic individuals who recover naturally return to *U*. Those diagnosed in the last *D* years (where *D* = 1 or 2) are distinguished from those never diagnosed or who were last diagnosed over *D* years ago. All treated individuals move into *U* in the “Diagnosed in the last *D* years” stratum. Over time, individuals in the “Diagnosed in the last *D* years” stratum move into the corresponding infection state in the “Not diagnosed in the last *D* years” stratum. Under all considered vaccine-targeting strategies, vaccination is offered to unprotected individuals diagnosed with (and treated for) gonorrhea. Under some strategies, vaccination is also offered to some uninfected individuals at screening (criteria in Table 1). Those who accept enter *U* in a vaccine-protected stratum. **b** Shows stratification according to vaccination status: unvaccinated (*X*), partially-vaccinated (*P*), fully-vaccinated (split into those who are ineligible for revaccination due to recent vaccination, *V*_*i*_, and those who are eligible for revaccination, *V*_*e*_), waned (*W*), and revaccinated (split into those who are ineligible due to recent revaccination, *R*_*i*_, and those who are eligible for repeat vaccination, *R*_*e*_). Unvaccinated individuals in *X* who accept vaccination and receive one dose enter *P*, those who accept vaccination and receive two doses enter *V*_*i*_, and those who decline vaccination remain in *X*. Individuals in *P* remain eligible for two-dose primary vaccination, those who choose to receive only one dose remain in *P* and those who receive two doses enter *V*_*i*_. Individuals in *V*_*i*_ become eligible for single-dose booster vaccination after a period of *E*_*V*_ years, moving to *V*_*e*_. When vaccine protection wanes, partially vaccinated individuals return to *X*, while fully vaccinated individuals in *V*_*e*_ enter *W*. Individuals in *V*_*e*_ and *W* who accept single-dose booster vaccination enter *R*_*i*_. Individuals in *R*_*i*_ become eligible for booster vaccination after a period of *E*_*R*_ years, moving to *R*_*e*_. When protection from booster vaccination wanes, individuals move from *R*_*e*_ back to *W*.

Figure 4 shows a diagram of the model’s structure, and the meaning and value of model parameters are detailed in Table 4. We denote the number of individuals in infection state *Z*, activity group *a*, vaccination status *v*, and diagnosis history *d* at time *t* as ${Z}_{a}^{v,d}(t)$ (or simply ${Z}_{a}^{v,d}$ when context is clear). The model is described by Equations (1–5).

**Table 4:** Model parameters

	Definition	Value	Source
	*Demography*		
*N*	Size of the MSM population in England	600,000	^10,31^
*α*	Annual population entrants (at age 15)	12,000	^48^
$\frac{1}{\gamma }$	Years spent in a sexually active population	50	Ages 15-65
	*Sexual behavior*		
*q*_*L*_	Proportion of the population in activity group *L*	85%	^49^
*q*_*H*_	Proportion of the population in activity group *H*	15%	1 − *q*_*L*_
*c*_*L*_	Annual rate of partner change in activity group *L*	0.6	^10,31^
*c*_*H*_	Annual rate of partner change in activity group *H*	15.6	^10,31^
*ϵ*	Level of assortativity in sexual mixing	0.570 (95% CrI: 0.040, 0.986)	^10^
	*Transmission and natural history*		
*β*	Probability of transmission per-partnership	0.410 (95% CrI: 0.266, 0.650)	^10^
*ψ*	Probability that an incident infection is symptomatic	0.150 (95% CrI: 0.074, 0.238)	^10^
*σ*	Rate of leaving incubating state (*I* → *S* or *A*)	99.9 (95% CrI: 56.1,176.0)	^10^
*μ*	Rate of seeking treatment due to symptoms (*S* → *T*)	218 (95% CrI: 93, 521)	^10^
*ν*	Rate of natural recovery (*A* → *U*)	3.08 (95% CrI: 1.60, 6.03)	^10^
	*Screening and treatment*		
*η*_*L*_	Rate of asymptomatic screening in activity group *L*	0.319 (95% CrI: 0.328, 0.384)	^10,12^
*η*_*H*_	Rate of asymptomatic screening in activity group *H*	0.717 (95% CrI: 0.398, 1.165)	^10,12^
*ρ*	Rate of recovery after treatment (*T* → *U*)	54.0 (95% CrI: 43.6, 66.4)	^10^
	*History of gonorrhea*		
*D*	Length of clinical history considered for VaH	1 or 2 years	-
	*Partner notification*		
*κ*	Number of notified partners per diagnosis	0.39 (95% CrI:0.31, 0.47)	^7^
*p*	gonorrhea prevalence among notified partners	0.38 (95% CrI:0.31, 0.45)	^41^
	*Vaccine properties*		
*e*_*v*_	Protection from full (and booster) vaccination	20 or 40%	^10^
*e*_*p*_	Protection from partial vaccination	2/3 of full protection	^20^
*D*_*P*_	Duration of protection: partial primary vaccination	Main: 1.5 years Supp Fig. 8: 4 years and 7.5 years	^50^
*D*_*V*_	Duration of protection: full primary vaccination	Main: 1.5 years Supp Fig. 8: 4 years and 7.5 years	^25^
*D*_*R*_	Duration of protection: booster vaccination	Main: 3 years Supp Fig. 8: 4 years and 7.5 years	^25^
	*Period ineligible for revaccination*		
*E*_*V*_	Duration ineligible for revaccination after full primary vaccination	Main: 1 year Supp Fig. 8: 3 years and 6 years	-
*E*_*R*_	Duration ineligible for revaccination after booster vaccination	Main: 2 years Supp Fig. 8: 3 years and 6 years	-
	*Vaccine uptake*		
(1 − *h*)	Proportion of the population willing to be vaccinated	Main: 100% Supp Fig. 10: 40.8% (95% CrI: 40.6, 41.0)	^42^
*r*_1_	Proportion accepting (i.e., receiving) the first primary dose when offered	Main: 40.8% (95% CrI: 40.6, 41.0) Supp Fig. 10: 100% in *X*, 0% in *H*	^42^
*r*_2_	Proportion accepting (i.e., receiving) the second primary dose when offered	Main: 61.7% (95% CrI: 61.2, 62.1) Supp Fig. 10: 61.7% (95% CrI: 61.2, 62.1) Supp Fig. 10: in *X*, 0% in *H*	^42^
*r*_*b*_	Proportion accepting (i.e., receiving) booster dose when offered	100%	

The terms *α*_*v*_ (the rate of new entrants into the population entering vaccination-status stratum *v*), *r*_*v*_ (the probability of accepting vaccination and moving between vaccination-status strata), *β*_*v*_ (the per-partnership probability of gonorrhea acquisition), and ${\theta }_{a,v,d}^{Z,wane}$ (the rate of moving between vaccination-status strata due to vaccination waning) take different formulations for different vaccination-status strata.

Dependent on the vaccination-targeting strategy implemented, vaccination is offered to uninfected individuals attending for screening (who were not notified partners), to uninfected individuals attending for screening due to partner notification, or to infected individuals when they are diagnosed. The rates of movement between vaccination-status strata by these are denoted by ${\theta }_{a,v,d}^{VoS}$, ${\theta }_{a,v,d}^{VaN}$, and ${\theta }_{a,v,d}^{VoD}$ respectively, which take different formulations for different vaccination strata and depend upon the vaccination strategy implemented. Terms that differ between vaccination strata are detailed in Equations (6–12).

1 $$\frac{d{U}_{a}^{v,d}}{dt}=\left\{\begin{array}{l}{q}_{a}{\alpha }_{v}+{\theta }_{a,v,d}^{U,wane}-({\lambda }_{a}^{v}+\gamma ){U}_{a}^{v,d}+\nu {A}_{a}^{v,d}+{r}_{v}({\theta }_{a,v,d}^{VoS}+{\theta }_{a,v,d}^{VaN})+\frac{1}{D}{U}_{a}^{v,1}\,{\text{, if}}\,d=0\quad \\ {\theta }_{a,v,d}^{U,wane}-({\lambda }_{a}^{v}+\gamma ){U}_{a}^{v,d}+\nu {A}_{a}^{v,d}+{r}_{v}({\theta }_{a,v,d}^{VoS}+{\theta }_{a,v,d}^{VaN}+\mathop{\sum}\limits _{d^{\prime} }{\theta }_{a,v,d{\prime} }^{VoD})-\frac{1}{D}{U}_{a}^{v,d}+\rho \mathop{\sum}\limits _{d^{\prime} }{T}_{a}^{v,d{\prime} }\,{\text{, if}}\,d=1\quad \end{array}\right.$$

2 $$\frac{d{I}_{a}^{v,d}}{dt}=\left\{\begin{array}{l}{\theta }_{a,v,d}^{I,wane}+{\lambda }_{a}^{v}{U}_{a}^{v,d}-(\sigma +\gamma ){I}_{a}^{v,d}+\frac{1}{D}{I}_{a}^{v,1}\,{\text{, if}}\,d=0\quad \\ {\theta }_{a,v,d}^{I,wane}+{\lambda }_{a}^{v}{U}_{a}^{v,d}-(\sigma +\gamma ){I}_{a}^{v,d}-\frac{1}{D}{I}_{a}^{v,d}\,{\text{, if}}\,d=1\quad \end{array}\right.$$

3 $$\frac{d{A}_{a}^{v,d}}{dt}=\left\{\begin{array}{l}{\theta }_{a,v,d}^{A,wane}+(1-\psi )\sigma {I}_{a}^{v,d}-(\nu +{\eta }_{a}+\gamma ){A}_{a}^{v,d}+\frac{1}{D}{A}_{a}^{v,1}\,{\text{, if}}\,d=0\quad \\ {\theta }_{a,v,d}^{A,wane}+(1-\psi )\sigma {I}_{a}^{v,d}-(\nu +{\eta }_{a}+\gamma ){A}_{a}^{v,d}-\frac{1}{D}{A}_{a}^{v,d}\,{\text{, if}}\,d=1\quad \end{array}\right.$$

4 $$\frac{d{S}_{a}^{v,d}}{dt}=\left\{\begin{array}{l}{\theta }_{a,v,d}^{S,wane}+\psi \sigma {I}_{a}^{v,d}-(\mu +\gamma ){S}_{a}^{v,d}+\frac{1}{D}{S}_{a}^{v,1}\,{\text{, if}}\,d=0\quad \\ {\theta }_{a,v,d}^{S,wane}+\psi \sigma {I}_{a}^{v,d}-(\mu +\gamma ){S}_{a}^{v,d}-\frac{1}{D}{S}_{a}^{v,d}\,{\text{, if}}\,d=1\quad \end{array}\right.$$

5 $$\frac{d{T}_{a}^{v,d}}{dt}=\left\{\begin{array}{l}{\theta }_{a,v,d}^{T,wane}+{\eta }_{a}{A}_{a}^{v,d}+\mu {S}_{a}^{v,d}-(\rho +\gamma ){T}_{a}^{v,d}+\frac{1}{D}{T}_{a}^{v,1}\,{\text{, if}}\,d=0\quad \\ {\theta }_{a,v,d}^{T,wane}+{\eta }_{a}{A}_{a}^{v,d}+\mu {S}_{a}^{v,d}-(\rho +\gamma ){T}_{a}^{v,d}-\frac{1}{D}{T}_{a}^{v,d}\,{\text{, if}}\,d=1\quad \end{array}\right.$$

6 $${\alpha }_{v}=\left\{\begin{array}{ll}(1-h)\alpha \quad &\,{\text{if}}\,v=X\\ 0\quad &\,{\text{if}}\,v\in \{P,{V}_{i},{V}_{e},W,{R}_{i},{R}_{e}\}\\ h\alpha \quad &\,{\text{if}}\,v=H\end{array}\right.$$

7 $${\beta }_{v}=\left\{\begin{array}{ll}\beta \quad &\,{\text{if}}\,v\in \{X,W,H\}\\ (1-{e}_{p})\beta \quad &\,{\text{if}}\,v=P\\ (1-{e}_{v})\beta \quad &\,{\text{if}}\,v\in \{{V}_{i},{V}_{e},{R}_{i},{R}_{e}\}\end{array}\right.$$

8 $${\theta }_{a,v,d}^{Z,wane}=\left\{\begin{array}{ll}\frac{1}{{D}_{P}}{Z}_{a}^{P,d}\quad &\,{\text{if}}\,v=X\\ -\frac{1}{{D}_{P}}{Z}_{a}^{P,d}\quad &\,{\text{if}}\,v=P\\ -\frac{1}{{E}_{V}}{Z}_{a}^{{V}_{i},d}\quad &\,{\text{if}}\,v={V}_{i}\\ \frac{1}{{E}_{V}}{Z}_{a}^{{V}_{i},d}-\frac{1}{{D}_{V}-{E}_{V}}{Z}_{a}^{{V}_{e},d}\quad &\,{\text{if}}\,v={V}_{e}\\ \frac{1}{{D}_{V}-{E}_{V}}{Z}_{a}^{{V}_{e},d}+\frac{1}{{D}_{R}-{E}_{R}}{Z}_{a}^{{R}_{e},d}\quad &\,{\text{if}}\,v=W\\ -\frac{1}{{E}_{R}}{Z}_{a}^{{R}_{i},d}\quad &\,{\text{if}}\,v={R}_{i}\\ \frac{1}{{E}_{R}}{Z}_{a}^{{R}_{i},d}-\frac{1}{{D}_{R}-{E}_{R}}{Z}_{a}^{{R}_{e},d}\quad &\,{\text{if}}\,v={R}_{e}\\ 0\quad &\,{\text{if}}\,v=H\end{array}\right.$$

9 $${\theta }_{a,v,d}^{VoS}={\eta }_{a}{\delta }_{a,d}^{VoS}\times \left\{\begin{array}{ll}-{r}_{1}{U}_{a}^{X,d}\quad &\,{\text{if}}\,v=X\\ {r}_{1}(1-{r}_{2}){U}_{a}^{X,d}-{r}_{1}{r}_{2}{U}_{a}^{P,d}\quad &\,{\text{if}}\,v=P\\ {r}_{1}{r}_{2}{U}_{a}^{X,d}+{r}_{1}{r}_{2}{U}_{a}^{P,d}\quad &\,{\text{if}}\,v={V}_{i}\\ -{r}_{b}{U}_{a}^{v,d}\quad &\,{\text{if}}\,v\in \{{V}_{e},W,{R}_{e}\}\\ {r}_{b}{U}_{a}^{W,d}+{r}_{b}{U}_{a}^{{V}_{e},d}+{r}_{b}{U}_{a}^{{R}_{e},d}\quad &\,{\text{if}}\,v={R}_{i}\\ 0\quad &\,{\text{if}}\,v=H\end{array}\right.$$

10 $${\theta }_{a,v,d}^{VaN}={\delta }^{VaN}\times \left\{\begin{array}{ll}-{r}_{1}{\phi }_{a}^{X,d}\quad &\,{\text{if}}\,v=X\\ {r}_{1}(1-{r}_{2}){\phi }_{a}^{X,d}-{r}_{1}{r}_{2}{\phi }_{a}^{P,d}\quad &\,{\text{if}}\,v=P\\ {r}_{1}{r}_{2}{\phi }_{a}^{X,d}+{r}_{1}{r}_{2}{\phi }_{a}^{P,d}\quad &\,{\text{if}}\,v={V}_{i}\\ -{r}_{b}{\phi }_{a}^{v,d}\quad &\,{\text{if}}\,v\in \{{V}_{e},W,{R}_{e}\}\\ {r}_{b}{\phi }_{a}^{W,d}+{r}_{b}{\phi }_{a}^{{V}_{e},d}+{r}_{b}{\phi }_{a}^{{R}_{e},d}\quad &\,{\text{if}}\,v={R}_{i}\\ 0\quad &\,{\text{if}}\,v=H\end{array}\right.$$

11 $${\theta }_{a,v,d}^{VoD}=\rho {\delta }^{VoD}\times \left\{\begin{array}{ll}-{r}_{1}{T}_{a}^{X,d}\quad &\,{\text{if}}\,v=X\\ {r}_{1}(1-{r}_{2}){T}_{a}^{X,d}-{r}_{1}{r}_{2}{T}_{a}^{P,d}\quad &\,{\text{if}}\,v=P\\ {r}_{1}{r}_{2}{T}_{a}^{X,d}+{r}_{1}{r}_{2}{T}_{a}^{P,d}\quad &\,{\text{if}}\,v={V}_{i}\\ -{r}_{b}{T}_{a}^{v,d}\quad &\,{\text{if}}\,v\in \{{V}_{e},W,{R}_{e}\}\\ {r}_{b}{T}_{a}^{W,d}+{r}_{b}{T}_{a}^{{V}_{e},d}+{r}_{b}{T}_{a}^{{R}_{e},d}\quad &\,{\text{if}}\,v={R}_{i}\\ 0\quad &\,{\text{if}}\,v=H\end{array}\right.$$

Letting ${C}_{a}^{v,d}={I}_{a}^{v,d}+{A}_{a}^{v,d}+{S}_{a}^{v,d}$, ${C}_{a}={\sum }_{v}{\sum }_{d}{C}_{a}^{v,d}$, ${N}_{a}^{v,d}={U}_{a}^{v,d}+{I}_{a}^{v,d}+{A}_{a}^{v,d}+{S}_{a}^{v,d}+{T}_{a}^{v,d}$, ${N}_{a}={\sum }_{v}{\sum }_{d}{N}_{a}^{v,d}$, with *c*_*a*_ denoting the partner change rate and with *ϵ* denoting the level of assortative mixing between activity groups, the force of infection to an individual in activity group *a* and vaccination stratum *v* is given by:

12 $$\begin{array}{lll}{\lambda }_{a}^{v}={c}_{a}{\beta }_{v}\left(\epsilon \frac{{C}_{a}}{{N}_{a}}+(1-\epsilon )\frac{\sum _{a{\prime} }{c}_{a{\prime} }{C}_{a{\prime} }}{\sum _{a{\prime} }{c}_{a{\prime} }{N}_{a{\prime} }}\right)\end{array}$$

### Partner notification

Implementing a strategy which offers vaccination to uninfected notified partners within the model requires quantifying the rate that uninfected individuals in activity group *a*, vaccination stratum *v*, and diagnosis history stratum *d* attend SHCs through partner notification. This is derived as follows:

1. The overall rate of diagnosis from activity group *b* is given by $\sum _{v}\sum _{d}\mu {S}_{b}^{v,d}+{\eta }_{b}{A}_{b}^{v,d}$
2. For each individual who is diagnosed, *κ* individuals are notified.
3. Of the notified individuals, a proportion *p* are infected.
4. Of uninfected notified partners, *ω*_*U*_(*b*, *a*) individuals are from activity group *a*, where *ω*_*U*_ is given by:
  13 $${\omega }_{U}=\left[\begin{array}{cc}\epsilon +(1-\epsilon )\frac{{c}_{L}{U}_{L}}{\sum _{a{\prime} }{c}_{a{\prime} }{U}_{a{\prime} }}&(1-\epsilon )\frac{{c}_{H}{U}_{H}}{\sum _{a{\prime} }{c}_{a{\prime} }{U}_{a{\prime} }}\\ (1-\epsilon )\frac{{c}_{L}{U}_{L}}{\sum _{a{\prime} }{c}_{a{\prime} }{U}_{a{\prime} }}&\epsilon +(1-\epsilon )\frac{{c}_{H}{U}_{H}}{\sum _{a{\prime} }{c}_{a{\prime} }{U}_{a{\prime} }}\\ \end{array}\right]$$
5. The proportion of uninfected notified partners in activity group *a* who are from vaccination stratum *v* and with diagnosis history *d* is given by $\frac{{U}_{a}^{v,d}}{{\sum }_{v{\prime} \in {\mathcal{V}}}{\sum }_{d{\prime} \in {\mathcal{D}}}{U}_{a}^{v{\prime} ,d{\prime} }}$.
6. So, the overall rate of uninfected individuals in subgroup (*a*, *v*, *d*) being notified from a contact in activity group *b* is given by
  14 $$\kappa \times (1-p)\times \frac{{U}_{a}^{v,d}}{\sum _{v{\prime} }\sum _{d{\prime} }{U}_{a}^{v{\prime} ,d{\prime} }}\times {\omega }_{U}(b,a)\times \sum _{v{\prime\prime} }\sum _{d{\prime\prime} }\left({{\mu {S}_{b}^{v}}^{\prime\prime} ,d^{\prime\prime} }+{\eta }_{b}{A}_{b}^{v{\prime\prime} ,d{\prime\prime} }\right)$$
7. So, the overall rate of uninfected individuals in subgroup (*a*, *v*, *d*) being notified by someone in any group is given by:
  15 $${\phi }_{a}^{v,d}=\kappa \times (1-p)\times \frac{{U}_{a}^{v,d}}{\sum _{v{\prime} }\sum _{d{\prime} }{U}_{a}^{v{\prime} ,d{\prime} }}\times \sum _{a{\prime\prime} }\sum _{v{\prime\prime} }\sum _{d{\prime\prime} }{\omega }_{U}(a{\prime\prime} ,a)\left(\mu {S}_{a{\prime\prime} }^{v{\prime\prime} ,d{\prime\prime} }+{\eta }_{a{\prime\prime} }{A}_{a{\prime\prime} }^{v{\prime\prime} ,d{\prime\prime} }\right)$$

This approach allows us to capture partner notification within a compartmental model provided that estimates of *κ* and *p* can be obtained.

Note that infected individuals who attend SHCs through partner notification are already implicitly captured within our framework, as vaccination is offered on diagnosis, and the model is calibrated to diagnosis numbers in England (which includes diagnoses through partner notification).

### Calibration and accounting for uncertainty

Epidemiological parameters are estimated via Bayesian calibration to data from the UK’s GUMCAD and GRASP surveillance systems^7,40^. We account for uncertainty in estimated parameters by using 1000 samples from the joint posterior distribution. Full details of the model calibration, including plots of model trajectories in comparison with the observations used for calibration, are provided in previous work^10^.

We introduce two parameters to incorporate partner notification: *κ*, the number of individuals attending SHC via partner notification per index case (0.39 [95% CrI:0.31–0.47])^7^, and *p*, the prevalence of gonorrhea infection among notified partners (38% [95% CrI:31–45%])^41^.

We account for uncertainty in the interpretation of vaccine uptake data^42^ by considering two vaccine-sentiment scenarios. In our main analysis, all MSM have the same probability of accepting vaccination, as in most modeling analyses. Vaccine uptake (the probability of acceptance when offered) is set at the level observed for MSM HPV vaccination in England^42^, i.e., 40.8% (95% CrI:40.6–41.0). In supplementary analysis, we consider an alternative scenario in which a proportion of individuals are vaccine-willing and the remainder are unwilling and never accept vaccination^12^. In this scenario, the proportion of individuals who are willing to be vaccinated is set to equal the probability of acceptance in our main scenario, to obtain equal initial rates of vaccination in the two scenarios^12^. In both scenarios, 61.7% (95% CrI:61.2–62.1) of those who receive a first dose return to obtain a second dose^42^. A model schematic of vaccination-status strata, including a stratum for those unwilling to be vaccinated, is shown in Supplementary Fig. 9.

### Vaccine-targeting strategies

We compare five vaccine-targeting strategies that could be implemented at SHCs, where the vast majority of gonorrhea diagnoses in England are managed^43^: VoD, VaH, VaN, VaH, and VaN combined (VaH + VaN), and VaR (eligibility detailed in Table 1). We start the model at equilibrium before each vaccination scenario is implemented.

Primary vaccination requires two doses, but some vaccinees only receive one dose due to not returning for the second primary dose. Those who receive only one dose get two-thirds the protection of two doses^20^. Revaccination is offered after a period of time since the last vaccination according to the eligibility criteria of the implemented strategy: those who received a two-dose primary vaccination are offered a single booster dose, while those who received only one primary dose are offered another course of primary vaccination.

We evaluate the impact of each strategy by calculating the yearly number of gonorrhea diagnoses averted, compared to a baseline scenario without vaccination. We evaluate the efficiency of each strategy by calculating the cumulative number of gonorrhea diagnoses averted per dose administered.

In scenario analysis, as before^10^ we vary the level of protection from two primary doses (20 and 40%) and the duration of protection (1.5 years after primary then 3 years after booster vaccination, or 4 years after primary and booster vaccination, or 7.5 years after primary and booster vaccination).

### Health-economic analysis

Health-economic analysis takes the perspective of the UK National Health Service (NHS), considering (i) costs of gonorrhea testing, treatment, and vaccination, and (ii) loss of health due to symptoms, measured in quality-adjusted life years (QALYs). Costs and QALYs are calculated as in Whittles et al. 2022^10^; a brief description of the approach is included below, and health-economic parameters are detailed in Table 5. For the convenience of the reader, equations for model outputs required to evaluate impact, efficiency, and cost-effectiveness are detailed in Table 6.

**Table 5:** Health-economic parameters

	Definition	Value	Distribution	Source
*ξ*	Cost per vaccine dose administered	£18 or £85	-	^10,45,46,51^
*w*^*U*^	Cost of initial test	£94.35 (95% CI: 61.06, 134.79)	Gamma (94.35, 0.2 × 94.35)^*^	^52^
*w*^*S*^	Cost of investigating symptoms	£23.19 (95% CI: 15.01, 31.13)	Gamma (23.19, 0.2 × 23.19)^*^	^52^
*w*^*T*^	Cost of treatment	£85.24 (95% CI: 55.16, 121.73)	Gamma (85.24, 0.2 × 85.24)^*^	^52^
*z*^*T*^	Reduction in the cost of treatment at the initial visit	14%	-	^52^
*w*^*ToC*^	Cost of Test-of-Cure (ToC)	£47.29 (95% CI: 30.60, 67.54)	Gamma (47.29, 0.2 × 47.29)^*^	^52^
*p*^*ToC*^	Proportion returning for ToC	57% (95% CI: 54%, 60%)	Beta (552, 419)	^31,40^
*χ*	Quality-of-Life disutility of symptoms	0.160 (95% CI: 0.136, 0.182)	Main: Pert (0.128, 0.16, 0.192) Supp Tables 3, 4: Pert (0.039, 0.10, 0.16)	^18,47,53^
*w*^*Q*^	QALY value	£20,000 or £30,000	-	^44^
*r*_*disc*_	Annual discount rate	3.5%	-	^44^
*t*_*hor*_	Time horizon	10 or 20 years	-	-

^*^Gamma distributions are parameterized in terms of mean and standard deviation.

**Table 6:** Summary of model outputs

Model outputs	Symbol	Expression
Outputs for activity group *a* diagnosis history stratum *d* in year *t*		
Annual # diagnosed cases, vaccination stratum *v*	${Y}_{D}^{a,v,d}(t)$	$\mathop{\int}\nolimits_{t}^{t+1}\rho {T}_{a}^{v,d}(\tau )d\tau$
Annual # asymptomatic diagnoses, vaccination stratum *v*	${Y}_{A}^{a,v,d}(t)$	$\mathop{\int}\nolimits_{t}^{t+1}{\eta }_{a}{A}_{a}^{v,d}(\tau )d\tau$
Annual # symptomatic diagnoses, vaccination stratum *v*	${Y}_{S}^{a,v,d}(t)$	$\mathop{\int}\nolimits_{t}^{t+1}\mu {S}_{a}^{v,d}(\tau )d\tau$
Annual # uninfected individuals screened, vaccination stratum *v*	${Y}_{U}^{a,v,d}(t)$	$\mathop{\int}\nolimits_{t}^{t+1}{\eta }_{a}{U}_{a}^{v,d}(\tau )d\tau$
Annual # partner notifications to uninfected individuals, vaccination stratum *v*	${Y}_{\phi }^{a,v,d}(t)$	$\mathop{\int}\nolimits_{t}^{t+1}{\phi }_{a}^{v,d}(\tau )d\tau$
Annual # vaccine doses administered to individuals in vaccination stratum *v* ∈ {*X*, *P*}	${\,\text{Doses}\,}_{a}^{v,d}(t)$	${r}_{1}(1+{r}_{2})\left({\delta }^{VoD}{Y}_{D}^{a,v,d}(t)+{\delta }_{a,d}^{VoS}{Y}_{U}^{a,v,d}(t)+{\delta }^{VaN}{Y}_{\phi }^{a,v,d}(t)\right)$
Annual # vaccine doses administered to individuals in vaccination stratum *v* ∈ {*V*_*e*_, *W*, *R*_*e*_}	${\,\text{Doses}\,}_{a}^{v,d}(t)$	${r}_{b}\left({\delta }^{VoD}{Y}_{D}^{a,v,d}(t)+{\delta }_{a,d}^{VoS}{Y}_{U}^{a,v,d}(t)+{\delta }^{VaN}{Y}_{\phi }^{a,v,d}(t)\right)$
Overall outputs in year *t*		
Annual # diagnosed cases	*Y*_*D*_(*t*)	$\sum _{a}\sum _{v}\sum _{d}{Y}_{D}^{a,v,d}(t)$
Annual # asymptomatic diagnoses	*Y*_*A*_(*t*)	$\sum _{a}\sum _{v}\sum _{d}{Y}_{A}^{a,v,d}(t)$
Annual # symptomatic diagnoses	*Y*_*S*_(*t*)	$\sum _{a}\sum _{v}\sum _{d}{Y}_{S}^{a,v,d}(t)$
Annual # vaccine doses administered	Doses(*t*)	$\sum _{a}\sum _{d}{\,\text{Doses}}_{a}^{X,d}(t)+{\text{Doses}}_{a}^{P,d}(t)+{\text{Doses}}_{a}^{W,d}(t)+{\text{Doses}}_{a}^{{V}_{e},d}(t)+{\text{Doses}\,}_{a}^{{R}_{e},d}(t)$
Annual (undiscounted) healthcare costs for uninfected individuals	Cost_*U*_(*t*)	*w*^*U*^*Y*_*U*_(*t*)
Annual (undiscounted) healthcare costs for asymptomatically infected individuals	Cost_*A*_(*t*)	$\left({w}^{U}+{w}^{T}+{p}^{ToC}{w}^{ToC}\right){Y}_{A}(t)$
Annual (undiscounted) healthcare costs for asymptomatically infected individuals	Cost_*S*_(*t*)	$\left({w}^{U}+{w}^{S}+(1-{z}^{T}){w}^{T}+{p}^{ToC}{w}^{ToC}\right){Y}_{S}(t)$
Total annual (undiscounted) healthcare costs	Cost(*t*)	Cost_*U*_(*t*) + Cost_*A*_(*t*) + Cost_*S*_(*t*)
Annual (discounted) value of QALY losses	*Q*(*t*)	$\chi \left(\frac{1}{\mu }+\frac{1}{2\rho }\right){w}^{Q}{Y}_{S}(t)\left({(1+{r}_{disc})}^{-(t+0.5)}\right)$
Overall outputs across the time horizon		
Incremental net cost of vaccination^*^	NC	$\mathop{\sum }\nolimits_{t=0}^{{t}_{hor}-1}\left(\,\text{Cost}\,({t}_{0}+t)-\hat{\,\text{Cost}}({t}_{0}+t)+(\xi \times \text{Doses}\,({t}_{0}+t))\right)\left({(1+{r}_{disc})}^{-(t+0.5)}\right)$
Net monetary benefit of vaccination^*^	NMB	$\mathop{\sum }\nolimits_{0}^{{t}_{hor}-1}\left(Q({t}_{0}+t)-\hat{Q}({t}_{0}+t)\right)-\,\text{NC}\,$

*Note terms with ${\hat{a}}$ (e.g., $\hat{Q}({t}_{0}+t)$) denote values obtained given a counterfactual scenario where vaccination is not introduced.

Costs (£2021-22 GBP) and QALYs are discounted at 3.5% per annum^44^. In our main analysis, we consider a vaccination cost of £18/dose administered, which includes a £10 administration fee^10,45^. We also consider a vaccination cost of £85/dose administered—the vaccine’s list price^46^ plus £10 administration fee.

We calculate the net monetary benefit (NMB) of each vaccination strategy, i.e., the monetary value of averting QALY losses minus the net costs of vaccination, compared with no vaccination. QALY loss is the product of the Quality-of-Life disutility of symptomatic infection^47^ and the average duration of symptoms. For vaccination to be considered cost-effective in the UK, there needs to be at least 50% probability that NMB is positive with a QALY valued at £20,000 and at least 90% probability with a QALY valued at £30,000^44^. Therefore, we calculate NMB with a QALY valued at £20,000 (NMB_ £20k_) and at £30,000 (NMB_ £30k_).

We calculate both impact and cost-effectiveness over a 10-year time horizon; in supplementary analysis, we also consider a 20-year time horizon.

## Supplementary information

Supplementary Materials

## Data Availability

All the data used to inform the model were from published studies or from publicly accessible data sources. All relevant references are detailed in Tables 4, 5.

## Code availability

The underlying code may be made available to qualified researchers on reasonable request from the corresponding author. All analyses were undertaken in R (version 4.4.3), using the R package gonovax (version 0.4.23). High-density regions in Figs. 2, 3 were generated using the R package ggdensity (version 1.0.0.9). Figure 4 and Supplementary Fig. 9 were generated using the R package flodia (version 0.0.12). All other figures were generated using ggplot2 (version 3.5.1).
