# Publisher Correction: Modeling gonorrhea vaccination to find optimal targeting strategies that balance impact with cost-effectiveness

**DOI:** 10.1038/s41541-025-01295-7

**Published:** 2025-11-25

**Authors:** Trystan Leng, Lilith K. Whittles, Dariya Nikitin, Peter J. White

**Affiliations:** 1 MRC Centre for Global Infectious Disease Analysis and NIHR Health Protection Research Unit in Modelling and Health Economics, Imperial College London, London, UK; 2 Modelling and Economics Unit, UK Health Security Agency, London, UK

**Keywords:** Public health, Epidemiology, Infectious diseases

Correction to: *npj Vaccines* 10.1038/s41541-025-01159-0, published online 21 June 2025

In the original version of this article, in Table 6 “{*V*_*e*_, *W*, *R*_*e*_}” was mistakenly replaced by “{*V*_*e*_, *W*, *R*_*e*_}s”. The corrected Table 6 is now displayed in the HTML and PDF versions of the article.

