# Supplementary material for: Modeling gonorrhea vaccination to find optimal targeting strategies that balance impact with cost-effectiveness
Source: NPJ Vaccines. 2025 Jun 21;10:128. doi: 10.1038/s41541-025-01159-0 (PMC12182581; doi:10.1038/s41541-025-01159-0)
Supplement: Supplementary file 1 — Supplementary Materials [file 41541_2025_1159_MOESM1_ESM.pdf]

Supplementary material for
Modeling gonorrhea vaccination to find optimal targeting strategies that balance impact with cost-effectiveness

Trystan Leng, Lilith K Whittles, Dariya Nikitin, Peter J White

Contents

1 Supplementary Note 1: Supplementary trends over 10 years **2**
1.1 Annual diagnoses and prevalence (Supplementary Figure 1) 2
1.2 Population protection under different vaccination strategies (Supplementary Figure 2) 2

2 Supplementary Note 2: Supplementary health-economic analyses **4**
2.1 Incremental analysis (Supplementary Tables 1 & 2) 4
2.2 Comparing different costs per dose (Supplementary Figures 3-5) 7
2.3 Comparing willingness-to-pay thresholds of £20,000/QALY vs £30,000/QALY (Supplementary Figure 6 11
2.4 Using an alternative disutility estimate for symptomatic gonorrhea (Supplementary Tables 3 & 4) . 13
2.5 Considering a longer time-horizon of 20 years (Supplementary Figure 7, Supplementary Tables 5 & 6) 16
2.6 Considering vaccines with longer durations of protection (Supplementary Tables 7-11, Supplementary Figure 8) 20

3 Supplementary Note 3: Alternative vaccine-sentiment scenario, with some unwilling to be vaccinated **26**
3.1 Vaccination-status strata (Supplementary Figure 9) 26
3.2 Health-economic analyses (Supplementary Figure 10, Supplementary Tables 12 & 13) 27

Supplementary References **31**

1 Supplementary Note 1: Supplementary trends over 10 years

1.1 Annual diagnoses and prevalence (Supplementary Figure 1)

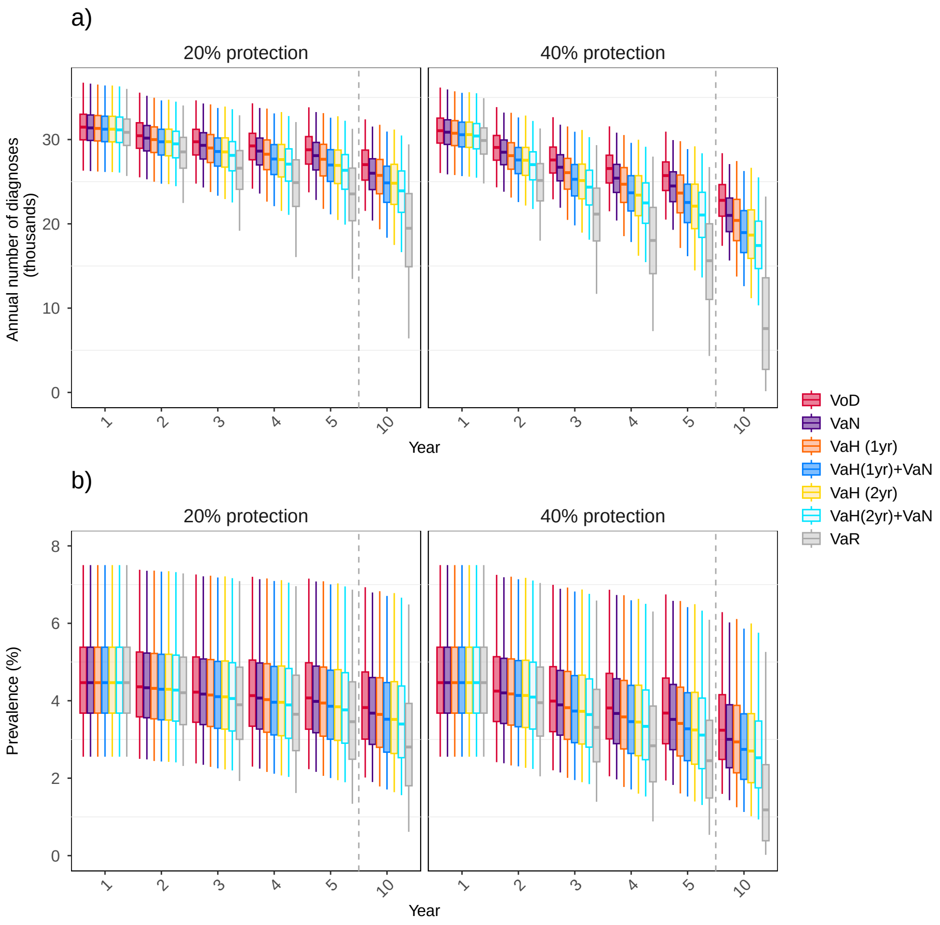

Supplementary Figure 1: Trends in cases and prevalence over 10 years under different strategies. Vaccination strategies considered are Vaccination-on-Diagnosis (VoD); Vaccination-according-to-partner-Notification (VaN); Vaccination-according-to-History (VaH) offering vaccination to those with a diagnosis in the last year [VaH(1yr)] or in the last two years [VaH(2yr)]; VaN combined with VaH(1yr) or VaH(2yr); and Vaccination-according-to-Risk (VaR). The left and right columns show results for vaccines providing 20% and 40% protection after two-dose primary vaccination, respectively. Primary vaccination has a 1.5-year duration of protection, and booster vaccination has a 3-year duration of protection. Section a) annual number of gonorrhea diagnoses. Section b) prevalence of gonorrhea infection at the end of each year. Box plots depict results from 1000 sets of sampled epidemiological and health-economic parameters, with whiskers indicating the 2.5^{th} and 97.5^{th} centiles, boxes the 25th and 75th centiles, and the central line the median (50th centile).

1.2 Population protection under different vaccination strategies (Supplementary Figure 2)

Supplementary Figure 2 shows the proportion of the population protected through vaccination over time. Some individuals have partial protection because they do not receive a 2nd dose whilst others have full protection from two-dose primary vaccination or single-dose revaccination following two-dose primary vaccination. For all strategies, the proportion of the population partially-protected (by one dose) initially increases, but subsequently declines because when revaccination is offered a proportion receives two doses and gains full protection, meaning that subsequent revaccination requires only a single dose to maintain full protection. The proportion of the population with full protection increases for all strategies over the 10 years. For a vaccine with 20% protection, the proportion of the population protected after 10 years for different strategies ranges from 4.3% (95% CrI: 3.4-5.1%) [VoD] to 9.8% (95% CrI: 7.5-11.9%) [VaR]. Because a vaccine with 40% protection averts more cases than a vaccine with 20% protection, fewer individuals are vaccinated upon diagnosis, so accordingly the proportion with vaccine protection is slightly lower.

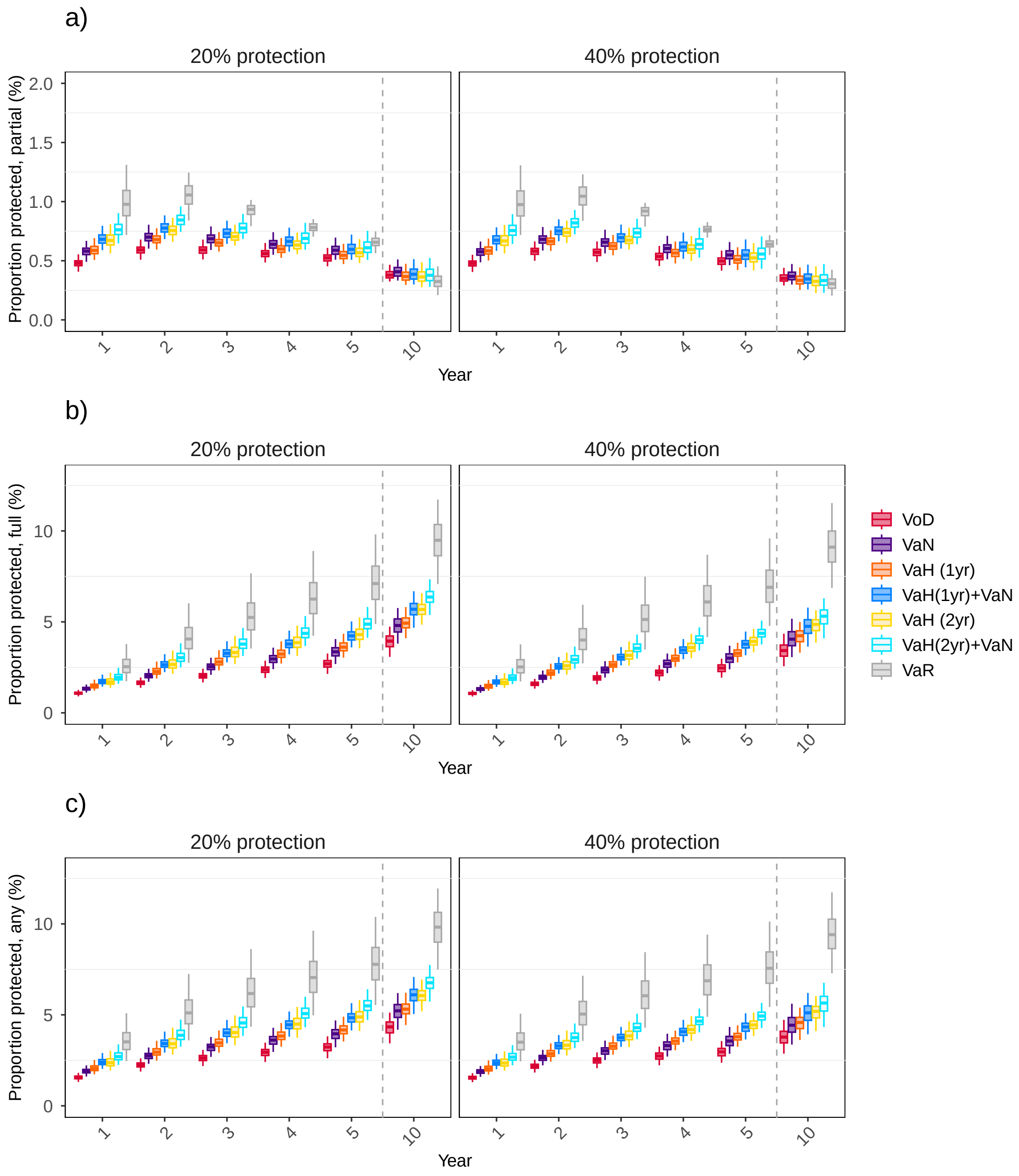

Supplementary Figure 2: Population protection over 10 years under different vaccination strategies. Vaccination strategies considered are Vaccination-on-Diagnosis (VoD); Vaccination-according-to-partner-Notification (VaN); Vaccination-according-to-History (VaH) offering vaccination to those with a diagnosis in the last year [VaH(1yr)] or in the last two years [VaH(2yr)]; VaN combined with VaH(1yr) or VaH(2yr); and Vaccination-according-to-Risk (VaR). The left and right columns show results for vaccines providing 20% and 40% protection after two-dose primary vaccination, respectively. Primary vaccination has a 1.5-year duration of protection, and booster vaccination has a 3-year duration of protection. Section a) the proportion of the population partially protected by one-dose primary vaccination. Section b) the proportion of the population fully protected by two-dose primary vaccination and one-dose booster vaccination. Section c) the proportion of the population with any level of protection. Box plots depict results from 1000 sets of sampled epidemiological and health-economic parameters, with whiskers indicating the 2.5^{th} and 97.5^{th} centiles, boxes the 25th and 75th centiles, and the central line the median (50th centile).

2 Supplementary Note 2: Supplementary health-economic analyses

2.1 Incremental analysis (Supplementary Tables 1 & 2)

In the main paper we present the net monetary benefit of each strategy, compared to a baseline scenario without vaccination. Here, we present the incremental monetary benefit (IMB) of each strategy considered, compared with each lower-impact strategy.

Notably, at £18/dose, VaR and VaH(2yr)+VaN meet both cost-effectiveness thresholds when compared to any of the lower-impact strategies considered, i.e. both have over 50% probability having a positive incremental monetary benefit with a QALY valued at £20,000 and over 90% probability of having a positive incremental monetary benefit with a QALY valued at £30,000.

In general, higher-impact strategies considered are cost-effective (at both thresholds) at £18/dose, when compared to lower-impact strategies. The exceptions to this are VaH(1yr) compared to VaN, and VaH(2yr) compared to VaH(1yr)+VaN.

Supplementary Table 1: Incremental health-economic analysis of vaccination of MSM in England over 10 years, under different targeting strategies, with a vaccine providing 20% protection after two doses, a 1.5-year duration of protection after primary vaccination, and a 3-year duration of protection after booster vaccination. Results are mean values and 95% credible intervals (CrIs) of simulations comparing each vaccination strategy (first column) against lower impact strategies (second column), using 1000 sets of sampled epidemiological and health-economic parameters.

Strategy	Comparison	£18/dose				£85/dose			
		$IMB_{£20k}$	$IMB_{£30k}$	$P(IMB_{£20k}) > 0$	$P(IMB_{£30k}) > 0$	$IMB_{£20k}$	$IMB_{£30k}$	$P(IMB_{£20k}) > 0$	$P(IMB_{£30k}) > 0$
VoD	vs No vaccination	1.8 (-0.6, 5)	2.1 (-0.5, 5.4)	90.6%	93.1%	-9 (-12.4, -5.5)	-8.7 (-12.1, -5)	0%	0%
VaN	vs No vaccination	2.3 (-0.7, 6)	2.6 (-0.5, 6.5)	91.7%	93.9%	-10.5 (-14.5, -6.3)	-10.2 (-14.3, -5.7)	0%	0%
	vs VoD	0.5 (-0.1, 1.1)	0.5 (0, 1.2)	95.6%	97.1%	-1.6 (-2.5, -0.7)	-1.5 (-2.5, -0.6)	0.1%	0.2%
VaH(1yr)	vs No vaccination	2.6 (-0.7, 7.3)	3 (-0.5, 8.1)	91.3%	93.5%	-10.8 (-14.4, -6.8)	-10.5 (-14.1, -6.1)	0%	0%
	vs VoD	0.8 (-0.1, 2.5)	0.9 (0, 2.7)	93.9%	95.6%	-1.9 (-2.4, -1.2)	-1.8 (-2.3, -1)	0.2%	0.2%
	vs VaN	0.3 (-0.1, 1.5)	0.4 (-0.1, 1.6)	80.4%	79.7%	-0.3 (-0.9, 0.7)	-0.3 (-0.9, 0.7)	20%	19.7%
VaH(1yr)+VaN	vs No vaccination	3 (-0.8, 8.3)	3.5 (-0.5, 9.2)	92.3%	94.3%	-12.2 (-16.6, -7.3)	-11.8 (-16.4, -6.5)	0%	0.1%
	vs VoD	1.2 (-0.1, 3.4)	1.4 (0, 3.7)	95.2%	96.5%	-3.3 (-4.4, -1.8)	-3.1 (-4.3, -1.5)	0.2%	0.2%
	vs VaN	0.8 (-0.1, 2.3)	0.9 (0, 2.5)	94.5%	96%	-1.7 (-2.2, -1)	-1.6 (-2.1, -0.8)	0.2%	0.2%
	vs VaH(1yr)	0.4 (0, 1)	0.5 (0, 1)	96.2%	97.4%	-1.4 (-2.3, -0.5)	-1.3 (-2.2, -0.4)	0.2%	0.3%
VaH(2yr)	vs No vaccination	3.2 (-0.8, 9)	3.6 (-0.6, 9.8)	91.6%	93.8%	-12 (-15.8, -7.4)	-11.5 (-15.5, -6.5)	0%	0.2%
	vs VoD	1.4 (-0.1, 4.2)	1.5 (-0.1, 4.6)	94.2%	95.6%	-3.1 (-4.1, -1.8)	-2.9 (-3.9, -1.4)	0.2%	0.2%
	vs VaN	0.9 (-0.1, 3.2)	1 (-0.1, 3.5)	91.2%	92.5%	-1.5 (-2.3, -0.3)	-1.4 (-2.2, -0.3)	1%	1.2%
	vs VaH(1yr)	0.6 (0, 1.7)	0.6 (0, 1.9)	94.4%	95.5%	-1.2 (-1.7, -0.5)	-1.1 (-1.6, -0.4)	0.2%	0.5%
	vs VaH(1yr)+VaN	0.2 (-0.1, 0.9)	0.2 (-0.2, 1)	62%	60%	0.2 (-0.3, 1.1)	0.2 (-0.2, 1.1)	70%	72.7%
VaH(2yr)+VaN	vs No vaccination	3.6 (-0.8, 9.9)	4.1 (-0.5, 10.9)	92.4%	94.5%	-13.3 (-17.8, -7.8)	-12.8 (-17.5, -6.9)	0%	0.2%
	vs VoD	1.8 (-0.1, 5.1)	2 (-0.1, 5.5)	95.1%	96.4%	-4.3 (-5.9, -2.3)	-4.1 (-5.7, -1.8)	0.2%	0.2%
	vs VaN	1.3 (-0.1, 3.9)	1.5 (-0.1, 4.3)	94.6%	95.8%	-2.7 (-3.7, -1.5)	-2.6 (-3.6, -1.1)	0.2%	0.2%
	vs VaH(1yr)	1 (-0.1, 2.5)	1.1 (0, 2.8)	95.4%	96.8%	-2.4 (-3.6, -1)	-2.3 (-3.6, -0.8)	0.2%	0.3%
	vs VaH(1yr)+VaN	0.5 (0, 1.6)	0.6 (0, 1.8)	94.5%	95.6%	-1.1 (-1.6, -0.4)	-1 (-1.5, -0.3)	0.2%	0.6%
	vs VaH(2yr)	0.4 (0, 0.8)	0.5 (0, 0.9)	96.3%	97.5%	-1.3 (-2.2, -0.5)	-1.2 (-2.2, -0.4)	0.1%	0.3%
VaR	vs No vaccination	6.5 (-0.4, 17.4)	7.3 (-0.2, 19.1)	96%	97.1%	-15.6 (-18.7, -9.3)	-14.8 (-18.3, -7.6)	0%	0.1%
	vs VoD	4.7 (0.2, 12.4)	5.2 (0.3, 13.4)	99.1%	99.8%	-6.6 (-8.9, -3.6)	-6.1 (-8.3, -2.6)	0.1%	0.2%
	vs VaN	4.2 (0.2, 11.3)	4.6 (0.3, 12.3)	99.7%	100%	-5 (-7.5, -2.1)	-4.6 (-6.9, -1.7)	0.2%	0.4%
	vs VaH(1yr)	3.9 (0.3, 9.9)	4.3 (0.3, 10.8)	99.6%	100%	-4.7 (-6.8, -2.2)	-4.3 (-6.3, -1.4)	0.2%	0.5%
	vs VaH(1yr)+VaN	3.5 (0.2, 9.1)	3.8 (0.3, 10)	100%	100%	-3.3 (-5.7, -0.9)	-3 (-5.2, -0.6)	0.6%	1.1%
	vs VaH(2yr)	3.3 (0.3, 8.4)	3.6 (0.3, 9)	100%	100%	-3.6 (-5.6, -1.4)	-3.2 (-5.2, -0.9)	0.3%	0.9%
	vs VaH(2yr)+VaN	2.9 (0.3, 7.5)	3.2 (0.3, 8.3)	100%	100%	-2.3 (-4.7, 0.2)	-2 (-4.3, 0.3)	3.3%	5.1%

Abbreviations: $IMB_{£20k}$, ($IMB_{£30k}$) incremental monetary benefit with a quality-adjusted life year valued at £20,000 (£30,000 shown in blue text) of a strategy (first column) to a comparison strategy (second column); VoD, Vaccination-on-Diagnosis; VaN, Vaccination-according-to-partner-Notification; VaH(1yr), Vaccination-according-to-History (diagnosed in last year); VaH(2yr), Vaccination-according-to-History (diagnosed in last 2 years); VaR, Vaccination-according-to-Risk.

Supplementary Table 2: Incremental health-economic analysis of vaccination of MSM in England over 10 years, under different targeting strategies, with a vaccine providing 40% protection after two doses, a 1.5-year duration of protection after primary vaccination, and a 3-year duration of protection after booster vaccination. Results are mean values and 95% credible intervals (CrIs) of simulations comparing each vaccination strategy (first column) against lower impact strategies (second column), using 1000 sets of sampled epidemiological and health-economic parameters.

Strategy	Comparison	£18/dose				£85/dose			
		$IMB_{£20k}$	$IMB_{£30k}$	$P(IMB_{£20k}) > 0$	$P(IMB_{£30k}) > 0$	$IMB_{£20k}$	$IMB_{£30k}$	$P(IMB_{£20k}) > 0$	$P(IMB_{£30k}) > 0$
VoD	vs No vaccination	6.3 (2.2, 11.6)	6.9 (2.5, 12.6)	100%	100%	-3.6 (-8.7, 2.2)	-3 (-8.3, 3.1)	10.4%	15.3%
VaN	vs No vaccination	7.6 (2.8, 13.8)	8.3 (3.2, 14.5)	100%	100%	-4 (-10, 2.8)	-3.3 (-9.6, 4)	11.7%	16.8%
	vs VoD	1.3 (0.6, 2.2)	1.4 (0.6, 2.3)	100%	100%	-0.4 (-1.4, 0.6)	-0.3 (-1.4, 0.8)	23.9%	31.6%
VaH(1yr)	vs No vaccination	8.5 (2.5, 16.4)	9.2 (2.9, 17.6)	100%	100%	-3.8 (-9.9, 4.2)	-3.1 (-9.5, 5.4)	14.8%	20.2%
	vs VoD	2.1 (0.4, 4.9)	2.3 (0.4, 5.3)	100%	100%	-0.2 (-1.3, 1.9)	0 (-1.3, 2.4)	32.8%	39.4%
	vs VaN	0.8 (-0.3, 3.1)	0.9 (-0.3, 3.3)	85.3%	85.2%	0.2 (-0.3, 1.3)	0.2 (-0.2, 1.6)	57.2%	62.8%
VaH(1yr)+VaN	vs No vaccination	9.6 (3.1, 17.9)	10.4 (3.5, 19.1)	100%	100%	-4.1 (-11.1, 4.7)	-3.3 (-10.6, 6.1)	15.6%	21.5%
	vs VoD	3.3 (0.9, 6.5)	3.5 (1.1, 6.9)	100%	100%	-0.5 (-2.5, 2.5)	-0.2 (-2.4, 3.1)	31.3%	40.1%
	vs VaN	2 (0.4, 4.4)	2.1 (0.4, 4.7)	100%	100%	-0.1 (-1.2, 1.8)	0 (-1.1, 2.2)	35.7%	42.9%
	vs VaH(1yr)	1.1 (0.5, 1.8)	1.2 (0.6, 1.9)	100%	100%	-0.3 (-1.3, 0.6)	-0.2 (-1.2, 0.7)	28.2%	37.1%
VaH(2yr)	vs No vaccination	10 (2.8, 19.5)	10.9 (3.1, 21.1)	100%	100%	-3.8 (-10.8, 5.8)	-2.9 (-10.2, 7.2)	18.3%	24.6%
	vs VoD	3.7 (0.6, 8.2)	4 (0.7, 9)	100%	100%	-0.2 (-2.3, 3.6)	0.1 (-2.2, 4.3)	36.6%	43.1%
	vs VaN	2.4 (0, 6.4)	2.6 (0.1, 6.9)	98.2%	98.4%	0.2 (-1.1, 2.9)	0.4 (-1, 3.4)	43%	49.9%
	vs VaH(1yr)	1.5 (0.3, 3.4)	1.7 (0.3, 3.6)	100%	100%	0 (-1, 1.6)	0.1 (-1, 1.8)	42.6%	50.3%
	vs VaH(1yr)+VaN	0.4 (-0.4, 2)	0.4 (-0.5, 2.1)	69.3%	69.3%	0.3 (0, 1.1)	0.3 (0, 1.3)	95.1%	96%
VaH(2yr)+VaN	vs No vaccination	11 (3.3, 20.8)	12 (3.7, 22.4)	100%	100%	-4.1 (-11.8, 6.3)	-3.1 (-11.3, 7.9)	18.6%	25.9%
	vs VoD	4.7 (1.1, 9.5)	5.1 (1.3, 10.3)	100%	100%	-0.5 (-3.4, 4)	-0.1 (-3.2, 4.7)	35.7%	42.7%
	vs VaN	3.4 (0.6, 7.5)	3.7 (0.7, 8.1)	100%	100%	-0.1 (-2.1, 3.2)	0.2 (-2, 3.9)	40.1%	47.4%
	vs VaH(1yr)	2.6 (0.8, 4.7)	2.8 (0.9, 5)	100%	100%	-0.3 (-2.1, 2.1)	-0.1 (-2, 2.4)	39.4%	45.7%
	vs VaH(1yr)+VaN	1.4 (0.2, 3.1)	1.5 (0.3, 3.3)	100%	100%	0 (-0.9, 1.5)	0.1 (-0.9, 1.7)	45.1%	52%
	vs VaH(2yr)	1 (0.5, 1.6)	1.1 (0.6, 1.7)	100%	100%	-0.3 (-1.2, 0.5)	-0.2 (-1.1, 0.6)	29%	38.3%
VaR	vs No vaccination	17.5 (4.7, 32.1)	19 (5.3, 34.7)	100%	100%	-3.9 (-12.1, 7)	-2.4 (-11.5, 9.2)	21.4%	30.9%
	vs VoD	11.2 (2.5, 20.7)	12.1 (2.7, 22.4)	100%	100%	-0.3 (-4.2, 5.5)	0.6 (-3.6, 7.1)	42.2%	54%
	vs VaN	9.9 (1.8, 18.8)	10.7 (2, 20.1)	100%	100%	0.1 (-3.5, 5.3)	0.9 (-2.9, 6.5)	46.3%	60.2%
	vs VaH(1yr)	9 (2.1, 16.4)	9.8 (2.4, 17.5)	100%	100%	-0.1 (-4.1, 4.6)	0.7 (-3.1, 5.6)	45%	58.9%
	vs VaH(1yr)+VaN	7.9 (1.5, 14.8)	8.5 (1.6, 15.8)	100%	100%	0.2 (-3.9, 4.5)	0.9 (-3.1, 5.3)	53.3%	66.7%
	vs VaH(2yr)	7.5 (1.8, 13.3)	8.1 (2, 14.3)	100%	100%	-0.1 (-4.8, 4)	0.5 (-4, 4.8)	47.9%	61.9%
	vs VaH(2yr)+VaN	6.5 (1.2, 12)	7 (1.3, 12.9)	100%	100%	0.2 (-4.9, 3.9)	0.7 (-4.1, 4.5)	58.1%	68.7%

Abbreviations: $IMB_{£20k}$, ($IMB_{£30k}$) incremental monetary benefit with a quality-adjusted life year valued at £20,000 (£30,000 shown in blue text) of a strategy (first column) to a comparison strategy (second column); VoD, Vaccination-on-Diagnosis; VaN, Vaccination-according-to-partner-Notification; VaH(1yr), Vaccination-according-to-History (diagnosed in last year); VaH(2yr), Vaccination-according-to-History (diagnosed in last 2 years); VaR, Vaccination-according-to-Risk.

2.2 Comparing different costs per dose (Supplementary Figures 3-5)

Figure 3a,b in the main paper considers vaccination costing £18/dose administered, and Figure 3c considers the probability that each strategy has a positive net monetary benefit as cost per dose is varied, compared to a strategy of no vaccination.

Here, we present additional results considering the relationship between costs per dose and cost-effectiveness. In Supplementary Figure 3a we present how the net monetary benefit of each strategy (compared to no vaccination), varies with cost per dose. We observe that $\text{NMB}_{£30k}$ decreases linearly with cost per dose. In Supplementary Figure 3b we present the expected loss associated with each strategy, i.e. the mean difference in NMB between the chosen strategy and the strategy with the highest NMB. In Supplementary Figure 4, we present the plots of the probability each strategy has the highest net monetary benefit (compared to no vaccination) of all strategies considered. If VaR is included (Supplementary Figure 4a), then either VaR has the highest probability of returning the highest NMB (at low costs per dose administered), or no vaccination does (at high costs per dose administered). If VaR is excluded (Supplementary Figure 4b) then either VaH(2yr)+VaN has the highest probability of returning the highest NMB, or a strategy of no vaccination does so.

In Supplementary Figure 5 we present results with vaccination costing £85/dose administered, corresponding to the UK list price of £75[1] plus £10 administration cost[2]. In our main analysis (£18/dose administered), higher-impact strategies have lower net costs (as savings in treatment considerably outweigh vaccination costs), but this no longer holds true at £85/dose. For a vaccine with 20% protection, net costs increase approximately linearly with QALYs gained by a strategy; consequently, VoD has the highest $\text{NMB}_{£30k}$ (-£8.7M [95% CrI: -£12.1M to -£5.0M]). At 40% protection, net costs remain fairly constant between strategies; as in our main analysis, VaR has the highest $\text{NMB}_{£30k}$ (-£2.4M [95% CrI: -£11.5M to £9.2M]).

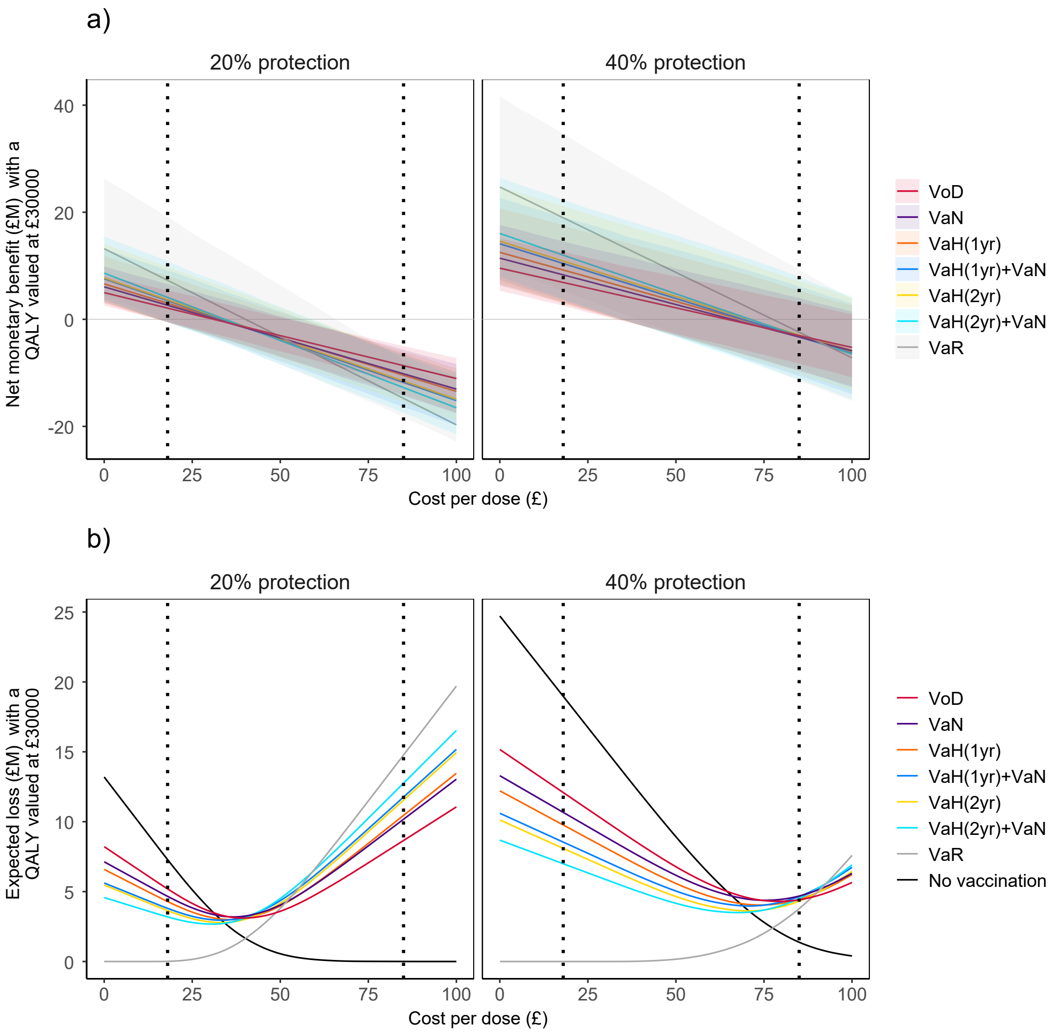

Supplementary Figure 3: Additional cost-effectiveness plots over the first 10 years of a vaccination program under different targeting strategies, varying cost per dose administered. Vaccination strategies considered are Vaccination-on-Diagnosis (VoD); Vaccination-according-to-partner-Notification (VaN); Vaccination- according-to-History (VaH) offering vaccination to those with a diagnosis in the last year [VaH(1yr)] or in the last two years [VaH(2yr)]; VaN combined with VaH(1yr) or VaH(2yr); Vaccination-according-to-Risk (VaR); and no vaccination. The left and right columns show results for vaccines providing 20% and 40% protection after two-dose primary vaccination, respectively. Primary vaccination has a 1.5-year duration of protection, and booster vaccination has a 3-year duration of protection. In all sections we consider a QALY valued at £30,000 and we vary costs per dose administered from £0 to £100. Section a) net monetary benefit (NMB), compared to no vaccination – shaded regions represent 95% credible intervals. Section b) expected loss, i.e. the expected difference in NMB between a strategy and the most cost-effective strategy. All plots use 1000 sets of sampled epidemiological and health-economic parameters.

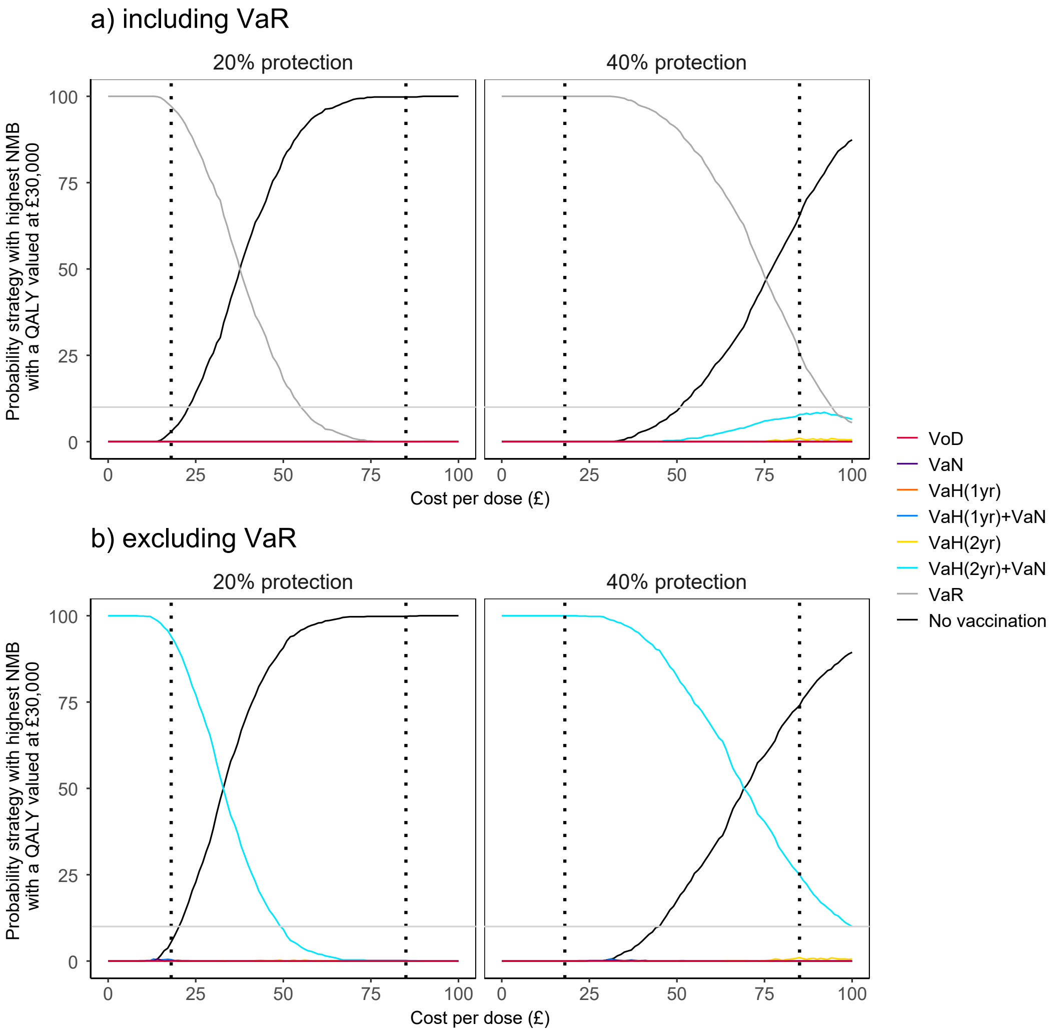

Supplementary Figure 4: Probability of highest net monetary benefit, varying cost per dose administered. Vaccination strategies considered are Vaccination-on-Diagnosis (VoD); Vaccination-according-to-partner-Notification (VaN); Vaccination-according-to-History (VaH) offering vaccination to those with a diagnosis in the last year [VaH(1yr)] or in the last two years [VaH(2yr)]; VaN combined with VaH(1yr) or VaH(2yr); Vaccination-according-to-Risk (VaR); and no vaccination. The left and right columns show results for vaccines providing 20% and 40% protection after two-dose primary vaccination, respectively. Primary vaccination has a 1.5-year duration of protection, and booster vaccination has a 3-year duration of protection. Section a) probability that a vaccination strategy has the highest NMB of all strategies considered, including VaR. Section b) probability that a vaccination strategy has the highest NMB of all strategies considered, excluding VaR. A horizontal line at 10% probability is shown, and the point at which 'No vaccination' crosses this line indicates a threshold for cost-effectiveness: at costs per dose lower than this, at least one vaccination strategy has at least 90% probability of having a positive net monetary benefit, whereas at higher costs per dose no strategy has 90% probability of having a positive net monetary benefit. All plots use 1000 sets of sampled epidemiological and health-economic parameters.

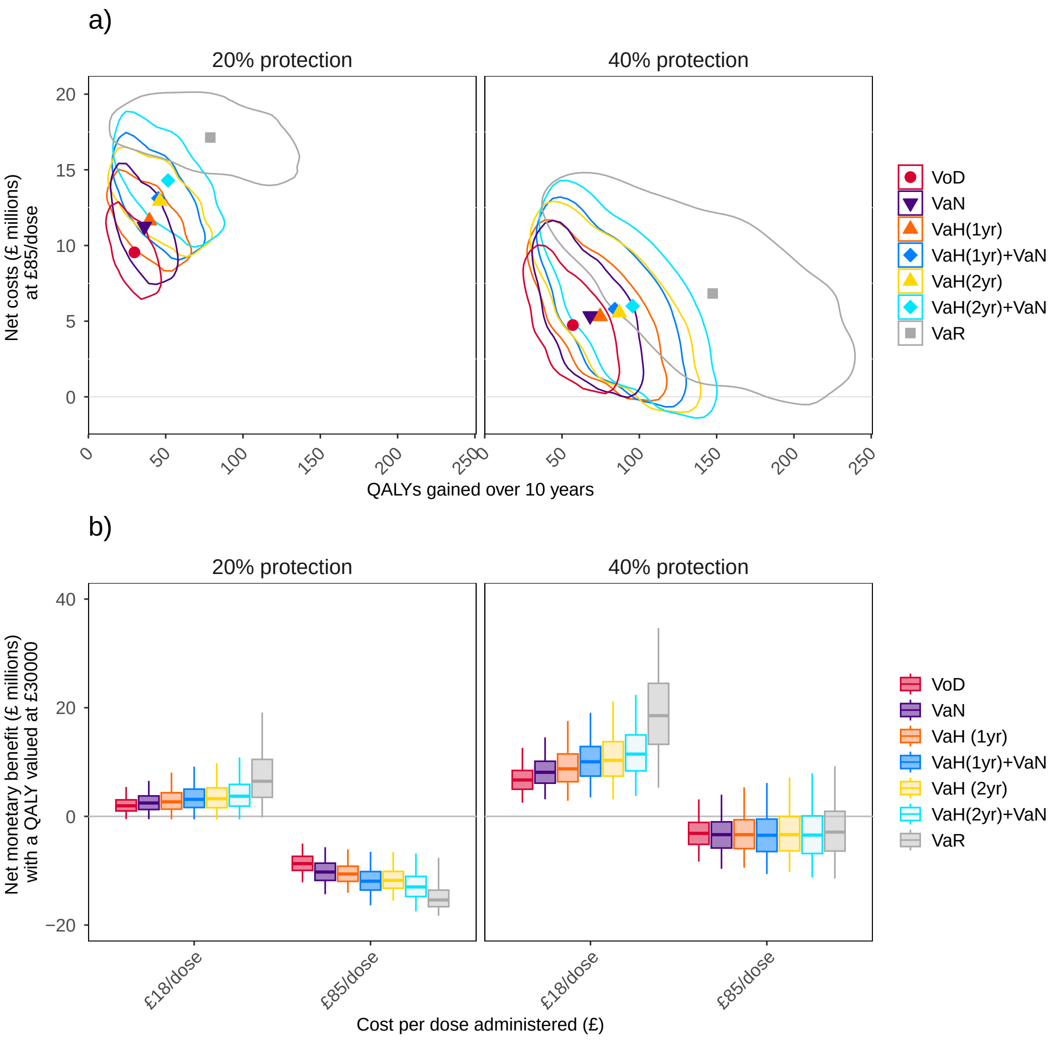

Supplementary Figure 5: Cost-effectiveness of vaccination: comparing results at £18/dose and £85/dose administered. Vaccination strategies considered are Vaccination-on-Diagnosis (VoD); Vaccination-according-to-partner-Notification (VaN); Vaccination-according-to-History (VaH) offering vaccination to those with a diagnosis in the last year [VaH(1yr)] or in the last two years [VaH(2yr)]; VaN combined with VaH(1yr) or VaH(2yr); and Vaccination-according-to-Risk (VaR). The left and right columns show results for vaccines providing 20% and 40% protection after two-dose primary vaccination, respectively. Primary vaccination has a 1.5-year duration of protection, and booster vaccination has a 3-year duration of protection. Section a) cost-effectiveness planes, for a vaccine costing £85/dose administered. The x-axis shows quality-adjusted life years gained by each strategy over 10 years, while the y-axis shows net costs of each strategy over 10 years (negative costs indicate that savings in testing and treatment outweigh vaccination costs). VoD is shown with a circle marker, VaN is shown with a triangle pointing downwards, VaH(1yr) and VaH(2yr) are shown with a triangle pointing upwards, VaH(1yr)+VaN and VaH(2yr)+VaN are shown with diamond markers, and VaR is shown with a square marker. Point markers show mean values, while outlined regions show high density regions which include 80% of model results[3]. Section b) box plots of the net monetary benefit of each strategy over 10 years, for a vaccine costing £18/dose administered (our main scenario) and £85/dose administered, with a QALY valued at £30,000. Box plot whiskers indicate the 2.5^{th} and 97.5^{th} centiles, boxes the 25th and 75th centiles, and the central line the median (50th centile). All plots use 1000 sets of sampled epidemiological and health-economic parameters.

2.3 Comparing willingness-to-pay thresholds of £20,000/QALY vs £30,000/QALY (Supplementary Figure 6)

Supplementary Figure 6 shows the probability that considered vaccination strategies are cost-effective (compared to no vaccination) at different costs per dose administered when a QALY is valued at £20,000, alongside probabilities when a QALY is valued at £30,000 (which is also presented in the main paper). At 20% protection, NMB$_{£20k} > 0$ with $\geq 50\%$ probability if vaccination costs $\leq$£28/dose administered for VoD, and $\leq$£35/dose administered for VaR, with all other considered strategies lying between these two values. At 40% protection, NMB$_{£20k} > 0$ with $\geq 50\%$ probability if vaccination costs $\leq$£59/dose administered for VoD, and $\leq$£71/dose administered for VaR, with all other considered strategies lying between these two values.

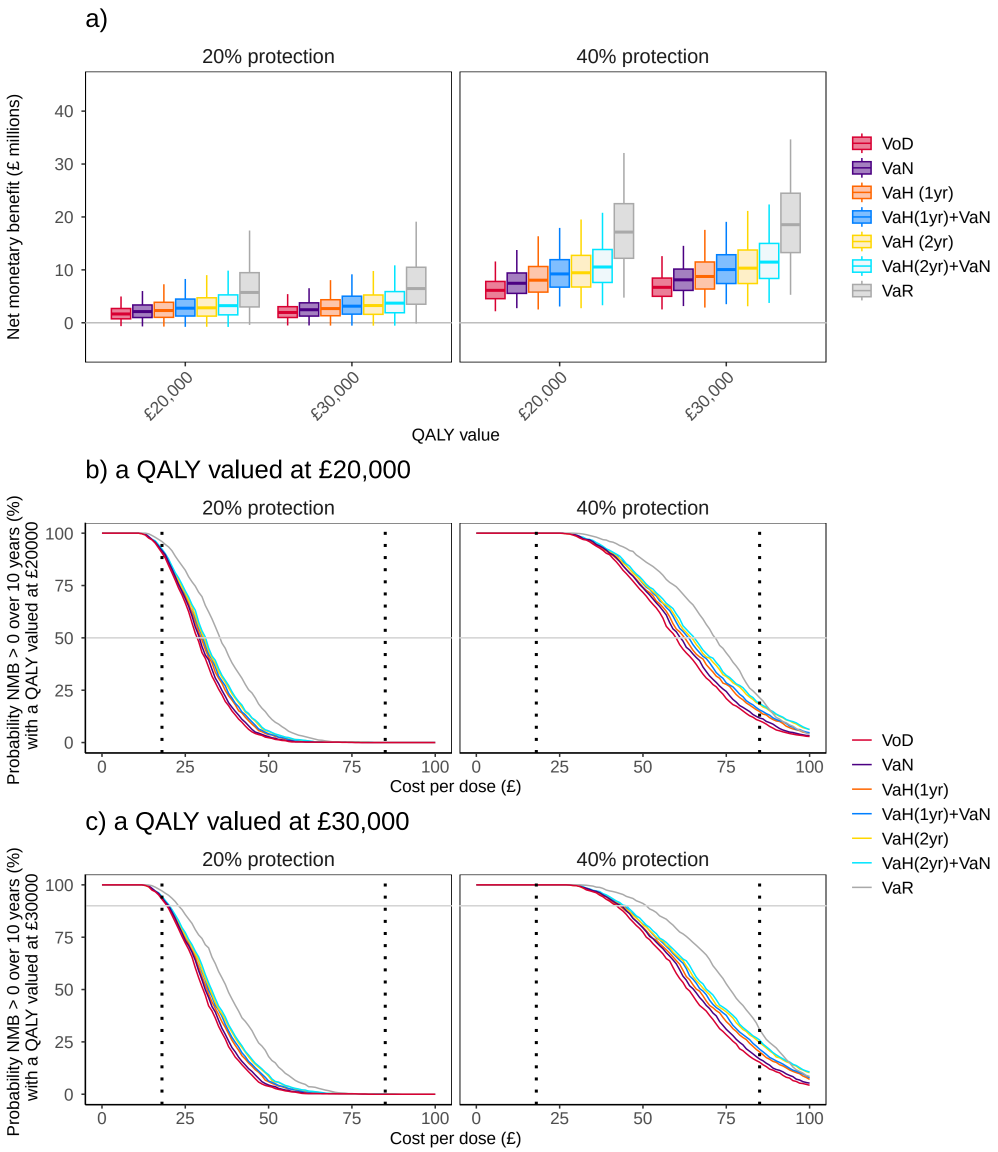

Supplementary Figure 6: Cost-effectiveness of vaccination with a QALY valued at £20,000 or £30,000. Vaccination strategies considered are Vaccination-on-Diagnosis (VoD); Vaccination-according-to-partner-Notification (VaN); Vaccination-according-to-History (VaH) offering vaccination to those with a diagnosis in the last year [VaH(1yr)] or in the last two years [VaH(2yr)]; VaN combined with VaH(1yr) or VaH(2yr); and Vaccination-according-to-Risk (VaR). The left and right columns show results for vaccines providing 20% and 40% protection after two-dose primary vaccination, respectively. Primary vaccination has a 1.5-year duration of protection, and booster vaccination has a 3-year duration of protection. Section a) box plots of the net monetary benefit of each targeting strategy over 10 years, for a vaccine costing £18/dose administered, with a QALY valued at £20,000 or £30,000 (presented in the main paper). Box plot whiskers indicate the 2.5^{th} and 97.5^{th} centiles, boxes the 25th and 75th centiles, and the central line the median (50th centile). Sections b,c) probability that vaccination is cost-effective (i.e. net monetary benefit is positive) at different costs per dose administered, with a QALY valued at b) £20,000 or c) £30,000 (presented in the main paper). All plots use 1000 sets of sampled epidemiological and health-economic parameters.

2.4 Using an alternative disutility estimate for symptomatic gonorrhea (Supplementary Tables 3 & 4)

Supplementary Tables 3 & 4 show the QALYs gained, net monetary benefit, and the probability that vaccination is cost-effective using two different estimates of the disutility of symptomatic gonorrhea. The first estimate, used in the main paper and our previous work[4,5], is based on the disutility of symptomatic gonorrhea from an Institute of Medicine (IoM) study[6]. The second estimate is derived using the method of Li et al.[7], which takes the point estimate of IoM as its upper bound, but derives a lower bound by applying a scaling factor to the point estimate of IoM based on the relative disutility of urethritis to epididymitis reported in the 2019 Global Burden of Disease (GBD) study[8], and the central value is taken as the average of these two values, i.e. we sample the disutility of symptomatic gonorrhea from Pert(0.039, 0.10, 0.16). Using this alternative disutility estimate reduces the QALYs gained by vaccination strategies by approximately 37%, but does not qualitatively impact results.

Supplementary Table 3: Health-economic analysis of vaccination of MSM in England over 10 years, under different pragmatic vaccination strategies aiming to vaccinate high-risk MSM, for a vaccine with 20% protection after two doses, a 1.5-year duration of protection after primary vaccination, and a 3-year duration of protection after booster vaccination, using an alternative disutility of gonorrhea symptoms estimate. Results are mean values and 95% credible intervals (CrIs) of simulations comparing each vaccination strategy against no vaccination, using 1000 sets of sampled epidemiological and health-economic parameters. This alternative disutility distribution [Pert(0.039, 0.1, 0.16)] is based on the method described by Li et al 2023.

Strategy	QALYs gained	£18/dose		£85/dose	
		$NMB_{£20k}$ (£M)<br><br>$NMB_{£30k}$ (£M)	Prob $NMB_{£20k}$>0<br><br>Prob $NMB_{£30k}$>0	$NMB_{£20k}$ (£M)<br><br>$NMB_{£30k}$ (£M)	Prob $NMB_{£20k}$>0<br><br>Prob $NMB_{£30k}$>0
VoD	18.6<br>(7.7, 36.8)	1.6<br>(-0.8, 4.7)<br>1.8<br>(-0.7, 4.9)	88.6%<br><br>90.5%	-9.2<br>(-12.5, -5.8)<br>-9<br>(-12.4, -5.6)	0%<br><br>0%
VaN	22.7<br>(9.5, 44.8)	2<br>(-0.9, 5.7)<br>2.2<br>(-0.7, 5.9)	89.9%<br><br>91.3%	-10.8<br>(-14.8, -6.7)<br>-10.6<br>(-14.6, -6.3)	0%<br><br>0%
VaH(1yr)	24.7<br>(9.4, 53)	2.3<br>(-0.9, 7)<br>2.6<br>(-0.8, 7.4)	89.5%<br><br>90.8%	-11.1<br>(-14.6, -7.2)<br>-10.9<br>(-14.4, -6.8)	0%<br><br>0%
VaH(1yr)+ VaN	28.3<br>(11, 59.6)	2.7<br>(-0.9, 7.8)<br>3<br>(-0.8, 8.2)	90.3%<br><br>91.9%	-12.6<br>(-16.8, -7.9)<br>-12.3<br>(-16.7, -7.5)	0%<br><br>0%
VaH(2yr)	28.9<br>(10.4, 62.9)	2.8<br>(-0.9, 8.4)<br>3.1<br>(-0.8, 8.9)	90.0%<br><br>91.3%	-12.3<br>(-16, -8)<br>-12.1<br>(-15.9, -7.4)	0%<br><br>0%
VaH(2yr)+ VaN	32.2<br>(12, 69.2)	3.2<br>(-1, 9.3)<br>3.5<br>(-0.9, 9.9)	90.5%<br><br>92.1%	-13.7<br>(-18.1, -8.5)<br>-13.3<br>(-17.9, -7.9)	0%<br><br>0%
VaR	49.3<br>(14.6, 110.4)	5.9<br>(-0.6, 16.3)<br>6.4<br>(-0.4, 17.3)	94.9%<br><br>95.6%	-16.1<br>(-19.2, -10.4)<br>-15.7<br>(-18.9, -9.7)	0%<br><br>0%

Abbreviations: M, millions; $NMB_{£20k}$, net monetary benefit with a QALY valued at £20,000; $NMB_{£30k}$, net monetary benefit with a QALY valued at £30,000 (shown in blue text); QALY, quality-adjusted life year; VoD, Vaccination-on-Diagnosis; VaN, Vaccination-according-to-partner-Notification; VaH(1yr), Vaccination-according-to-History (diagnosed in last year); VaH(2yr), Vaccination-according-to-History (diagnosed in last 2 years); VaR, Vaccination-according-to-Risk.

Supplementary Table 4: Health-economic analysis of vaccination of MSM in England over 10 years, under different pragmatic vaccination strategies aiming to vaccinate high-risk MSM, for a vaccine with 40% protection after two doses, a 1.5-year duration of protection after primary vaccination, and a 3-year duration of protection after booster vaccination, using an alternative disutility of gonorrhea symptoms estimate. Results are mean values and 95% credible intervals (CrIs) of simulations comparing each vaccination strategy against no vaccination, using 1000 sets of sampled epidemiological and health-economic parameters. This alternative disutility distribution [Pert(0.039, 0.1, 0.16)] is based on the method described by Li et al 2023.

Strategy	QALYs gained	£18/dose		£85/dose	
		$NMB_{£20k}$ (£M)<br><br>$NMB_{£30k}$ (£M)	Prob $NMB_{£20k}$>0<br><br>Prob $NMB_{£30k}$>0	$NMB_{£20k}$ (£M)<br><br>$NMB_{£30k}$ (£M)	Prob $NMB_{£20k}$>0<br><br>Prob $NMB_{£30k}$>0
VoD	35.7<br>(15.7, 67.6)	5.9<br>(1.9, 11)<br>6.2<br>(2.1, 11.5)	100%<br><br>100%	-4<br>(-8.9, 1.5)<br>-3.7<br>(-8.7, 2)	7.0%<br><br>9.6%
VaN	42.7<br>(18.9, 79.7)	7.1<br>(2.4, 13)<br>7.5<br>(2.7, 13.6)	100%<br><br>100%	-4.5<br>(-10.4, 2)<br>-4.1<br>(-10.1, 2.6)	8.7%<br><br>11.4%
VaH(1yr)	46.7<br>(19.2, 94)	7.9<br>(2.3, 15.7)<br>8.4<br>(2.6, 16.4)	100%<br><br>100%	-4.4<br>(-10.3, 3.2)<br>-3.9<br>(-10, 4.1)	10.7%<br><br>14.0%
VaH(1yr)+ VaN	52.7<br>(22.3, 104.3)	9<br>(2.8, 17.1)<br>9.5<br>(3.1, 18)	100%<br><br>100%	-4.7<br>(-11.6, 3.5)<br>-4.2<br>(-11.3, 4.6)	11.6%<br><br>14.9%
VaH(2yr)	54.5<br>(21.7, 111)	9.4<br>(2.4, 18.9)<br>9.9<br>(2.8, 19.9)	100%<br><br>100%	-4.5<br>(-11.1, 4.6)<br>-3.9<br>(-10.9, 5.6)	14.1%<br><br>17.8%
VaH(2yr)+ VaN	59.9<br>(24.7, 118.7)	10.3<br>(3, 20.2)<br>10.9<br>(3.3, 21)	100%<br><br>100%	-4.8<br>(-12.4, 4.8)<br>-4.2<br>(-12.2, 6.1)	14.6%<br><br>18.1%
VaR	92.3<br>(32.2, 184.1)	16.4<br>(4.3, 30.7)<br>17.3<br>(4.8, 32.4)	100%<br><br>100%	-5<br>(-12.7, 5.7)<br>-4.1<br>(-12.2, 7)	13.8%<br><br>19.4%

Abbreviations: M, millions; $NMB_{£20k}$, net monetary benefit with a QALY valued at £20,000; $NMB_{£30k}$, net monetary benefit with a QALY valued at £30,000 (shown in blue text); QALY, quality-adjusted life year; VoD, Vaccination-on-Diagnosis; VaN, Vaccination-according-to-partner-Notification; VaH(1yr), Vaccination-according-to-History (diagnosed in last year); VaH(2yr), Vaccination-according-to-History (diagnosed in last 2 years); VaR, Vaccination-according-to-Risk.

2.5 Considering a longer time-horizon of 20 years (Supplementary Figure 7, Supplementary Tables 5 & 6)

Supplementary Figure 7 shows net monetary benefit and the probability that considered vaccination strategies are cost-effective (compared to no vaccination) at different costs per dose administered over 20 years, alongside corresponding results when cost- effectiveness is evaluated over 10 years (which are also presented in the main paper). At 20% protection, $\text{NMB}_{£30k} > 0$ with ≥90% probability if vaccination costs ≤£27/dose administered for VoD, and ≤£32/dose administered for VaR, with all other considered strategies lying between these two values. At 40% protection, $\text{NMB}_{£30k} > 0$ with ≥90% probability if vaccination costs ≤£59/dose administered for VoD, and ≤£73/dose administered for VaR, with all other considered strategies lying between these two values.

Supplementary Tables 5 and 6 show health-economic analysis evaluated over 20 years instead of 10 years. Each strategy is more likely to be cost-effective over 20 years than 10 years, with $\text{NMB}_{£20k}$ and $\text{NMB}_{£30k}$ between 2.3–2.6× across strategies considered (at £18/dose administered). However, at 20% protection (Supplementary Table 5) no strategy is likely to be cost-effective at £85/dose administered – the most likely being VaR, which has 4.1% probability of $\text{NMB}_{£20k} > 0$ and 7.4% probability of $\text{NMB}_{£30k} > 0$. At 40% protection (Supplementary Table 6), each strategy has over 50% probability that $\text{NMB}_{£20k} > 0$ (ranging from 51.8% [VoD] to 76.1% [VaR]), but no strategy has over 90% probability that $\text{NMB}_{£30k} > 0$ (ranging from 59.3% [VoD] to 82.2% [VaR]).

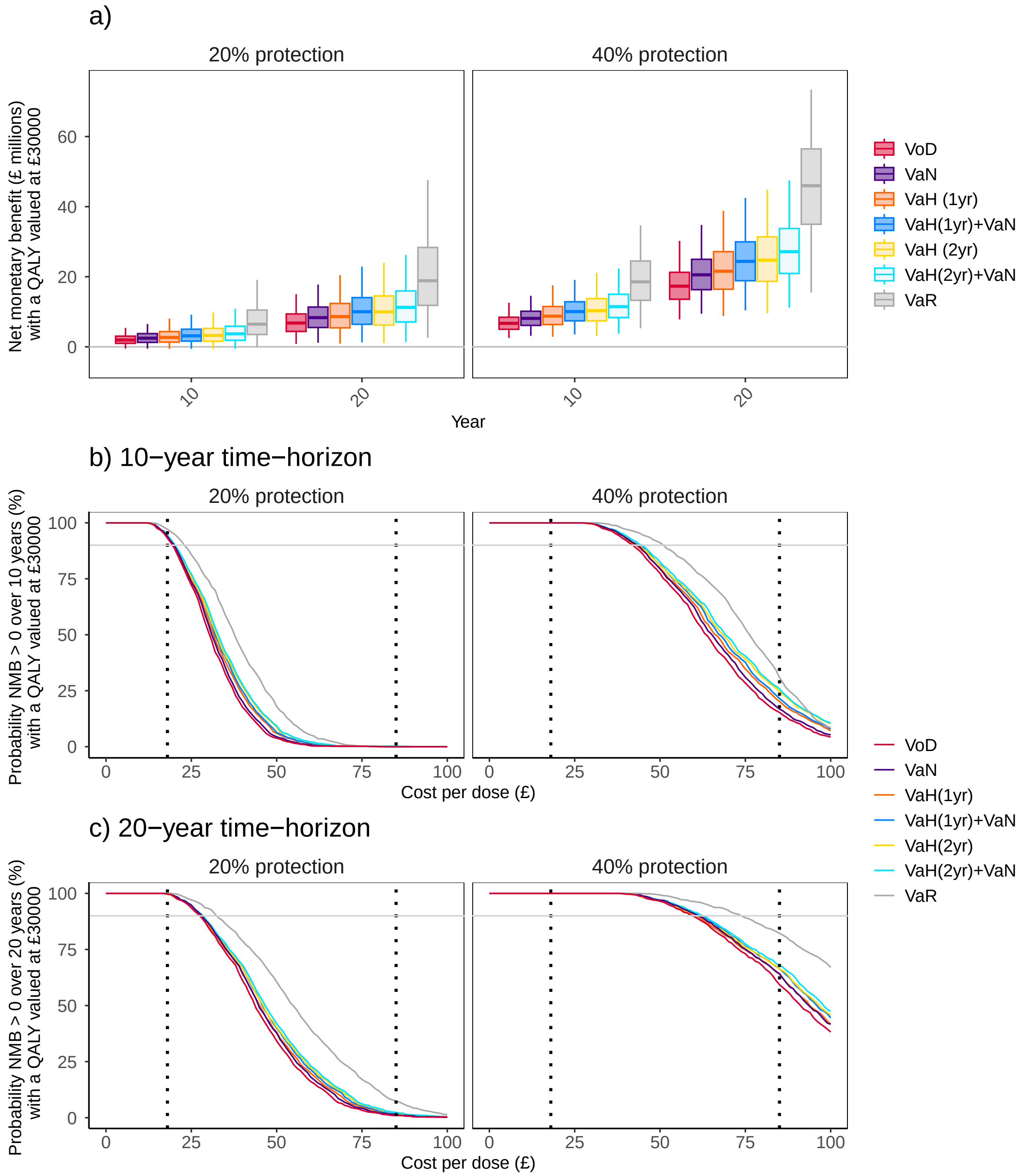

Supplementary Figure 7: Cost-effectiveness of vaccination: comparing time horizons of 10 & 20 years. Vaccination strategies considered are Vaccination-on-Diagnosis (VoD); Vaccination-according-to-partner-Notification (VaN); Vaccination-according-to-History (VaH) offering vaccination to those with a diagnosis in the last year [VaH(1yr)] or in the last two years [VaH(2yr)]; VaN combined with VaH(1yr) or VaH(2yr); and Vaccination-according-to-Risk (VaR). The left and right columns show results for vaccines providing 20% and 40% protection after two-dose primary vaccination, respectively. Primary vaccination has a 1.5-year duration of protection, and booster vaccination has a 3-year duration of protection. Section a) box plots of the net monetary benefit of each strategy over 10 years (our main scenario) and 20 years, for a vaccine costing £18/dose administered, with a QALY valued at £30,000. Box plot whiskers indicate the 2.5^{th} and 97.5^{th} centiles, boxes the 25th and 75th centiles, and the central line the median (50th centile). Sections b,c) probability that vaccination is cost-effective (i.e. net monetary benefit is positive) at different costs per dose administered, when evaluated over b) 10 years (presented in the main paper) or c) 20 years, with a QALY valued at £30,000. All plots use 1000 sets of sampled epidemiological and health-economic parameters.

Supplementary Table 5: Health-economic analysis of vaccination of MSM in England over 20 years, under different pragmatic vaccination strategies aiming to vaccinate high-risk MSM, for a vaccine with 20% protection after two doses, a 1.5-year duration of protection after primary vaccination, and a 3-year duration of protection after booster vaccination. Results are mean values and 95% credible intervals (CrIs) of simulations comparing each vaccination strategy against no vaccination, using 1000 sets of sampled epidemiological and health-economic parameters. All values are discounted at 3.5% per annum except where stated (cases averted and vaccine doses administered have both discounted and undiscounted numbers reported). Note that at £18/dose administered each strategy saves more money and gains more QALYs than the one above and therefore dominates the one above.

Strategy	Cases averted (thousands)		Testing & Treatment costs saved (£M)	QALYs gained	Vaccine doses administered (thousands)		£18/dose				£85/dose			
	Undisc.	Disc.			Undisc.	Disc.	Vaccination costs incurred (£M)	Net costs saved (£M)	$NMB_{£20k}$ (£M)<br>$NMB_{£30k}$ (£M)	Prob $NMB_{£20k}$>0<br>Prob $NMB_{£30k}$>0	Vaccination costs incurred (£M)	Net costs saved (£M)	$NMB_{£20k}$ (£M)<br>$NMB_{£30k}$ (£M)	Prob $NMB_{£20k}$>0<br>Prob $NMB_{£30k}$>0
VoD	90.8<br>(47.6, 145)	60.8<br>(32.1, 96.8)	9.8<br>(4.9, 16.3)	70.5<br>(34.1, 129.5)	363.4<br>(298.7, 424.4)	265.2<br>(218.6, 309.4)	4.8<br>(3.9, 5.6)	5<br>(-0.2, 11.6)	6.4<br>(0.5, 13.9)<br>7.1<br>(0.8, 15)	99.3%<br>99.7%	22.5<br>(18.6, 26.3)	-12.8<br>(-19.7, -5.4)	-11.4<br>(-18.8, -3.2)<br>-10.7<br>(-18.3, -2)	0.5%<br>1.0%
VaN	108.8<br>(57.1, 171.6)	73<br>(38.5, 114.9)	11.7<br>(6, 19.3)	84.7<br>(41, 154)	424.2<br>(346.5, 495.9)	311<br>(255.4, 362.9)	5.6<br>(4.6, 6.5)	6.1<br>(-0.1, 13.9)	7.8<br>(0.7, 16.4)<br>8.7<br>(1.2, 17.8)	99.6%<br>99.7%	26.4<br>(21.7, 30.8)	-14.7<br>(-23.1, -5.9)	-13<br>(-22.1, -3.2)<br>-12.2<br>(-21.6, -1.9)	0.7%<br>1.2%
VaH(1yr)	114<br>(53.4, 195.8)	76.9<br>(36.1, 133)	12.4<br>(5.6, 22.1)	89.2<br>(39, 170.9)	435.9<br>(377.9, 493.3)	321.3<br>(280.1, 363.2)	5.8<br>(5, 6.5)	6.6<br>(-0.2, 15.8)	8.4<br>(0.5, 18.8)<br>9.3<br>(0.9, 20.4)	99.3%<br>99.7%	27.3<br>(23.8, 30.9)	-15<br>(-22.5, -6.2)	-13.2<br>(-21.5, -2.9)<br>-12.3<br>(-21, -1.5)	0.8%<br>1.4%
VaH(1yr)+ VaN	129.6<br>(62.5, 215.8)	87.5<br>(42.2, 147.2)	14.1<br>(6.6, 24.4)	101.6<br>(45.2, 193)	487.7<br>(417.1, 555.8)	360.6<br>(312, 409.4)	6.5<br>(5.6, 7.4)	7.6<br>(-0.1, 17.7)	9.6<br>(0.8, 20.9)<br>10.6<br>(1.3, 22.9)	99.6%<br>99.7%	30.7<br>(26.5, 34.8)	-16.6<br>(-25.5, -6.3)	-14.6<br>(-24.3, -2.7)<br>-13.5<br>(-23.6, -1.1)	0.9%<br>1.8%
VaH(2yr)	130.2<br>(57.6, 228.8)	88.1<br>(38.8, 155.7)	14.2<br>(6, 25.5)	102.3<br>(41.8, 200.8)	484.2<br>(425.3, 549.2)	358.4<br>(315.2, 408)	6.5<br>(5.7, 7.3)	7.7<br>(-0.3, 18.8)	9.8<br>(0.5, 22.4)<br>10.8<br>(1.1, 24)	99.2%<br>99.7%	30.5<br>(26.8, 34.7)	-16.3<br>(-24.5, -6.1)	-14.3<br>(-23.4, -2.4)<br>-13.2<br>(-22.7, -0.7)	1.1%<br>2.3%
VaH(2yr)+ VaN	144.3<br>(66.6, 246.3)	97.8<br>(45, 167.9)	15.7<br>(7, 27.6)	113.4<br>(48.4, 217.5)	531.2<br>(462.8, 601.7)	394.2<br>(345.6, 445.3)	7.1<br>(6.2, 8)	8.6<br>(-0.1, 20.4)	10.9<br>(0.8, 24.3)<br>12<br>(1.4, 26.2)	99.5%<br>99.7%	33.5<br>(29.4, 37.9)	-17.8<br>(-27.4, -6.2)	-15.5<br>(-26, -2.1)<br>-14.4<br>(-25.4, -0.4)	1.2%<br>2.4%
VaR	226.9<br>(80, 426.8)	153.1<br>(53.9, 288.8)	24.6<br>(8.4, 47.8)	177.6<br>(57.9, 363.4)	693.4<br>(560.3, 827.3)	514.9<br>(410.5, 622.3)	9.3<br>(7.4, 11.2)	15.3<br>(0.9, 36.9)	18.9<br>(2, 43.8)<br>20.7<br>(2.6, 47.6)	100%<br>100%	43.8<br>(34.9, 52.9)	-19.2<br>(-28, -4.2)	-15.6<br>(-26.5, 2.6)<br>-13.8<br>(-25.8, 6.7)	4.1%<br>7.4%

Abbreviations: Undisc., Undiscounted; Disc., Discounted; M, millions; $NMB_{£20k}$, net monetary benefit with a QALY valued at £20,000; $NMB_{£30k}$, net monetary benefit with a QALY valued at £30,000 (shown in blue text); QALY, quality-adjusted life year; VoD, Vaccination-on-Diagnosis; VaN, Vaccination-according-to-partner-Notification; VaH(1yr), Vaccination-according-to-History (diagnosed in last year); VaH(2yr), Vaccination-according-to-History (diagnosed in last 2 years); VaR, Vaccination-according-to-Risk.

Supplementary Table 6: Health-economic analysis of vaccination of MSM in England over 20 years, under different pragmatic vaccination strategies aiming to vaccinate high-risk MSM, for a vaccine with 40% protection after two doses, a 1.5-year duration of protection after primary vaccination, and a 3-year duration of protection after booster vaccination. Results are mean values and 95% credible intervals (CrIs) of simulations comparing each vaccination strategy against no vaccination, using 1000 sets of sampled epidemiological and health-economic parameters. All values are discounted at 3.5% per annum except where stated (cases averted and vaccine doses administered have both discounted and undiscounted numbers reported). Note that at £18/dose administered each strategy saves more money and gains more QALYs than the one above and therefore dominates the one above.

Strategy	Cases averted (thousands)		Testing & Treatment costs saved (£M)	QALYs gained	Vaccine doses administered (thousands)		£18/dose				£85/dose			
	Undisc.	Disc.			Undisc.	Disc.	Vaccination costs incurred (£M)	Net costs saved (£M)	$NMB_{£20k}$ (£M)<br>$NMB_{£30k}$ (£M)	Prob $NMB_{£20k}$>0<br>Prob $NMB_{£30k}$>0	Vaccination costs incurred (£M)	Net costs saved (£M)	$NMB_{£20k}$ (£M)<br>$NMB_{£30k}$ (£M)	Prob $NMB_{£20k}$>0<br>Prob $NMB_{£30k}$>0
VoD	167<br>(99.5, 245.2)	112.5<br>(66.8, 165.6)	18.1<br>(10.2, 28.1)	130.4<br>(69.9, 228.6)	323.3<br>(255, 389.9)	238<br>(188.8, 286.2)	4.3<br>(3.4, 5.2)	13.8<br>(5.4, 24.1)	16.4<br>(6.9, 28.2)<br>17.7<br>(7.8, 30.2)	100%<br>100%	20.2<br>(16.1, 24.3)	-2.2<br>(-12.4, 9.3)	0.5<br>(-10.8, 13.3)<br>1.8<br>(-10.1, 15.3)	51.8%<br>59.3%
VaN	195.8<br>(117.6, 282.4)	132.2<br>(79.3, 191.4)	21.2<br>(12.2, 32.5)	153.3<br>(84.2, 262.3)	369.3<br>(287, 453.5)	273.4<br>(214.3, 334)	4.9<br>(3.9, 6)	16.3<br>(6.7, 27.8)	19.4<br>(8.5, 32.4)<br>20.9<br>(9.4, 34.8)	100%<br>100%	23.2<br>(18.2, 28.4)	-2<br>(-14.1, 11)	1.1<br>(-12, 15.5)<br>2.6<br>(-11.4, 17.7)	54.9%<br>64.2%
VaH(1yr)	206.1<br>(112.3, 317.2)	140<br>(75.8, 217.5)	22.5<br>(11.7, 36.6)	162.3<br>(79.6, 288.8)	382.9<br>(316.2, 445.2)	285<br>(238.6, 329.1)	5.1<br>(4.3, 5.9)	17.4<br>(6.2, 31.4)	20.6<br>(7.9, 36.2)<br>22.2<br>(8.8, 38.8)	100%<br>100%	24.2<br>(20.3, 28)	-1.7<br>(-13.9, 13.1)	1.5<br>(-12, 18.2)<br>3.1<br>(-11.2, 21.1)	55.0%<br>63.9%
VaH(1yr)+ VaN	229.8<br>(130.4, 342.2)	156.4<br>(88.2, 235.5)	25.1<br>(13.6, 39.5)	181.3<br>(93.7, 321.2)	420.2<br>(339.3, 499.2)	314<br>(257.5, 368.4)	5.7<br>(4.6, 6.6)	19.5<br>(7.4, 34)	23.1<br>(9.5, 40)<br>24.9<br>(10.4, 42.5)	100%<br>100%	26.7<br>(21.9, 31.3)	-1.6<br>(-15.1, 14.3)	2.1<br>(-13.1, 19.9)<br>3.9<br>(-12.3, 22.7)	57.8%<br>66.4%
VaH(2yr)	234.2<br>(122.4, 364.5)	159.7<br>(82.7, 251.8)	25.7<br>(12.9, 42.2)	185.1<br>(88, 335.8)	422.4<br>(351.7, 488.8)	316<br>(268.5, 361.6)	5.7<br>(4.8, 6.5)	20<br>(6.8, 36.2)	23.7<br>(8.8, 41.8)<br>25.5<br>(9.6, 44.8)	100%<br>100%	26.9<br>(22.8, 30.7)	-1.2<br>(-14.8, 16)	2.5<br>(-12.9, 21.9)<br>4.4<br>(-12, 25.2)	58.8%<br>66.3%
VaH(2yr)+ VaN	254.9<br>(139.6, 383.8)	174.1<br>(94.4, 265.1)	28<br>(14.7, 44.8)	201.7<br>(100.3, 358.3)	455<br>(369.8, 538.2)	341.6<br>(284.5, 399)	6.1<br>(5.1, 7.2)	21.8<br>(8, 38.4)	25.9<br>(10.2, 44.6)<br>27.9<br>(11.2, 47.5)	100%<br>100%	29<br>(24.2, 33.9)	-1.1<br>(-16.2, 17)	3<br>(-14, 23.2)<br>5<br>(-13.1, 26.4)	61.1%<br>67.9%
VaR	409.8<br>(179, 588.6)	278<br>(120.3, 406)	44.7<br>(18.6, 71)	322<br>(129.8, 561.6)	666.2<br>(543.9, 809.2)	495.9<br>(399.3, 608.3)	8.9<br>(7.2, 10.9)	35.7<br>(11.3, 60.4)	42.2<br>(14.1, 68.9)<br>45.4<br>(15.5, 73.4)	100%<br>100%	42.1<br>(33.9, 51.7)	2.5<br>(-15.4, 22.4)	9<br>(-13, 31)<br>12.2<br>(-12, 35.1)	76.1%<br>82.2%

Abbreviations: Undisc., Undiscounted; Disc., Discounted; M, millions; $NMB_{£20k}$, net monetary benefit with a QALY valued at £20,000; $NMB_{£30k}$, net monetary benefit with a QALY valued at £30,000 (shown in blue text); QALY, quality-adjusted life year; VoD, Vaccination-on-Diagnosis; VaN, Vaccination-according-to-partner-Notification; VaH(1yr), Vaccination-according-to-History (diagnosed in last year); VaH(2yr), Vaccination-according-to-History (diagnosed in last 2 years); VaR, Vaccination-according-to-Risk.

2.6 Considering vaccines with longer durations of protection (Supplementary Tables 7-11, Supplementary Figure 8)

Supplementary Figure 8a shows net monetary benefit boxplots, and Supplementary Figure 8b-d shows the probability that vaccination is cost-effective at different costs per dose administered, if the duration of protection is 1.5 years after primary vaccination and 3 years after booster vaccination (as in the main paper) or 4 years after primary and booster vaccination, or 7.5 years after primary and booster vaccination.

The maximum costs per dose administered at which vaccination would be cost-effective for the lowest and highest value strategies are shown in Supplementary Table 7. The lowest value strategy varies between scenarios, but VaR is the highest value strategy (i.e. has the highest cost per dose administered for vaccination to be cost-effective over 10 years) in all scenarios. The corresponding maximum costs for not listed in Supplementary Table 7 between the values listed in all cases.

Supplementary Table 7: Maximum costs per dose administered at which vaccination would be cost-effective over 10 years for vaccines with different durations of protection.

Level of protection	Duration of protection	Maximum cost per dose*	
		**Lowest value strategy**	**Highest value strategy**
20%	1.5, 3 years	£19 [VoD, VaN, VaH(1yr)]	£22 [VaR]
20%	4 years	£29 [all except for VaR]	£32 [VaR]
20%	7.5 years	£36 [VaH, VaH+VaN] †	£40 [VaR]
40%	1.5, 3 years	£42 [VoD, VaH(1yr)]	£50 [VaR]
40%	4 years	£64 [VaH(2yr)]	£72 [VaR]
40%	7.5 years	£83 [all except for VaR]	£92 [VaR]

* i.e. when Prob($\text{NMB}_{£20k} > 0$) $> 50\%$ and Prob($\text{NMB}_{£30k} > 0$) $> 90\%$
†with both a one-year and two-year history as eligibility criteria.

Supplementary Tables 8 and 9 show health-economic analysis for a vaccine with 4-year durations of protection, while Supplementary Tables 10 and 11 show health-economic analysis for a vaccine with 7.5-year durations of protection. At 20% protection no strategy was likely to be cost-effective at £85/dose administered with either 4-year durations of protection (Supplementary Table 8) or 7.5-year durations of protection (Supplementary Table 10). At 40% protection and 4-year durations of protection (Supplementary Table 9) at £85/dose administered, all strategies had over 50% probability that $\text{NMB}_{£20k} > 0$ (ranging from 64.2% [VoD] to 71.7% [VaR]), but no strategy had over 90% probability that $\text{NMB}_{£30k} > 0$ (ranging from 70.2% [VoD] to 78.5% [VaR]). At 40% protection and 7.5-year durations of protection (Supplementary Table 11) at £85/dose administered, all strategies again had over 50% probability that $\text{NMB}_{£20k} > 0$, but only VaR had over 90% probability that $\text{NMB}_{£30k} > 0$ (though note that all strategies were close; the strategy least likely to have $\text{NMB}_{£30k} > 0$ was VaH(2yr), which had 89% probability).

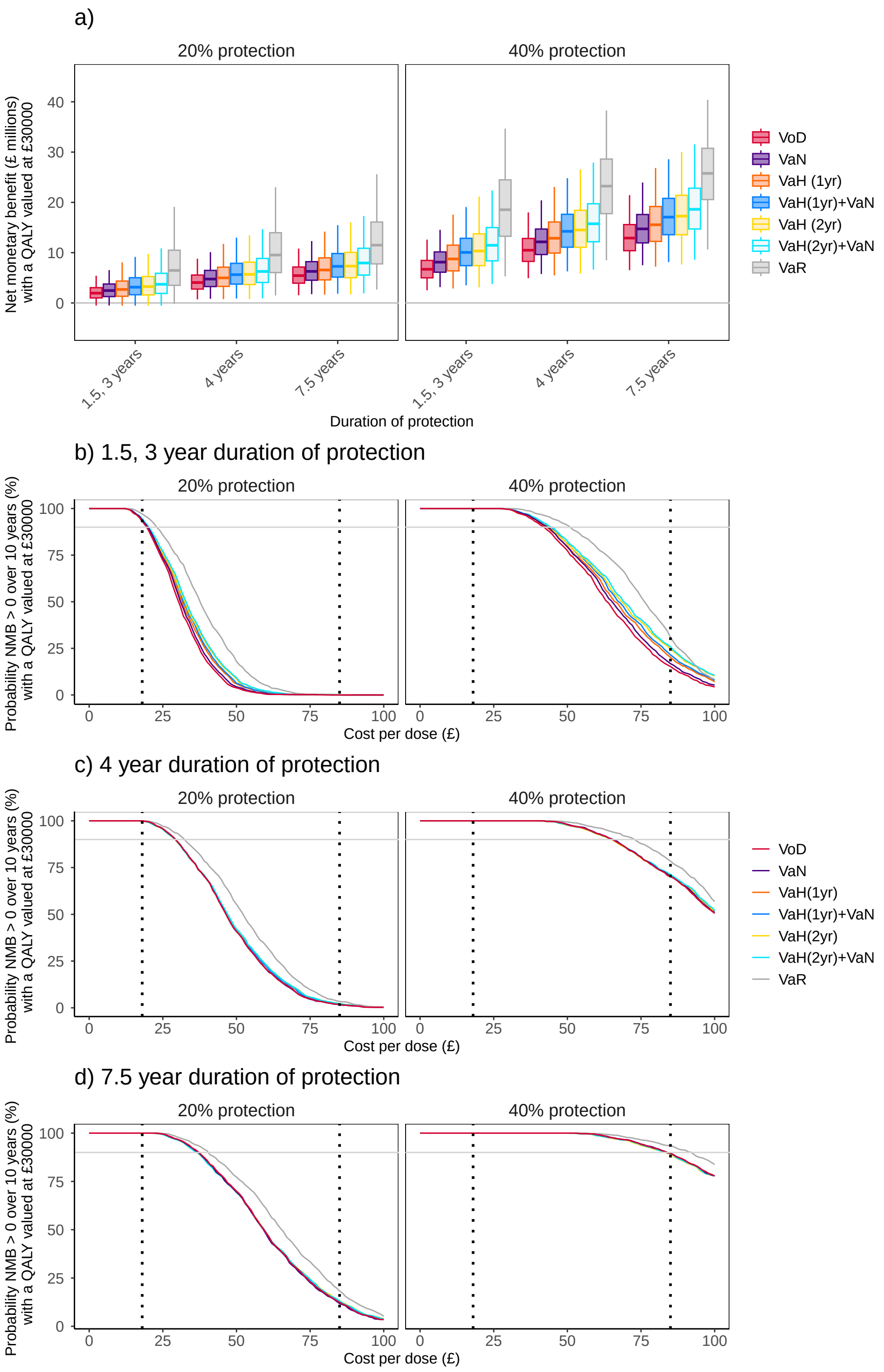

Supplementary Figure 8: Cost-effectiveness of vaccination with different durations of protection. Vaccination strategies considered are Vaccination-on-Diagnosis (VoD); Vaccination-according-to-partner-Notification (VaN); Vaccination-according-to-History (VaH) offering vaccination to those with a diagnosis in the last year [VaH(1yr)] or in the last two years [VaH(2yr)]; VaN combined with VaH(1yr) or VaH(2yr); and Vaccination-according-to-Risk (VaR). The left and right columns show results for vaccines providing 20% and 40% protection after two-dose primary vaccination, respectively. Section a) box plots of the net monetary benefit of each strategy over 10 years, for a vaccine costing £18/dose administered and with a QALY valued at £30,000, with a duration of protection of 1.5 years after primary vaccination and 3 years after booster vaccination (our main scenario), with 4 years protection after primary and booster vaccination, and with 7.5 years protection after primary and booster vaccination. Box plot whiskers indicate the 2.5^{th} and 97.5^{th} centiles, boxes the 25th and 75th centiles, and the central line the median (50th centile). Sections b-d) probability that vaccination is cost-effective (i.e. net monetary benefit is positive) when evaluated over 10 years at different costs per dose administered, for a vaccine with b) 1.5-year duration of protection from primary vaccination and 3 years after booster vaccination (presented in the main paper) c) 4-year durations of protection and d) 7.5-year durations of protection. All plots use 1000 sets of sampled epidemiological and health-economic parameters.

Supplementary Table 8: Health-economic analysis of vaccination of MSM in England over 10 years, under different pragmatic vaccination strategies aiming to vaccinate high-risk MSM, for a vaccine with 20% protection after two doses and a 4-year duration of protection after both primary vaccination and after booster vaccination. Results are mean values and 95% credible intervals (CrIs) of simulations comparing each vaccination strategy against no vaccination, using 1000 sets of sampled epidemiological and health-economic parameters. All values are discounted at 3.5% per annum except where stated (cases averted and vaccine doses administered have both discounted and undiscounted numbers reported). Note that at £18/dose administered each strategy saves more money and gains more QALYs than the one above and therefore dominates the one above.

Strategy	Cases averted (thousands)		Testing & Treatment costs saved (£M)	QALYs gained	Vaccine doses administered (thousands)		£18/dose				£85/dose			
	Undisc.	Disc.			Undisc.	Disc.	Vaccination costs incurred (£M)	Net costs saved (£M)	$NMB_{£20k}$ (£M)<br>$NMB_{£30k}$ (£M)	Prob $NMB_{£20k}$>0<br>Prob $NMB_{£30k}$>0	Vaccination costs incurred (£M)	Net costs saved (£M)	$NMB_{£20k}$ (£M)<br>$NMB_{£30k}$ (£M)	Prob $NMB_{£20k}$>0<br>Prob $NMB_{£30k}$>0
VoD	42.9<br>(23, 67.9)	34.7<br>(18.7, 54.9)	5.6<br>(2.9, 9.2)	40.8<br>(19.8, 74)	168.6<br>(139.2, 197.5)	143.8<br>(119.1, 167.9)	2.6<br>(2.1, 3)	3<br>(0.1, 6.8)	3.8<br>(0.5, 8.1)<br>4.2<br>(0.7, 8.8)	99.7%<br>100%	12.2<br>(10.1, 14.3)	-6.6<br>(-10.5, -2.5)	-5.8<br>(-10, -1.2)<br>-5.4<br>(-9.7, -0.5)	0.9%<br>1.7%
VaN	50.4<br>(27, 78.8)	40.8<br>(21.9, 63.9)	6.6<br>(3.4, 10.8)	47.9<br>(23.5, 86.2)	197.9<br>(162, 231.9)	169.2<br>(139, 197.8)	3<br>(2.5, 3.6)	3.5<br>(0.1, 7.9)	4.5<br>(0.6, 9.3)<br>5<br>(0.8, 10.1)	99.7%<br>100%	14.4<br>(11.8, 16.8)	-7.8<br>(-12.4, -2.9)	-6.8<br>(-11.9, -1.4)<br>-6.4<br>(-11.6, -0.6)	0.9%<br>1.7%
VaH(1yr)	53.6<br>(25.5, 91.4)	43.4<br>(20.7, 74.1)	7<br>(3.2, 12.3)	51<br>(22.6, 97.4)	205.7<br>(179, 233.2)	176.4<br>(153.5, 201.4)	3.2<br>(2.8, 3.6)	3.8<br>(0.1, 9.1)	4.9<br>(0.5, 10.8)<br>5.4<br>(0.7, 11.7)	99.7%<br>99.9%	15<br>(13, 17.1)	-8<br>(-12.1, -3)	-7<br>(-11.5, -1.4)<br>-6.4<br>(-11.2, -0.4)	1.1%<br>1.9%
VaH(1yr)+ VaN	60<br>(29.4, 99.8)	48.7<br>(23.9, 81.1)	7.9<br>(3.7, 13.5)	57.2<br>(25.7, 107)	231<br>(200.1, 262.5)	198.5<br>(172.6, 226.1)	3.6<br>(3.1, 4.1)	4.3<br>(0.1, 10.1)	5.4<br>(0.6, 11.8)<br>6<br>(0.9, 13)	99.7%<br>99.9%	16.9<br>(14.7, 19.2)	-9<br>(-13.8, -3.4)	-7.9<br>(-13.2, -1.6)<br>-7.3<br>(-12.9, -0.4)	1.1%<br>2.0%
VaH(2yr)	60.5<br>(27.3, 105.5)	49.1<br>(22.2, 85.9)	7.9<br>(3.4, 14.1)	57.6<br>(24.1, 112.3)	229.4<br>(201.8, 261.4)	197.2<br>(172.7, 223.8)	3.5<br>(3.1, 4)	4.4<br>(0, 10.6)	5.5<br>(0.5, 12.6)<br>6.1<br>(0.8, 13.4)	99.6%<br>99.9%	16.8<br>(14.7, 19)	-8.8<br>(-13.2, -3.2)	-7.7<br>(-12.5, -1.4)<br>-7.1<br>(-12.2, -0.2)	1.2%<br>2.2%
VaH(2yr)+ VaN	66.4<br>(30.9, 113)	53.9<br>(25.1, 91.9)	8.7<br>(3.9, 15.2)	63.3<br>(27.3, 120.7)	252.6<br>(222.5, 285.7)	217.5<br>(191, 245.2)	3.9<br>(3.4, 4.4)	4.8<br>(0.1, 11.5)	6.1<br>(0.6, 13.5)<br>6.7<br>(0.9, 14.7)	99.6%<br>99.9%	18.5<br>(16.2, 20.8)	-9.8<br>(-14.8, -3.4)	-8.5<br>(-14.1, -1.6)<br>-7.9<br>(-13.8, -0.3)	1.4%<br>2.1%
VaR	96<br>(36.1, 175.6)	77.8<br>(29.3, 142.2)	12.6<br>(4.6, 23.9)	91.3<br>(31.8, 182.7)	323.8<br>(259.8, 388.1)	279.2<br>(222, 337.9)	5<br>(4, 6.1)	7.5<br>(0.6, 18.2)	9.4<br>(1.2, 21.3)<br>10.3<br>(1.5, 23)	100%<br>100%	23.7<br>(18.9, 28.7)	-11.2<br>(-15.2, -4)	-9.3<br>(-14.4, -0.7)<br>-8.4<br>(-14, 1.2)	1.8%<br>3.4%

Abbreviations: Undisc., Undiscounted; Disc., Discounted; M, millions; $NMB_{£20k}$, net monetary benefit with a QALY valued at £20,000; $NMB_{£30k}$, net monetary benefit with a QALY valued at £30,000 (shown in blue text); QALY, quality-adjusted life year; VoD, Vaccination-on-Diagnosis; VaN, Vaccination-according-to-partner-Notification; VaH(1yr), Vaccination-according-to-History (diagnosed in last year); VaH(2yr), Vaccination-according-to-History (diagnosed in last 2 years); VaR, Vaccination-according-to-Risk.

Supplementary Table 9: Health-economic analysis of vaccination of MSM in England over 10 years, under different pragmatic vaccination strategies aiming to vaccinate high-risk MSM, for a vaccine with 40% protection after two doses and a 4-year duration of protection after both primary vaccination and after booster vaccination. Results are mean values and 95% credible intervals (CrIs) of simulations comparing each vaccination strategy against no vaccination, using 1000 sets of sampled epidemiological and health-economic parameters. All values are discounted at 3.5% per annum except where stated (cases averted and vaccine doses administered have both discounted and undiscounted numbers reported). Note that at £18/dose administered each strategy saves more money and gains more QALYs than the one above and therefore dominates the one above.

Strategy	Cases averted (thousands)		Testing & Treatment costs saved (£M)	QALYs gained	Vaccine doses administered (thousands)		£18/dose				£85/dose			
	Undisc.	Disc.			Undisc.	Disc.	Vaccination costs incurred (£M)	Net costs saved (£M)	NMB$_{£20k}$ (£M)<br>NMB$_{£30k}$ (£M)	Prob NMB$_{£20k}$>0<br>Prob NMB$_{£30k}$>0	Vaccination costs incurred (£M)	Net costs saved (£M)	NMB$_{£20k}$ (£M)<br>NMB$_{£30k}$ (£M)	Prob NMB$_{£20k}$>0<br>Prob NMB$_{£30k}$>0
VoD	81.7<br>(48.6, 119.9)	66.3<br>(39.5, 97.3)	10.7<br>(6.1, 16.5)	77.8<br>(41.8, 133.8)	150.1<br>(118.5, 181.5)	128.8<br>(102.4, 155.4)	2.3<br>(1.8, 2.8)	8.4<br>(3.5, 14.4)	9.9<br>(4.4, 16.9)<br>10.7<br>(4.9, 18)	100%<br>100%	11<br>(8.7, 13.2)	-0.2<br>(-6.2, 6.7)	1.3<br>(-5.3, 9)<br>2.1<br>(-4.8, 10.3)	64.2%<br>70.2%
VaN	94.1<br>(56.7, 136.6)	76.4<br>(46.1, 111.3)	12.3<br>(7.1, 18.8)	89.6<br>(49.2, 151.8)	172.7<br>(134.4, 212.4)	148.6<br>(116.6, 182.1)	2.7<br>(2.1, 3.3)	9.7<br>(4.1, 16.4)	11.5<br>(5.2, 19.2)<br>12.4<br>(5.8, 20.4)	100%<br>100%	12.6<br>(9.9, 15.5)	-0.3<br>(-7.2, 7.4)	1.5<br>(-6, 10.2)<br>2.4<br>(-5.5, 11.5)	64.8%<br>70.3%
VaH(1yr)	100.5<br>(54.4, 156.3)	81.8<br>(44.3, 127.1)	13.2<br>(6.9, 21.4)	95.9<br>(46.9, 171.1)	181.1<br>(151.5, 210.2)	156.3<br>(132.2, 181.1)	2.8<br>(2.4, 3.3)	10.4<br>(3.9, 18.8)	12.3<br>(5, 21.7)<br>13.3<br>(5.5, 23.1)	100%<br>100%	13.3<br>(11.2, 15.4)	-0.1<br>(-7.1, 8.8)	1.8<br>(-6, 11.8)<br>2.8<br>(-5.5, 13.3)	64.6%<br>70.4%
VaH(1yr)+ VaN	110.8<br>(62.6, 166.7)	90.2<br>(51, 136.2)	14.6<br>(7.9, 23)	105.7<br>(53.8, 184.5)	199.6<br>(163.1, 235.4)	172.8<br>(143, 202.3)	3.1<br>(2.6, 3.6)	11.4<br>(4.5, 20)	13.6<br>(5.7, 23.4)<br>14.6<br>(6.3, 24.8)	100%<br>100%	14.7<br>(12.2, 17.2)	-0.1<br>(-8, 9.5)	2<br>(-6.7, 12.5)<br>3<br>(-6.2, 14.2)	64.9%<br>70.6%
VaH(2yr)	113.2<br>(58.7, 178.6)	92.2<br>(47.8, 145.6)	14.9<br>(7.5, 24.5)	108<br>(51.5, 196.2)	201.4<br>(172.3, 231)	174.5<br>(151.5, 199.6)	3.1<br>(2.7, 3.6)	11.7<br>(4.2, 21.5)	13.9<br>(5.3, 24.8)<br>15<br>(5.9, 26.6)	100%<br>100%	14.8<br>(12.9, 17)	0<br>(-7.7, 10.2)	2.2<br>(-6.6, 13.6)<br>3.3<br>(-6, 15.6)	65.1%<br>70.6%
VaH(2yr)+ VaN	122.4<br>(66.1, 187.5)	99.7<br>(53.9, 153.1)	16.1<br>(8.4, 25.7)	116.8<br>(57.4, 207.4)	217.9<br>(181.7, 255)	189.2<br>(160.7, 220.3)	3.4<br>(2.9, 4)	12.7<br>(4.7, 22.5)	15<br>(6, 26.1)<br>16.2<br>(6.6, 27.9)	100%<br>100%	16.1<br>(13.7, 18.7)	0<br>(-8.6, 10.8)	2.3<br>(-7.3, 14.2)<br>3.5<br>(-6.7, 16.1)	65.2%<br>71.3%
VaR	175.9<br>(79.1, 261.2)	142.9<br>(64.3, 213.8)	23.1<br>(10.1, 37.1)	167.5<br>(70.8, 291.2)	312<br>(251.9, 378.8)	269.4<br>(215.6, 329.7)	4.8<br>(3.9, 5.9)	18.2<br>(6.2, 31.7)	21.6<br>(7.8, 35.9)<br>23.2<br>(8.5, 38.3)	100%<br>100%	22.9<br>(18.3, 28)	0.2<br>(-8.4, 10.8)	3.5<br>(-6.9, 15.3)<br>5.2<br>(-6.3, 17.7)	71.7%<br>78.5%

Abbreviations: Undisc., Undiscounted; Disc., Discounted; M, millions; NMB$_{£20k}$, net monetary benefit with a QALY valued at £20,000; NMB$_{£30k}$, net monetary benefit with a QALY valued at £30,000 (shown in blue text); QALY, quality-adjusted life year; VoD, Vaccination-on-Diagnosis; VaN, Vaccination-according-to-partner-Notification; VaH(1yr), Vaccination-according-to-History (diagnosed in last year); VaH(2yr), Vaccination-according-to-History (diagnosed in last 2 years); VaR, Vaccination-according-to-Risk.

Supplementary Table 10: Health-economic analysis of vaccination of MSM in England over 10 years, under different pragmatic vaccination strategies aiming to vaccinate high-risk MSM, for a vaccine with 20% protection after two doses and a 7.5-year duration of protection after both primary vaccination and after booster vaccination. Results are mean values and 95% credible intervals (CrIs) of simulations comparing each vaccination strategy against no vaccination, using 1000 sets of sampled epidemiological and health-economic parameters. All values are discounted at 3.5% per annum except where stated (cases averted and vaccine doses administered have both discounted and undiscounted numbers reported). Note that at £18/dose administered each strategy saves more money and gains more QALYs than the one above and therefore dominates the one above.

Strategy	Cases averted (thousands)		Testing & Treatment costs saved (£M)	QALYs gained	Vaccine doses administered (thousands)		£18/dose				£85/dose			
	Undisc.	Disc.			Undisc.	Disc.	Vaccination costs incurred (£M)	Net costs saved (£M)	$NMB_{£20k}$ (£M)<br>$NMB_{£30k}$ (£M)	Prob $NMB_{£20k}>0$<br>Prob $NMB_{£30k}>0$	Vaccination costs incurred (£M)	Net costs saved (£M)	$NMB_{£20k}$ (£M)<br>$NMB_{£30k}$ (£M)	Prob $NMB_{£20k}>0$<br>Prob $NMB_{£30k}>0$
VoD	50.6<br>(27.1, 79.7)	40.8<br>(22, 64.2)	6.6<br>(3.4, 10.8)	47.9<br>(23.5, 86.3)	154.5<br>(126.7, 182.1)	132.7<br>(109.5, 156)	2.4<br>(2, 2.8)	4.2<br>(0.8, 8.6)	5.2<br>(1.3, 10)<br>5.6<br>(1.5, 10.8)	100%<br>100%	11.3<br>(9.3, 13.3)	-4.7<br>(-8.9, 0.2)	-3.7<br>(-8.3, 1.6)<br>-3.3<br>(-8, 2.3)	7.8%<br>12.4%
VaN	58.5<br>(31.4, 91.2)	47.2<br>(25.4, 73.5)	7.6<br>(4, 12.5)	55.5<br>(27.3, 98.8)	179.5<br>(145.7, 211.5)	154.6<br>(125.9, 182.2)	2.8<br>(2.3, 3.3)	4.8<br>(0.9, 9.9)	6<br>(1.4, 11.5)<br>6.5<br>(1.7, 12.3)	100%<br>100%	13.1<br>(10.7, 15.5)	-5.5<br>(-10.4, 0.2)	-4.4<br>(-9.9, 1.8)<br>-3.8<br>(-9.5, 2.7)	7.4%<br>11.8%
VaH(1yr)	61.9<br>(29.9, 104.4)	50<br>(24.2, 84.3)	8.1<br>(3.7, 14.1)	58.8<br>(26.3, 110.4)	186<br>(162.5, 210.6)	160.6<br>(140.5, 181.8)	2.9<br>(2.5, 3.3)	5.2<br>(0.8, 11.2)	6.4<br>(1.4, 13.1)<br>7<br>(1.7, 14.2)	100%<br>100%	13.6<br>(11.9, 15.5)	-5.6<br>(-10.2, 0.3)	-4.4<br>(-9.6, 2.2)<br>-3.8<br>(-9.2, 3.2)	8.4%<br>12.7%
VaH(1yr)+ VaN	68.7<br>(33.8, 113.1)	55.5<br>(27.4, 91.7)	9<br>(4.3, 15.3)	65.2<br>(29.6, 120.4)	207.2<br>(179.1, 239.2)	179.3<br>(155.6, 205.7)	3.2<br>(2.8, 3.7)	5.7<br>(0.9, 12.3)	7<br>(1.5, 14.2)<br>7.7<br>(1.8, 15.5)	100%<br>100%	15.2<br>(13.2, 17.5)	-6.3<br>(-11.6, 0.4)	-5<br>(-11, 2.3)<br>-4.3<br>(-10.5, 3.4)	8.1%<br>12.4%
VaH(2yr)	68.9<br>(31.7, 118.6)	55.7<br>(25.7, 96.2)	9<br>(3.9, 15.8)	65.4<br>(27.8, 125.5)	205.4<br>(181.3, 233.7)	177.9<br>(157, 202.1)	3.2<br>(2.8, 3.6)	5.8<br>(0.8, 12.7)	7.1<br>(1.4, 15)<br>7.8<br>(1.7, 16)	100%<br>100%	15.1<br>(13.3, 17.2)	-6.1<br>(-11.2, 0.5)	-4.8<br>(-10.3, 2.6)<br>-4.2<br>(-10, 3.8)	8.7%<br>13.2%
VaH(2yr)+ VaN	75<br>(35.2, 126.1)	60.7<br>(28.6, 102.4)	9.8<br>(4.5, 16.9)	71.3<br>(31.1, 133.3)	224.7<br>(197.4, 256.9)	195<br>(171.8, 221.9)	3.5<br>(3.1, 4)	6.3<br>(0.9, 13.7)	7.7<br>(1.6, 15.9)<br>8.4<br>(1.9, 17.3)	100%<br>100%	16.6<br>(14.6, 18.9)	-6.8<br>(-12.5, 0.6)	-5.3<br>(-11.6, 2.7)<br>-4.6<br>(-11.2, 4)	8.6%<br>13.1%
VaR	103.9<br>(40.9, 182.5)	84.1<br>(33.1, 147.8)	13.6<br>(5.2, 25.6)	98.8<br>(36, 194)	278.7<br>(231.5, 322)	242.7<br>(199.6, 284.4)	4.4<br>(3.6, 5.1)	9.2<br>(1.5, 20.3)	11.2<br>(2.3, 23.7)<br>12.2<br>(2.7, 25.6)	100%<br>100%	20.6<br>(17, 24.2)	-7.1<br>(-12.4, 1.4)	-5.1<br>(-11.6, 5)<br>-4.1<br>(-11.1, 7.1)	12.6%<br>18.3%

Abbreviations: Undisc., Undiscounted; Disc., Discounted; M, millions; $NMB_{£20k}$, net monetary benefit with a QALY valued at £20,000; $NMB_{£30k}$, net monetary benefit with a QALY valued at £30,000 (shown in blue text); QALY, quality-adjusted life year; VoD, Vaccination-on-Diagnosis; VaN, Vaccination-according-to-partner-Notification; VaH(1yr), Vaccination-according-to-History (diagnosed in last year); VaH(2yr), Vaccination-according-to-History (diagnosed in last 2 years); VaR, Vaccination-according-to-Risk.

Supplementary Table 11: Health-economic analysis of vaccination of MSM in England over 10 years, under different pragmatic vaccination strategies aiming to vaccinate high-risk MSM, for a vaccine with 40% protection after two doses and a 7.5-year duration of protection after both primary vaccination and after booster vaccination. Results are mean values and 95% credible intervals (CrIs) of simulations comparing each vaccination strategy against no vaccination, using 1000 sets of sampled epidemiological and health-economic parameters. All values are discounted at 3.5% per annum except where stated (cases averted and vaccine doses administered have both discounted and undiscounted numbers reported). Note that at £18/dose administered each strategy saves more money and gains more QALYs than the one above and therefore dominates the one above.

Strategy	Cases averted (thousands)		Testing & Treatment costs saved (£M)	QALYs gained	Vaccine doses administered (thousands)		£18/dose				£85/dose			
	Undisc.	Disc.			Undisc.	Disc.	Vaccination costs incurred (£M)	Net costs saved (£M)	NMB$_{£20k}$ (£M)<br>NMB$_{£30k}$ (£M)	Prob NMB$_{£20k}$>0<br>Prob NMB$_{£30k}$>0	Vaccination costs incurred (£M)	Net costs saved (£M)	NMB$_{£20k}$ (£M)<br>NMB$_{£30k}$ (£M)	Prob NMB$_{£20k}$>0<br>Prob NMB$_{£30k}$>0
VoD	96<br>(57.8, 139.3)	77.6<br>(46.7, 112.8)	12.5<br>(7.2, 19.2)	91.2<br>(49.4, 154.3)	134.9<br>(105.2, 164.8)	116.7<br>(91.6, 141.7)	2.1<br>(1.6, 2.6)	10.4<br>(4.8, 17.2)	12.3<br>(5.9, 20)<br>13.2<br>(6.5, 21.4)	100%<br>100%	9.9<br>(7.8, 12)	2.6<br>(-4.1, 10.4)	4.4<br>(-3.1, 13)<br>5.4<br>(-2.5, 14.3)	85.3%<br>89.5%
VaN	109.1<br>(66.5, 157)	88.3<br>(53.9, 127.2)	14.3<br>(8.4, 21.7)	103.6<br>(57.4, 174.3)	153.3<br>(118, 190.9)	133.1<br>(103.4, 164.9)	2.4<br>(1.9, 3)	11.9<br>(5.6, 19.4)	13.9<br>(6.8, 22.3)<br>15<br>(7.5, 24)	100%<br>100%	11.3<br>(8.8, 14)	2.9<br>(-4.7, 11.6)	5<br>(-3.4, 14.6)<br>6<br>(-2.8, 15.9)	85.3%<br>89.5%
VaH(1yr)	115.9<br>(64.4, 175.6)	93.9<br>(52.2, 142.7)	15.2<br>(8.1, 24.1)	110.2<br>(54.8, 193.2)	160.4<br>(131.2, 189.7)	139.7<br>(115.9, 163.6)	2.5<br>(2.1, 2.9)	12.6<br>(5.3, 21.7)	14.9<br>(6.6, 25.3)<br>16<br>(7.2, 26.8)	100%<br>100%	11.9<br>(9.9, 13.9)	3.3<br>(-4.6, 13.2)	5.5<br>(-3.4, 16.3)<br>6.6<br>(-3, 18.3)	84.9%<br>89.3%
VaH(1yr)+ VaN	126.5<br>(72.4, 187.4)	102.6<br>(58.8, 152.3)	16.6<br>(9.1, 25.9)	120.4<br>(62, 207.3)	175.1<br>(141, 212)	153.1<br>(125, 182.8)	2.8<br>(2.3, 3.3)	13.8<br>(6, 23.2)	16.2<br>(7.5, 27)<br>17.4<br>(8.1, 28.6)	100%<br>100%	13<br>(10.6, 15.5)	3.6<br>(-5.2, 14)	6<br>(-3.9, 17.5)<br>7.2<br>(-3.3, 19.2)	85.0%<br>89.5%
VaH(2yr)	128.5<br>(69.1, 196.3)	104.3<br>(55.9, 159.6)	16.8<br>(8.6, 27.1)	122.3<br>(59.4, 217.5)	176.9<br>(148.5, 207)	154.8<br>(132.3, 179.9)	2.8<br>(2.4, 3.2)	14<br>(5.7, 24.5)	16.5<br>(7, 28.2)<br>17.7<br>(7.6, 30)	100%<br>100%	13.2<br>(11.2, 15.3)	3.7<br>(-5.1, 14.7)	6.1<br>(-3.8, 18.6)<br>7.3<br>(-3.3, 20.4)	84.5%<br>89.0%
VaH(2yr)+ VaN	137.8<br>(76.5, 205.7)	111.9<br>(62.1, 167.2)	18.1<br>(9.6, 28.5)	131.2<br>(65.8, 227.3)	189.9<br>(155.8, 226.4)	166.6<br>(139.3, 196.4)	3<br>(2.5, 3.5)	15.1<br>(6.3, 25.6)	17.7<br>(7.9, 29.6)<br>19<br>(8.6, 31.5)	100%<br>100%	14.2<br>(11.8, 16.7)	3.9<br>(-5.6, 15.6)	6.5<br>(-4.2, 19.3)<br>7.8<br>(-3.5, 21.3)	84.8%<br>89.3%
VaR	186.7<br>(90.3, 264.9)	151.7<br>(73.3, 216.9)	24.5<br>(11.3, 38.1)	177.8<br>(80.3, 300)	267.9<br>(224.3, 314.8)	233.7<br>(193.4, 277.7)	4.2<br>(3.5, 5)	20.3<br>(8, 33.2)	23.8<br>(9.6, 37.8)<br>25.6<br>(10.6, 40.4)	100%<br>100%	19.9<br>(16.4, 23.6)	4.6<br>(-5, 15.6)	8.2<br>(-3.3, 20.4)<br>9.9<br>(-2.6, 22.6)	90.8%<br>93.5%

Abbreviations: Undisc., Undiscounted; Disc., Discounted; M, millions; NMB$_{£20k}$, net monetary benefit with a QALY valued at £20,000; NMB$_{£30k}$, net monetary benefit with a QALY valued at £30,000 (shown in blue text); QALY, quality-adjusted life year; VoD, Vaccination-on-Diagnosis; VaN, Vaccination-according-to-partner-Notification; VaH(1yr), Vaccination-according-to-History (diagnosed in last year); VaH(2yr), Vaccination-according-to-History (diagnosed in last 2 years); VaR, Vaccination-according-to-Risk.

3 Supplementary Note 3: Alternative vaccine-sentiment scenario, with some unwilling to be vaccinated

3.1 Vaccination-status strata (Supplementary Figure 9)

In the scenario presented in the paper, all individuals have the same probability of accepting vaccination, which we have previously labeled “All-willing”[5]. We also consider an alternative scenario (which we have previously labeled “Some-unwilling”[5]) in which a proportion h of individuals are unwilling to be vaccinated, while the rest of the population are willing to be vaccinated and accept the first dose the first time it is offered (i.e. r_1 = 1), with uptake of the second primary dose (r_2) and of the booster dose (r_b) remaining the same as in our main scenario. We set the proportion h who are unwilling to be vaccinated to be equal to (1-r_1) in the “All-willing” scenario, to obtain equal initial rates of vaccination in the two scenarios[5].

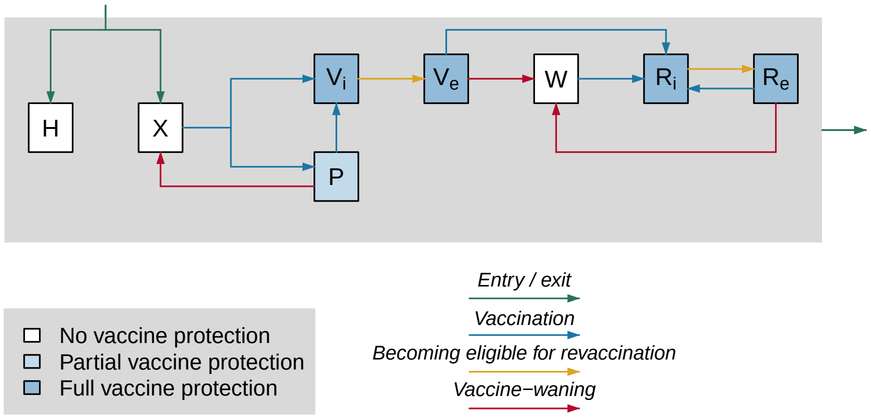

Supplementary Figure 9: Diagram of vaccination-status strata in model with some unwilling to be vaccinated. Stratification of the population according to vaccination status (unwilling (H), unvaccinated but willing (X), partially-vaccinated (P), fully-vaccinated (split into those who are ineligible for revaccination due to recent vaccination, V_i, and those who are eligible for revaccination, V_e), waned (W), and revaccinated (split into those who are ineligible due to recent revaccination, R_i, and those who are eligible for another revaccination, R_e). Individuals enter the sexually-active population into either the unwilling stratum (H) or the unvaccinated but willing stratum (X), and leave the population through ageing from any stratum. Individuals in H never accept vaccination, so remain in H throughout their time in the population. Individuals in stratum X who receive vaccination enter a vaccine-protected stratum (P or V), while those who decline vaccination remain in the unvaccinated stratum. Full primary vaccination comprises 2 doses, with individuals who receive both doses entering the fully-vaccinated (V_i) stratum and those who receive 1 dose entering the partially-vaccinated (P) stratum. Fully-vaccinated individuals become eligible for single-dose booster vaccination after a period of time, entering V_e. When vaccine protection wanes, partially-vaccinated individuals return to the unvaccinated stratum (X), while fully-vaccinated individuals enter the waned (W) stratum. Individuals in W are eligible for single-dose booster vaccination, with those who accept entering the revaccinated (R_i) stratum, with R strata having the same level of protection as V strata. Revaccinated individuals become eligible again for single-dose booster vaccination after a period of time, entering R_e.

3.2 Health-economic analyses (Supplementary Figure 10, Supplementary Tables 12 & 13)

Supplementary Figure 10a shows net monetary benefit boxplots, and Supplementary Figure 10b,c shows the probability that vaccination is cost-effective at different costs per dose administered, under the two vaccine-sentiment scenarios. Compared with the main ("All-willing") scenario, under the alternative ("Some-unwilling") scenario all vaccination strategies have lower NMB, as fewer doses are administered over 10 years, meaning fewer cases are averted. At 20% protection, $\text{NMB}_{£30k} > 0$ with $\geq$ 90% probability if vaccination costs $\leq$£19/dose administered for VoD, and $\leq$£23/dose administered for VaR, with all other considered strategies lying between these two values. At 40% protection, $\text{NMB}_{£30k} > 0$ with $\geq$ 90% probability if vaccination costs $\leq$£42/dose administered for VoD, and $\leq$£51/dose administered for VaR, with all other considered strategies lying between these two values.

Supplementary Tables 12 and 13 show health-economic analysis in a scenario where some individuals are unwilling to be vaccinated. Compared to our main scenario, all strategies have lower NMB. However, all strategies are as or more likely to be cost-effective (NMB $>$ 0) than our main analysis, and in all cases all strategies have over 50% probability that $\text{NMB}_{£20k} > 0$ and over 90% probability that $\text{NMB}_{£30k} > 0$, consistent with our previous finding that no minimum level of uptake is required for cost-effectiveness[5].

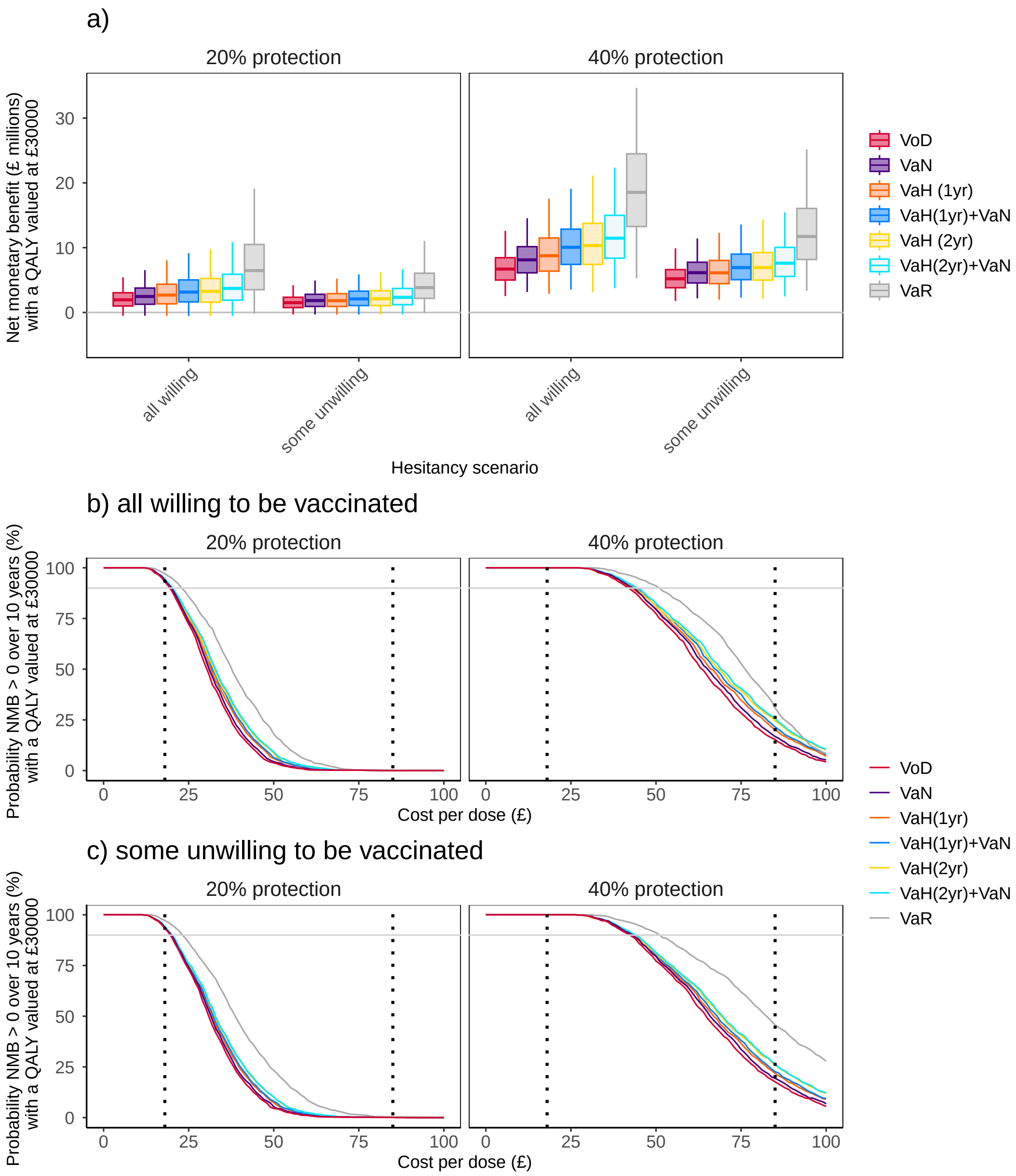

Supplementary Figure 10: Cost-effectiveness of vaccination, comparing vaccine-sentiment scenarios. Vaccination strategies considered are Vaccination-on-Diagnosis (VoD); Vaccination-according-to-partner-Notification (VaN); Vaccination-according-to-History (VaH) offering vaccination to those with a diagnosis in the last year [VaH(1yr)] or in the last two years [VaH(2yr)]; VaN combined with VaH(1yr) or VaH(2yr); and Vaccination-according-to-Risk (VaR). The left and right columns show results for vaccines providing 20% and 40% protection after two-dose primary vaccination, respectively. Primary vaccination has a 1.5-year duration of protection, and booster vaccination has a 3-year duration of protection. Section a) box plots of the net monetary benefit of each strategy over 10 years for a vaccine costing £18/dose administered and with a QALY valued at £30,000, under the scenario presented in the main paper where all individuals are willing to be vaccinated and under an alternative scenario where some individuals are unwilling to be vaccinated. Box plot whiskers indicate the 2.5^{th} and 97.5^{th} centiles, boxes the 25th and 75th centiles, and the central line the median (50th centile). Sections b,c) probability that vaccination is cost-effective (i.e. net monetary benefit is positive) at different costs per dose administered, with a QALY valued at £30,000, under b) the "All-willing" vaccine-sentiment scenario (presented in the main paper) or c) the "Some-unwilling" vaccine-sentiment scenario (in which a proportion of individuals never accept vaccination). All plots use 1000 sets of sampled epidemiological and health-economic parameters.

Supplementary Table 12: Health-economic analysis of vaccination of MSM in England, in an alternative scenario in which some MSM are unwilling to be vaccinated, over 10 years under different pragmatic vaccination strategies aiming to vaccinate high-risk MSM, for a vaccine with 20% protection after two doses, a 1.5-year duration of protection after primary vaccination, and a 3-year duration of protection after booster vaccination. Results are mean values and 95% credible intervals (CrIs) of simulations comparing each vaccination strategy against no vaccination, using 1000 sets of sampled epidemiological and health-economic parameters. All values are discounted at 3.5% per annum except where stated (cases averted and vaccine doses administered have both discounted and undiscounted numbers reported). Note that at £18/dose administered each strategy saves more money and gains more QALYs than the one above and therefore dominates the one above.

Strategy	Cases averted (thousands)		Testing & Treatment costs saved (£M)	QALYs gained	Vaccine doses administered (thousands)		£18/dose				£85/dose			
	Undisc.	Disc.			Undisc.	Disc.	Vaccination costs incurred (£M)	Net costs saved (£M)	NMB$_{£20k}$ (£M)<br>NMB$_{£30k}$ (£M)	Prob NMB$_{£20k}$>0<br>Prob NMB$_{£30k}$>0	Vaccination costs incurred (£M)	Net costs saved (£M)	NMB$_{£20k}$ (£M)<br>NMB$_{£30k}$ (£M)	Prob NMB$_{£20k}$>0<br>Prob NMB$_{£30k}$>0
VoD	23.4<br>(12.1, 37.8)	19.1<br>(9.9, 30.8)	3.1<br>(1.6, 5.2)	22.4<br>(10.7, 40.7)	137.2<br>(118.1, 159.4)	118.2<br>(102, 137.1)	2.1<br>(1.8, 2.5)	0.9<br>(-0.6, 3.1)	1.4<br>(-0.4, 3.8)<br>1.6<br>(-0.3, 4.2)	90.8%<br>93.3%	10.1<br>(8.7, 11.7)	-7<br>(-9.4, -4.6)	-6.5<br>(-9.1, -3.9)<br>-6.3<br>(-8.9, -3.5)	0%<br>0.1%
VaN	27.4<br>(14.2, 44.3)	22.4<br>(11.6, 36.2)	3.6<br>(1.8, 6.1)	26.2<br>(12.6, 48.1)	157.9<br>(135.3, 184.2)	136.5<br>(116.8, 159.4)	2.5<br>(2.1, 2.9)	1.2<br>(-0.7, 3.8)	1.7<br>(-0.5, 4.5)<br>1.9<br>(-0.3, 4.9)	91.5%<br>93.7%	11.6<br>(9.9, 13.5)	-8<br>(-10.9, -5.1)	-7.5<br>(-10.5, -4.3)<br>-7.2<br>(-10.3, -3.9)	0%<br>0.1%
VaH(1yr)	27.4<br>(12.9, 46.8)	22.4<br>(10.6, 38.2)	3.6<br>(1.7, 6.3)	26.2<br>(11.7, 49.4)	153.7<br>(135.2, 176.3)	133.2<br>(117.3, 152.4)	2.4<br>(2.1, 2.7)	1.2<br>(-0.7, 4)	1.7<br>(-0.5, 4.8)<br>2<br>(-0.3, 5.2)	91.4%<br>93.5%	11.3<br>(10, 13)	-7.7<br>(-10.2, -5)	-7.2<br>(-9.9, -4.1)<br>-6.9<br>(-9.7, -3.7)	0.1%<br>0.1%
VaH(1yr)+ VaN	30.9<br>(15, 51.7)	25.3<br>(12.4, 42.2)	4.1<br>(1.9, 7.1)	29.6<br>(13.3, 56)	172.1<br>(149.9, 199.3)	149.3<br>(130.4, 172.4)	2.7<br>(2.3, 3.1)	1.4<br>(-0.8, 4.4)	2<br>(-0.5, 5.4)<br>2.3<br>(-0.3, 5.9)	91.6%<br>93.9%	12.7<br>(11.1, 14.7)	-8.6<br>(-11.7, -5.5)	-8<br>(-11.3, -4.5)<br>-7.7<br>(-11.1, -4)	0.1%<br>0.1%
VaH(2yr)	30.7<br>(13.9, 53.6)	25.1<br>(11.4, 43.8)	4.1<br>(1.8, 7.2)	29.4<br>(12.4, 57.1)	166.5<br>(145.6, 192.5)	144.6<br>(126.5, 166.8)	2.6<br>(2.3, 3)	1.4<br>(-0.7, 4.7)	2<br>(-0.5, 5.6)<br>2.3<br>(-0.3, 6.2)	91.8%<br>93.9%	12.3<br>(10.8, 14.2)	-8.2<br>(-11.1, -5.3)	-7.7<br>(-10.6, -4.2)<br>-7.4<br>(-10.5, -3.8)	0.1%<br>0.2%
VaH(2yr)+ VaN	33.8<br>(15.7, 57.9)	27.7<br>(12.9, 47.4)	4.5<br>(2, 7.9)	32.5<br>(13.9, 61.3)	183.3<br>(159, 212.6)	159.5<br>(138.6, 184.3)	2.9<br>(2.5, 3.3)	1.6<br>(-0.8, 5.1)	2.2<br>(-0.5, 6.1)<br>2.6<br>(-0.3, 6.7)	92.1%<br>94.2%	13.6<br>(11.8, 15.7)	-9.1<br>(-12.4, -5.6)	-8.4<br>(-11.9, -4.6)<br>-8.1<br>(-11.8, -4)	0.1%<br>0.2%
VaR	47.4<br>(18.1, 87.7)	38.8<br>(14.9, 71.9)	6.3<br>(2.3, 12)	45.5<br>(16.1, 90.9)	211.8<br>(178.9, 242)	185.5<br>(155.8, 212.5)	3.3<br>(2.8, 3.8)	2.9<br>(-0.6, 8.2)	3.8<br>(-0.2, 10.1)<br>4.3<br>(-0.1, 11)	96.1%<br>97.2%	15.8<br>(13.2, 18.1)	-9.5<br>(-12, -5.6)	-8.6<br>(-11.6, -3.9)<br>-8.1<br>(-11.3, -3.1)	0.2%<br>0.3%

Abbreviations: Undisc., Undiscounted; Disc., Discounted; M, millions; NMB$_{£20k}$, net monetary benefit with a QALY valued at £20,000; NMB$_{£30k}$, net monetary benefit with a QALY valued at £30,000 (shown in blue text); QALY, quality-adjusted life year; VoD, Vaccination-on-Diagnosis; VaN, Vaccination-according-to-partner-Notification; VaH(1yr), Vaccination-according-to-History (diagnosed in last year); VaH(2yr), Vaccination-according-to-History (diagnosed in last 2 years); VaR, Vaccination-according-to-Risk.

Supplementary Table 13: Health-economic analysis of vaccination of MSM in England, in an alternative scenario in which some MSM are unwilling to be vaccinated, over 10 years under different pragmatic vaccination strategies aiming to vaccinate high-risk MSM, for a vaccine with 40% protection after two doses, a 1.5-year duration of protection after primary vaccination, and a 3-year duration of protection after booster vaccination. Results are mean values and 95% credible intervals (CrIs) of simulations comparing each vaccination strategy against no vaccination, using 1000 sets of sampled epidemiological and health-economic parameters. All values are discounted at 3.5% per annum except where stated (cases averted and vaccine doses administered have both discounted and undiscounted numbers reported). Note that at £18/dose administered each strategy saves more money and gains more QALYs than the one above and therefore dominates the one above.

Strategy	Cases averted (thousands)		Testing & Treatment costs saved (£M)	QALYs gained	Vaccine doses administered (thousands)		£18/dose				£85/dose			
	Undisc.	Disc.			Undisc.	Disc.	Vaccination costs incurred (£M)	Net costs saved (£M)	NMB$_{£20k}$ (£M)<br>NMB$_{£30k}$ (£M)	Prob NMB$_{£20k}$>0<br>Prob NMB$_{£30k}$>0	Vaccination costs incurred (£M)	Net costs saved (£M)	NMB$_{£20k}$ (£M)<br>NMB$_{£30k}$ (£M)	Prob NMB$_{£20k}$>0<br>Prob NMB$_{£30k}$>0
VoD	46<br>(25.9, 71)	37.6<br>(21.3, 58.1)	6.1<br>(3.3, 9.8)	44.1<br>(22.3, 78)	129.8<br>(109.4, 151.9)	112.1<br>(94.6, 131.2)	2<br>(1.7, 2.4)	4<br>(1.1, 7.9)	4.9<br>(1.6, 9.2)<br>5.4<br>(1.8, 9.9)	100%<br>100%	9.5<br>(8, 11.1)	-3.5<br>(-6.9, 0.7)	-2.6<br>(-6.4, 2)<br>-2.1<br>(-6.1, 2.7)	12.5%<br>17.6%
VaN	53.7<br>(30.3, 81.8)	43.9<br>(24.9, 66.9)	7.1<br>(3.9, 11.4)	51.4<br>(26.4, 91)	148.4<br>(124.9, 175.7)	128.5<br>(108.3, 151.4)	2.3<br>(2, 2.7)	4.8<br>(1.4, 9.2)	5.8<br>(2, 10.7)<br>6.3<br>(2.2, 11.4)	100%<br>100%	10.9<br>(9.2, 12.9)	-3.8<br>(-7.9, 0.9)	-2.8<br>(-7.3, 2.4)<br>-2.3<br>(-7, 3.3)	13.9%<br>19.4%
VaH(1yr)	54<br>(28.1, 87.7)	44.2<br>(23.1, 71.9)	7.1<br>(3.6, 12)	51.8<br>(24.6, 94.6)	145.5<br>(127.5, 168.7)	126.3<br>(111.1, 145.7)	2.3<br>(2, 2.6)	4.9<br>(1.2, 9.9)	5.9<br>(1.8, 11.4)<br>6.4<br>(2, 12.3)	100%<br>100%	10.7<br>(9.4, 12.4)	-3.6<br>(-7.6, 1.4)	-2.6<br>(-7, 3)<br>-2<br>(-6.7, 3.9)	16.6%<br>21.8%
VaH(1yr)+ VaN	60.6<br>(32.2, 96.6)	49.7<br>(26.5, 79.1)	8<br>(4.1, 13.3)	58.2<br>(28.4, 106.3)	161.7<br>(139.3, 188.9)	140.6<br>(121.8, 163.8)	2.5<br>(2.2, 2.9)	5.5<br>(1.5, 10.9)	6.6<br>(2, 12.6)<br>7.2<br>(2.3, 13.6)	100%<br>100%	12<br>(10.4, 13.9)	-3.9<br>(-8.6, 1.6)	-2.8<br>(-7.8, 3.4)<br>-2.2<br>(-7.5, 4.4)	17.5%<br>22.6%
VaH(2yr)	60.7<br>(30, 101)	49.8<br>(24.7, 82.9)	8<br>(3.8, 13.8)	58.3<br>(26.9, 109.4)	157.6<br>(138.5, 182.8)	137.2<br>(120.7, 158.3)	2.5<br>(2.2, 2.8)	5.6<br>(1.3, 11.4)	6.7<br>(1.8, 13.2)<br>7.3<br>(2.1, 14.3)	100%<br>100%	11.7<br>(10.3, 13.5)	-3.6<br>(-8.1, 2.3)	-2.5<br>(-7.5, 4.1)<br>-1.9<br>(-7.2, 5.1)	20.4%<br>26.2%
VaH(2yr)+ VaN	66.6<br>(33.9, 108.2)	54.6<br>(27.9, 88.9)	8.8<br>(4.4, 14.9)	64<br>(30.3, 116.6)	172.3<br>(149.4, 201.4)	150.3<br>(130.8, 175.5)	2.7<br>(2.4, 3.2)	6.1<br>(1.5, 12.3)	7.4<br>(2.1, 14.3)<br>8<br>(2.5, 15.5)	100%<br>100%	12.8<br>(11.1, 14.9)	-4<br>(-9.1, 2.3)	-2.7<br>(-8.3, 4.3)<br>-2<br>(-7.9, 5.4)	20.5%<br>26.4%
VaR	97.5<br>(40.1, 172.1)	79.8<br>(32.8, 140.6)	12.9<br>(5.1, 23.5)	93.4<br>(35.5, 181.9)	207.2<br>(176.8, 234.7)	181.6<br>(153.6, 206.9)	3.3<br>(2.8, 3.7)	9.6<br>(2.2, 20)	11.5<br>(3, 23.4)<br>12.4<br>(3.3, 25.2)	100%<br>100%	15.4<br>(13.1, 17.6)	-2.6<br>(-8.4, 6.5)	-0.7<br>(-7.6, 9.6)<br>0.2<br>(-7.2, 11.6)	38.5%<br>45.6%

Abbreviations: Undisc., Undiscounted; Disc., Discounted; M, millions; NMB$_{£20k}$, net monetary benefit with a QALY valued at £20,000; NMB$_{£30k}$, net monetary benefit with a QALY valued at £30,000 (shown in blue text); QALY, quality-adjusted life year; VoD, Vaccination-on-Diagnosis; VaN, Vaccination-according-to-partner-Notification; VaH(1yr), Vaccination-according-to-History (diagnosed in last year); VaH(2yr), Vaccination-according-to-History (diagnosed in last 2 years); VaR, Vaccination-according-to-Risk.

Supplementary References

[1] British National Formulary. Meningococcal Group B Vaccine (rDNA, Component, Absorbed). Available from: https://bnf.nice.org.uk/medicinal-forms/meningococcal-group-b-vaccine-rdna-component-adsorbed.html.

[2] NHS England. Human Papilloma Virus (HPV) Immunisation Programme for Men that Have Sex with Men (MSM) 2018/19. Available from: https://www.england.nhs.uk/wp-content/uploads/2019/01/hpv-msm-faqs-for-hcp.pdf (2019).

[3] Otto, J. & Kahle, D. ggdensity: Interpretable Bivariate Density Visualization with 'ggplot2' (2023). R package version 1.0.0.900, https://github.com/jamesotto852/ggdensity/.

[4] Whittles, L. K., Didelot, X. & White, P. J. Public health impact and cost-effectiveness of gonorrhoea vaccination: an integrated transmission-dynamic health-economic modelling analysis. *The Lancet Infectious Diseases* **22**, 1030–1041 (2022).

[5] Nikitin, D., Whittles, L. K., Imai-Eaton, J. W. & White, P. J. Cost-effectiveness of 4CMenB Vaccination Against Gonorrhea: Importance of Dosing Schedule, Vaccine Sentiment, Targeting Strategy, and Duration of Protection. *The Journal of Infectious Diseases* **231**, 71–83 (2025).

[6] Insitute of Medicine. *Vaccines for the 21st century: a tool for decisionmaking"* (National Academies Press, 2000).

[7] Li, Y. *et al.* The estimated lifetime quality-adjusted life-years lost due to chlamydia, gonorrhea, and trichomoniasis in the United States in 2018. *The Journal of Infectious Diseases* **227**, 1007–1018 (2023).

[8] Ärnlöv, J. *et al.* Global burden of 87 risk factors in 204 countries and territories, 1990–2019: a systematic analysis for the Global Burden of Disease Study 2019. *The Lancet* **396**, 1223–1249 (2020).
